# Elemental analyses reveal distinct mineralization patterns in radular teeth of various molluscan taxa

**DOI:** 10.1038/s41598-022-11026-w

**Published:** 2022-05-07

**Authors:** Wencke Krings, Jan-Ole Brütt, Stanislav N. Gorb

**Affiliations:** 1 Department of Behavioral Biology, Institute of Cell and Systems Biology of Animals, Universität Hamburg, Martin-Luther-King-Platz 3, 20146 Hamburg, Germany; 2 Department of Mammalogy and Palaeoanthropology, Leibniz Institute for the Analysis of Biodiversity Change, Martin-Luther-King-Platz 3, 20146 Hamburg, Germany; 3 Department of Functional Morphology and Biomechanics, Zoological Institute, Christian-Albrechts-Universität Zu Kiel, Am Botanischen Garten 9, 24118 Kiel, Germany

**Keywords:** Evolutionary ecology, Biomechanics, Structural biology

## Abstract

The molluscan phylum is the second specious animal group with its taxa feeding on a variety of food sources. This is enabled by the radula, a chitinous membrane with embedded teeth, one important autapomorphy. Between species, radulae can vary in their morphology, mechanical, and chemical properties. With regard to chemical composition, some taxa (Polyplacophora and Patellogastropoda) were studied extensively in the past decades, due to their specificity to incorporate high proportions of iron, calcium, and silicon. There is, however, a huge lack of knowledge about radular composition in other taxa. The work presented aims at shedding light on the chemistry by performing energy-dispersive X-ray spectroscopy analyses on 24 molluscan species, thereof two Polyplacophora, two Cephalopoda, and 20 Gastropoda, which was never done before in such a comprehensiveness. The elements and their proportions were documented for 1448 individual, mature teeth and hypotheses about potential biomineralization types were proposed. The presented work additionally comprises a detailed record on past studies about the chemical composition of molluscan teeth, which is an important basis for further investigation of the radular chemistry. The found disparity in elements detected, in their distribution and proportions highlights the diversity of evolutionary solutions, as it depicts multiple biomineralization types present within Mollusca.

## Introduction

The radula is the molluscan autapomorphy for food gathering and processing. Overall, its general shape reflects the deep molluscan phylogeny, whereas the fine morphology and chemical composition seem to be usually species-specific, serving as taxonomic character. All parameters are also, however, considered as adaptations to different ingesta types (food type, minerals, the substrate where the food is collected from, etc.) related to the ecological niche of particular species.

In most molluscan classes, the radula consists of a membrane with rows of embedded teeth, which form, together with underlain odontophoral cartilages, surrounding muscles, alary processus, and in some taxa also the jaw, the feeding apparatus, i.e. the buccal mass. The radula itself is continuously regrown by secretion of over- and underlain epithelia situated in the 'radular sac', before it is maturated in the ‘maturation zone’ and finally enters the ‘working zone’, where teeth interact with the ingesta [e.g.^1–3^].

Past studies related the radular morphology and general buccal mass anatomy with the ingesta preferred [e.g.^4–13^], supplemented by phenotypic plasticity studies addressing the morphological answers to shifts in the diet [e.g.^14–18^]. However, the radular tooth shape is not always a good indicator for trophic specializations^19^, since morphology seems to be more phylogenetically fixated and rather less adaptive in some cases^12^. Additionally, experimental approaches revealed that not all teeth necessarily interact with the ingesta, but still contribute to the overall radular function by e.g. reinforcing the spanned radula^20,21^, which indicates that tooth function and the identification of potential trophic adaptations is more complex than it has been thought before.

The mechanical properties (e.g. stiffness and hardness) of the radular teeth material can be considered as adaptive to certain ingesta types. This is well investigated for the dominant lateral teeth of Polyplacophora and patelliform gastropods, which are of high stiffness and hardness as adaptations to feeding on algae covering rocks^22–28^. For these taxa, the mechanical parameters have their origin in the architecture of the organic components (matrix of alpha chitin with associated proteins) as e.g. fiber orientation or density, the distinct folding or bonding conditions of the chitin, and the amount/distribution of the inorganic components (iron, silicon, and calcium) [e.g.^23,25,27–32^; for reviews see^33–36^].

This direct relationship between ingesta and tooth mechanical properties was recently also described for closely related paludomid gastropods from Lake Tanganyika; here species feed either on algae covering sand, mud, rock, or multiple surfaces^12,37–40^. The origins of the differences in hardness and stiffness remained, however, enigmatic, as radular teeth are completely understudied with regard to their structural organization and chemical composition outside the polyplacophoran or limpet realm. Only very few past studies focused on the radular composition in non-patelliform gastropods [e.g.^41–43^], cephalopods^43^, or scaphopods^43–45^.

We thus aim here on shedding some light on the chemical composition of radular teeth. We performed elemental analyses using energy disperse x-ray spectroscopy (EDX, EDS), overall on 1448 mature teeth of 24 species, thereof two Polyplacophora, two Cephalopoda, and 20 Gastropoda species. For the gastropods, we have chosen representative taxa from the Vetigastropoda, Neritimorpha, Heterobranchia, Caenogastropoda, and one Patellogastropoda for comparison. For the Caenogastropoda, we included six paludomid species from Lake Tanganyika, which were previously studied extensively with regard to the radular tooth mechanical properties, because one goal was the determination of the origin of heterogeneities. The elements incorporated in the mature teeth were identified, documented, and compared between the species studied. The aim of this project is the identification of similarities and differences in tooth mineralization between the molluscan taxa, which was never done before for such an elevated quantity of taxa. The results are compared and interpreted against the background of previous studies on the chemical composition of radular teeth and functional adaptations of different radulae.

## Results

### Radular morphology and types

Overall, the here analysed radulae are highly distinct in morphology, size, and quantity of tooth rows (see Table 1 and Supplementary Figs. 1–24 for each species). However, these differences are rather interspecific, as the selected specimens, which were of similar body size for each species, show only little intraspecific variability in the radular parameters studied here.

**Table 1:** Systematic position of the taxa studied, list of specimens with collection number, locality and date of collection or fixation.

Class	Subclass	Species	Specimens				Ecology			Radula						
			*N* of specimens studied with EDX + SEM	Collection number	Source or locality of collection	Date of collection or fixation	Food or substrate	Reference for ecology	Ingesta category	Radular type	Radular formula	Specimen no	Length, µm	Width, µm	Area, µm^2^	*N* of tooth rows
Polyplacophora	Chitonida	*Lepidochitona cinerea* (Linnaeus, 1767)	3 + 1	ZMH 154653	North Sea, at Husum, Germany	Autumn 2019	Algae from solid substrate	^46^	Solid	Docogloss	1 + DT + 1 + C + 1 + DT + 1	1	3375	375	1,265,625	39
												2	3269	369	1,206,261	39
												3	3109	354	1,100,586	39
	Chitonida	*Acanthochitona fascicularis* (Linnaeus, 1767)	2 + 1	ZMH 122789	North Sea, at Roscoff, France	2018, 2019	Algae from solid substrate	^47^	Solid	Docogloss	1 + DT + 1 + C + 1 + DT + 1	1	7630	890	6,790,700	55
												2	7527	872	6,566,587	54
												3	7559	880	6,652,595	55
Cephalopoda	Oegopsida	*Histioteuthis spec.* d'Orbigny [in Férussac & d'Orbigny], 1841	2	ZMH 11623/999	NE Atlantic, 46°29′24’’N 027°14′18’’W -250 m	12.06.1982	Fish, crustacea, Cephalopoda	^48^	Soft to solid	Homodont	2 + 1 + C + 1 + 2	1	868	464	402,752	36
												2	857	459	393,363	36
	Myopsida	*Loligo vulgaris* Lamarck, 1798	2 + 1	ZMH 12279	Indonesia	Spring 2021	Fish, crustacea, Cephalopoda	^49^	Soft to solid	Homodont	2 + 1 + C + 1 + 2	1	6430	1480	9,516,400	45
												2	6531	1485	9,698,535	46
												3	6315	1369	8,645,235	45
Gastropoda	Patellogastropoda	*Patella vulgata* Linnaeus, 1758	2 + 1	ZMH 122790	North Sea, at Roscoff, France	30.09.2020	Algae from rocks, macro algae	^50^	Solid	Docogloss	3 + DT + 2 + 0 + 2 + DT + 3	1	36,634	949	34,765,666	195
												2	36,862	962	35,461,244	197
												3	35,925	958	34,416,150	197
	Vetigastropoda	*Rochia conus* (Gmelin, 1791)	2 + 1	ZMH 154624	Pet shop	Summer 2019	Algae/ plants from corals and rocks	www.sealifebase.ca	Solid	Rhipidogloss	∞ + 5 + C + 5 + ∞	1	6070	980	5,948,600	102
												2	6054	978	5,920,812	101
												3	6103	968	5,907,704	102
		*Haliotis tuberculata* Linnaeus, 1758	2 + 1	ZMH 122791	Pet shop	Summer 2021	Macro algae	^51^	Medium	Rhipidogloss	∞ + DT + 2 + C + 2 + DT + ∞	1	15,945	3690	58,837,050	114
												2	15,763	3598	56,715,274	113
												3	15,899	3304	52,530,296	113
	Neritimorpha	*Vittina turrita* (Gmelin, 1791)	2 + 1	ZMH 154753	Pet shop	Summer 2020	solid substrates, but also porous ingesta	^52^	Medium to solid	Rhipidogloss, neritinomorph	40 + 1 + 1 + C + 1 + 1 + 40	1	17,350	1210	20,993,500	150
												2	17,364	1205	20,923,620	151
												3	18,023	1305	23,520,015	149
	Caenogastropoda	*Lavigeria grandis* (Smith, 1881)	2 + 1	ZMH 150020/999	Zambia 08°43′25’’S 31°09′00’’E	30.11.2017	Algae from rocks	^53–56^	Solid	Taeniogloss	2 + 1 + C + 1 + 2	1	8120	900	7,308,000	85
												2	8109	886	7,184,574	83
												3	7906	749	5,921,594	85
		*Lavigeria nassa* (Woodward, 1859)	2 + 1	ZMH 119369/999	Zambia 08°29′23 ‘’S 30°28′46’’E	09.09.2016	Algae from rocks	^53,54,57–59^; personal comment from collector (Heinz Büscher)	Solid	Taeniogloss	2 + 1 + C + 1 + 2	1	5160	430	2,218,800	112
												2	5136	426	2,187,936	112
												3	5004	435	2,176,740	110
		*Paramelania damoni* (Smith, 1881)	2 + 1	ZMH 150023/999	Zambia 08°34 ‘09 ‘’S 31°45 ‘02 ‘’E	05.05.2018	Algae from rocks and sand	^53–56,58–62^	Soft to solid	Taeniogloss	2 + 1 + C + 1 + 2	1	2060	388	799,280	98
												2	2054	376	772,304	97
												3	1786	–	–	–
		*Cleopatra johnstoni* Smith, 1893	2 + 1	ZMB 220.102b	Zambia 09°20′866’S 28°43′886’E	19.12.2000	Algae from sand and mud	Unpublished work, personal comment from collector (Matthias Glaubrecht)	Soft	Taeniogloss	2 + 1 + C + 1 + 2	1	2082	349	726,618	70
												2	2076	347	720,372	71
												3	–	326	–	–
		*Reymondia horei* (Smith, 1880)	2 + 1	ZMB 220.147	Tanzania Kigoma	26.02.1995	Algae from rocks	^53–56,61,62^; personal comment from collectors (Heinz Büscher and Matthias Glaubrecht)	Solid	Taeniogloss	2 + 1 + C + 1 + 2	1	9240	820	7,576,800	176
												2	9342	831	7,763,202	178
												3	9108	803	7,313,724	176
		*Spekia zonata* (Woodward, 1859)	2 + 1	ZMB 220.077	Zambia 08°45 ‘547 ‘’S 31°05 ‘825 ‘’E	12.02.2000	Algae from rocks	^53–56,58,60–63^; personal comment from collectors (Heinz Büscher and Matthias Glaubrecht)	Solid	Taeniogloss	2 + 1 + C + 1 + 2	1	5660	560	3,169,600	138
												2	5680	571	3,243,280	139
												3	5589	577	3,224,853	138
		*Faunus ater* (Linnaeus, 1758)	2 + 1	ZMH 154630	Pet shop	Summer 2019	? found on soft and solid substrate	^64,65^	Soft to solid ?	Taeniogloss	2 + 1 + C + 1 + 2	1	11,480	510	5,854,800	170
												2	11,502	505	5,808,510	169
												3	11,899	489	5,818,611	170
		*Littorina littorea* (Linnaeus, 1758)	2 + 1	ZMH 154633	North Sea, at Husum, Germany	Autumn 2019	Algae, fleshy macro algae, also from rocks	^66–69^	Medium to solid	Taeniogloss	2 + 1 + C + 1 + 2	1	23,180	370	8,576,600	280
												2	23,195	376	8,721,320	281
												3	–	384	–	–
		*Paludomus siamensis* Blanford, 1903	2 + 1	ZMB 220.234	Thailand, Kanchanaburi, 14°26,3’N 98°51,0’E	08.02.2001	Not known	?	?	Taeniogloss	2 + 1 + C + 1 + 2	1,2,3	–	–	–	–
		*Anentome helena* (von dem Busch, 1847)	2 + 1	ZMH 122792	Pet shop	Summer 2019	Gastropoda, fish eggs, shrimps, carrion	^70,71^	Soft to solid	Stenogloss	1 + C + 1	1	2097	244	511,668	61
												2	2102	241	506,582	60
												3	2189	261	571,329	61
		*Buccinum undatum* Linnaeus, 1758	2 + 1	ZMH 122793	Biologische Anstalt Helgoland, Germany	May 2021	Polychaeta, fish eggs, bivalves, carrion, etc	^72^	Soft to solid	Stenogloss	1 + C + 1	1	9660	1420	13,717,200	59
												2	9641	1396	13,458,836	60
												3	9701	1486	14,415,686	64
	Heterobranchia	*Onchidoris bilamellata* (Linnaeus, 1767)	2 + 1	ZMH 122794	Biologische Anstalt Helgoland, Germany	May 2021	Soft parts of barnacles	^4,73^	Medium	–	1 + DT + TM + DT + 1	1	2060	447	920,820	34
												2	2081	461	959,341	34
												3	–	474	–	–
		*Aeolidia papillosa* (Linnaeus, 1761)	2 + 1	ZMH 122795	Biologische Anstalt Helgoland, Germany	May 2021	Sea anemone	^74–76^	Soft	–	DC	1	1460	410	598,600	9
												2	1471	420	617,820	9
												3	1507	429	646,503	10
		*Polycera quadrilineata* (Müller, 1776)	2 + 1	ZMH 122796	Biologische Anstalt Helgoland, Germany	May 2021	Encrusted Bryozoa	^77^	Medium	–	1 + 1 + TM + 1 + 1	1	2075	726	1,506,450	12
												2	2082	731	1,521,942	12
												3	–	–	–	–
		*Doris pseudoargus* Rapp, 1827	2 + 1	ZMH 122797	Biologische Anstalt Helgoland, Germany	May 2021	Porifera	^78,79^	Medium	Isodont	∞ + ∞ + C + ∞ + ∞	1	2350	2520	5,922,000	26
												2	2461	2580	6,349,380	28
												3	–	–	–	–
		*Cornu aspersum* (Müller, 1774)	2 + 1	ZMH 150.005	Pet shop	2018	Various plant types	www.cabi.org/isc/ datasheet/26,821	Soft to solid	Isodont	∞ + ∞ + C + ∞ + ∞	1	8000	3000	24,000,000	171
												2	8123	3004	24,401,492	180
												3	8206	3208	26,324,848	179

The ingesta preferred, if known, and the ingesta categories assigned in this study are listed. Radular parameters, i.e. type, formula, length, width, area, and quantity of tooth rows, are documented for each specimen. C, central tooth; DC, dominant central tooth; DT, dominant lateral tooth or lateral tooth II; TM, thickened membrane, potentially reduced central tooth.

Based on the arrangement and quantity of certain tooth shapes, the radulae of the studied species could be assigned to the following published radular type categories (see Table 1): those of the Polyplacophora and Patellogastropoda to the docoglossan, those of the Cephalopoda to the homodont, those of the Vetigastropoda and Neritimorpha to the rhipidoglossan (special type ‘neritinomorph’ for *Vittina turrita*), those of the Caenogastropoda to the taenioglossan or stenoglossan, and those of the Heterobranchia *Doris pseudoargus* and *Cornu aspersum* to the isodont type. Radulae of *Onchidoris bilamellata*, *Aeolidia papillosa,* and *Polycera papillosa* could not be assigned to existing categories due to their diverging morphologies. The categories usually reflect phylogeny, except for the docoglossan type, which was detected in Polyplacophora and Gastropoda (Patellogastropoda). Within Heterobranchia, specifically Nudibranchia (*O. bilamellata*, *A. papillosa*, *P. quadrilineata*, *D. pseudoargus*), we detect a rather large diversity of radular shapes and thus different types. In addition, radular formulae, based on the quantity and arrangement of teeth with a specific shape, were determined. These do not consistently reflect phylogeny, except for Polyplacophora, Cephalopoda, and each of the two large Caenogastropoda groups (Buccinoidea and Paludomidae), since selected radulae are rather diverging in their interspecific morphology.

*Histioteuthis spec.* possesses the shortest radula (< 1 mm), *Patella vulgata* the longest one (37 mm). *Faunus ater*, *Haliotis tuberculata*, *V. turrita*, and *Littorina littorea* show radulae that are of 10–23 mm length, all other species of 1.4–10 mm. Areas of radulae in *Buccinum undatum*, *V. turrita*, *C. aspersum*, and *P. vulgata* are rather large (14–35 mm^2^). The largest one was detected for *H. tuberculata* (59 mm^2^) and the smallest for *H. spec.* (0.39 mm^2^). Most species have less than 100 tooth rows, with *A. papillosa* possessing the smallest quantity (9 rows). The radulae of *Rochia conus*, *Lavigeria nassa*, *H. tuberculata*, *Spekia zonata*, *F. ater*, *C. aspersum*, *Reymondia horei*, and *P. vulgata* contain 100–200 rows. *V. turrita*’s radula possesses 149–151 rows, whereas *L. littorea*’s one has the highest number of rows (280–281). Length, area, and tooth row number do not seem to relate to the phylogeny as closer related species (e.g. the Paludomidae: *L. nassa, L. grandis*, *R. horei*, *S. zonata*, and *C. johstoni*) show large differences in these parameters.

### Ingesta

Overall, the preferred ingesta types (see Table 1) vary greatly within classes, especially within Vetigastropoda, Caenogastropoda, and Heterobranchia, and convergent adaptations to similar trophic categories are present. The two studied Polyplacophora species (*Acanthochitona fascicularis* and *Lepidochitona cinerea*), the Patellogastropoda *Patella vulgata*, the Vetigastropoda *Rochia conus*, the Caenogastropoda *Lavigeria grandis*, *L. nassa*, *Reymondia horei*, and *Spekia zonata* can be regarded as solid ingesta feeders. Neritimorpha *Vittina turrita* and Caenogastropoda *Littorina littorea* seem to forage on solid, but also medium hardness ingesta, the Vetigastropoda *Haliotis tuberculata* and the Heterobranchia *Onchidoris bilamellata*, *Polycera quadrilineata*, and *Doris pseudoargus* on ingesta of medium stiffness and hardness. The Caenogastropoda *Cleopatra johnstoni* and the Heterobranchia *Aeolidia papillosa* were the only species foraging on softer ingesta. The Cephalopoda *Histioteuthis spec.* and *Loligo vulgaris*, the Caenogastropoda *Paramelania damoni*, *Faunus ater*, *Anentome helena*, and *Buccinum undatum*, as well as the Heterobranchia *Cornu aspersum* seem to forage on the widest range of food types (soft to solid).

### Elements detected

**Figure 1:**
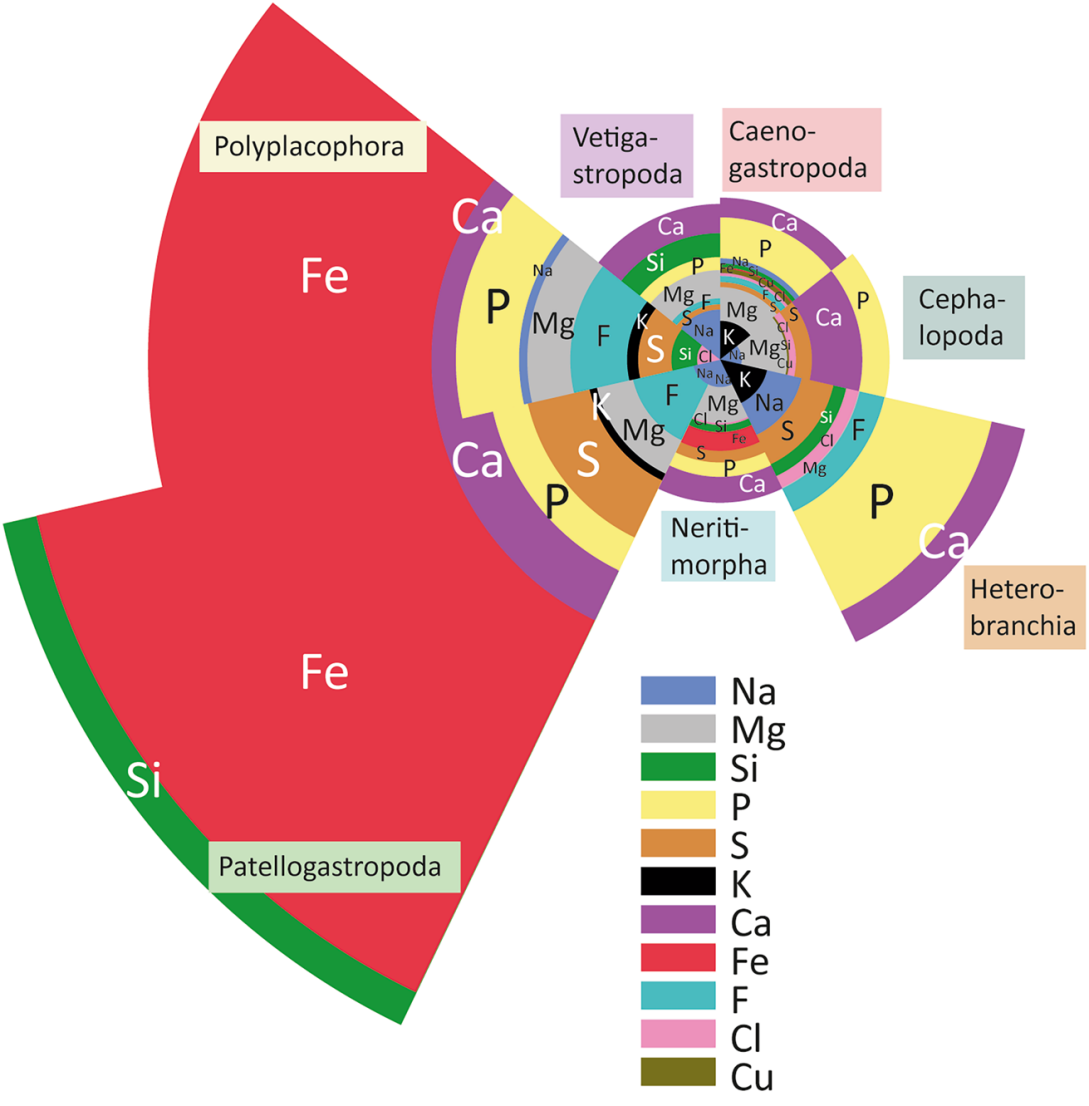
Elemental proportions of the species’ radular teeth, summarized for taxa (Patellogastropoda, Polyplacophora, Vetigastropoda, Caenogastropoda, Cephalopoda, Heterobranchia, Neritimorpha) to ease comparison in the radular mineral content.

**Figure 2:**
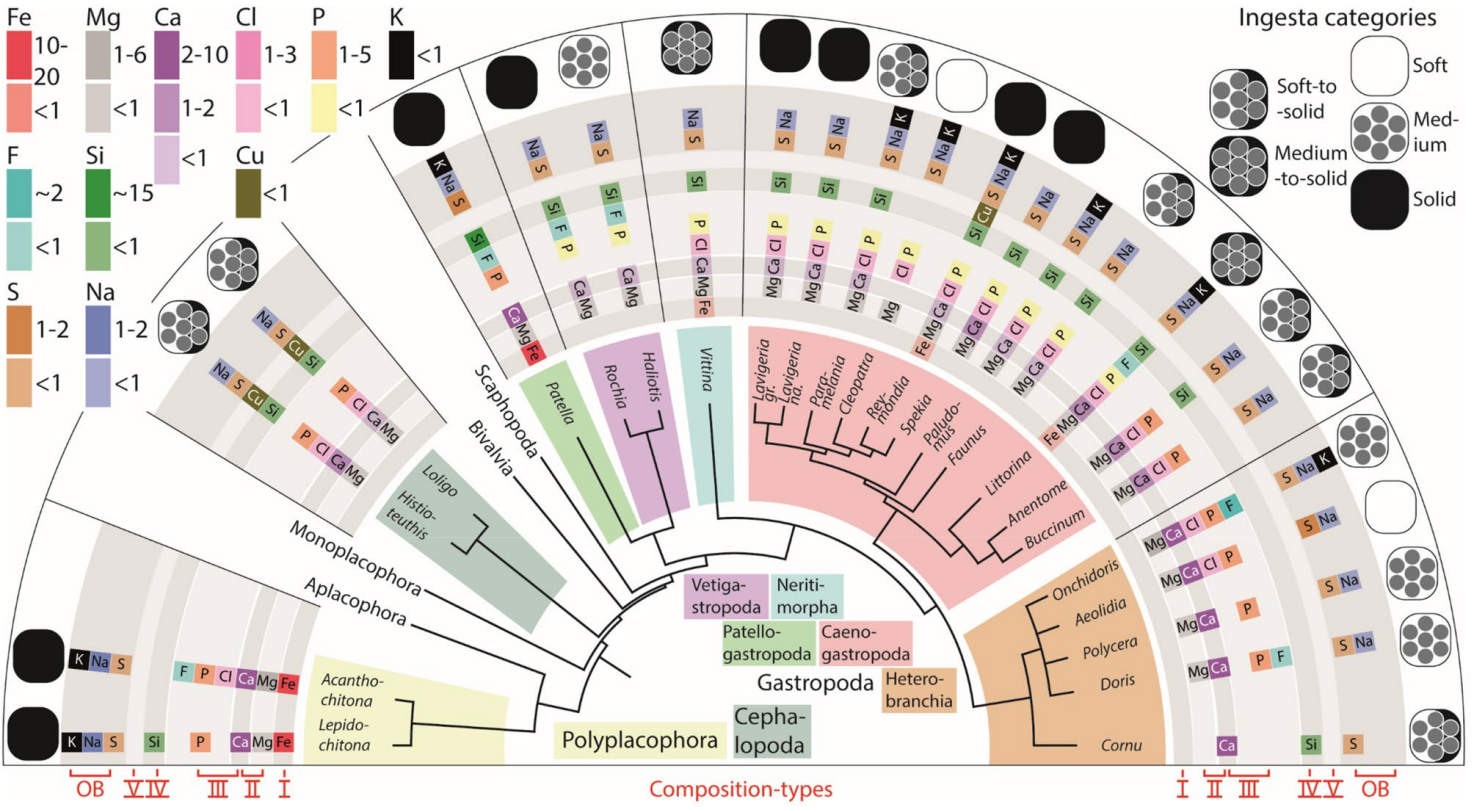
Elements detected, the mean atomic percent (represented by different colours), and assigned ingesta category (soft, medium, solid, soft to solid, medium to solid) for each species, sorted to the major molluscan groups (Polyplacophora, Cephalopoda, Gastropoda: Patellogastropoda, Vetigastropoda, Caenogastropoda, Neritimorpha, Heterobranchia). Elements are sorted to the defined composition-types (I, II, III, IV, V, OB).

Overall, the presence of many elements is rather puzzling and does not follow, in each case, the phylogeny (see Figs. 1 and 2; Supplementary Figs. 1–27; Supplementary Table 3 for mean, SD, sum of means, and N): S was detected in the radula of each studied species. Mg, Ca, Na, and P in most, but in *Cornu aspersum* no Mg, Na, and P and in *Cleopatra johnstoni* no Ca was found. Cl was determined for radulae of *Acanthochitona fascicularis*, both Cephalopoda, *Vittina turrita*, all Caenogastropoda, *Onchidoris bilamellata*, and *Aeolidia papillosa*. F was detected in *A. fascicularis*, *Patella vulgata*, both Vetigastropoda, *Littorina littorea*, *O. bilamellata*, and *Doris pseudoargus*. Si was found in *Lepidochitona cinerea*, both Cephalopoda, *P. vulgata*, both Vetigastropoda, *V. turrita*, all Caenogastropoda except *C. johnstoni* and *Buccinum undatum*, and in *Cornu aspersum*. Cu was determined for radulae of both Cephalopod species and *Reymondia horei*. K was found in both Polyplacophora, *P. vulgata*, *Paramelania damoni*, *C. johnstoni*, *R. horei*, *Paludomus siamensis*, *L. littorea*, and *O. bilamellata*.

### Elemental proportions

Summarizing all radulae studied (for mean, SD, and N see Supplementary Table 1), we detect that Fe is present in the highest proportions, followed by Si, Ca, P, F, Na, Mg, S, Cl, Cu, and finally K. Of the 1448 areas studied, most of them contained Ca, followed by P, Mg, S, Cl, Na, Si, F, Fe, Cu, and finally K.

Overall, the highest content of all analysed elements (sum, in atomic %, of the means of F, Na, Mg, Si, P, S, Cl, K, Ca, Fe, and Cu; see Supplementary Table 2 for values) was detected for the studied Patellogastropoda. This is followed by the Polyplacophora, Heterobranchia, Caenogastropoda, Cephalopoda, Vetigastropoda, and finally the Neritimorpha.

### Composition- and biomineralization-types

Overall, we detect that multiple composition- and biomineralization types are present within each individual species and also within individual teeth (see Fig. 2). Within Polyplacophora and Patellogastropoda we detected strong indications for the presence of the composition type I (containing Fe), II (Mg, Ca), III (Ca, P, Cl, F), IV (Si), OB (Na, S, K). In Cephalopoda, the types II, III, IV, V (Cu), and OB occur. In Vetigastropoda and Neritimorpha, the types II, III, IV, and OB were detected, in *Vittina turrita*—additionally the type I. In Caenogastropoda and Heterobranchia, the radular composition greatly varies between taxa. Overall, in Caenogastropoda, all composition types were found. However, in each species, the types II, III, and OB were always present, whereas Fe was only determined in *Reymondia horei* and *Littorina littorea*, Cu — in *R. horei*, and K — in *Paramelania damoni*, *Cleopatra johnstoni*, *R. horei*, *Faunus ater*, and *L. littorea*. *C. johnstoni* is the only species that seems to lack Ca. In Heterobranchia, the types II and OB were found in each species. The type III (apatite) is present in *Onchidoris bilamellata*, *Aeolidia papillosa*, *Polycera quadrilineata*, and *Doris pseudoargus*, but not in *Cornu aspersum,* as P, Cl, and F were not determined in this species. Si was only detected in *C. aspersum* and K in *O. bilamellata*.

### Ingesta versus radular morphology and elemental proportions

#### Morphology

The longest radulae were detected in species foraging on medium to solid ingesta, followed by solid-, medium-, soft-to-solid-, and finally soft-feeders with the shortest radulae (see Table 2 and Supplementary Figs. 28–30). The largest radular area was calculated for medium-, medium-to-solid-, soft-to-solid-, solid-, and finally for the soft-feeders with the smallest area. Species foraging on medium-to-solid ingesta possess the highest quantity of tooth rows, followed by solid-, soft-to-solid-, medium-, and finally with the least quantity of tooth rows the soft-feeders.

**Table 2:** Proportions of elements, radular length, area, quantity of tooth rows for the species foraging on certain ingesta types.

Parameter	Ingesta type														
	Soft			Soft to solid			Medium			Medium to solid			Solid		
	Mean	SD	*N*	Mean	SD	*N*	Mean	SD	*N*	Mean	SD	*N*	Mean	SD	*N*
Proportions of all elements, atomic %	1.88	2.80	64/64	2.99	2.40	348/348	5.58	6.24	164/164	3.97	2.39	122/122	6.33	12.50	688/688
Radular length, µm	1964.00	241.55	5	5901.28	3945.67	20	7704.29	6710.08	9	20,128.88	2923.74	5	7911.95	8319.09	24
Radular area, µm^2^	702,397	45,924	5	7,406,442	7,723,878	19	25,206,904	26,887,891	9	15,106,455	6,172,958	5	6,144,306	8,488,474	24
*N* of tooth rows	59	24	5	92	53	20	62	43	9	241	38	5	97	53	24
Fe-proportion, atomic %	0.00	0.00	0/64	0.00	0.00	0/348	0.00	0.00	0/164	0.34	0.15	62/122	13.69	10.17	120/688
Mg-proportion, atomic %	0.34	0.24	41/64	0.15	0.11	237/348	0.68	0.43	114/164	0.28	0.27	85/122	0.42	0.62	426/688
Ca-proportion, atomic %	3.11	2.49	12/64	1.22	1.27	317/348	3.15	3.22	150/164	1.12	1.03	118/122	1.84	1.80	561/688
P-proportion, atomic %	0.69	0.95	22/64	1.29	1.12	264/348	1.91	2.06	132/164	0.63	0.62	112/122	1.11	1.64	405/688
Cl-proportion, atomic %	0.26	0.43	27/64	0.42	0.41	256/348	0.98	0.27	6/164	0.16	0.18	66/122	0.21	0.15	248/688
F-proportion, atomic %	0.00	0.00	0/64	0.00	0.00	0/348	0.65	0.74	136/164	0.28	0.24	44/122	0.38	0.48	156/688
Si-proportion, atomic %	0.00	0.00	0/64	0.22	0.20	116/348	0.74	0.58	24/164	0.50	0.46	120/122	7.39	10.40	83/688
Cu-proportion, atomic %	0.00	0.00	0/64	0.19	0.12	94/348	0.00	0.00	0/164	0.00	0.00	0/122	0.33	0.17	52/688
S-proportion, atomic %	0.62	1.05	40/ 64	0.36	0.25	241/348	0.22	0.20	69/164	0.32	0.20	104/122	0.38	0.52	349/688
Na-proportion, atomic %	0.38	0.28	58/64	0.22	0.22	174/348	0.22	0.31	69/164	0.14	0.12	64/122	0.66	0.70	230/688
K-proportion, atomic %	0.02	0.01	14/64	0.08	0.08	24/348	0.09	0.08	10/164	0.09	0.08	6/122	0.39	0.40	40/688

N, quantity of teeth that contain the element, or quantity of radulae studied.

#### All elements

In general, we detect that radulae of species foraging on solid ingesta possess the highest proportions of all studied elements, followed by species foraging on medium, medium-to-solid, soft-to-solid, and finally species feeding on soft ingesta.

#### Composition-type I

The highest Fe-proportions (means) were detected in the exclusively solid-, followed by the medium-to-solid-feeders. No Fe was detected for all other ingesta types.

#### Composition-type II and III

The highest proportions of Mg were detected in species foraging on medium ingesta, followed by solid-, soft-, medium-to-solid-, and finally soft-to-solid-feeders. Ca was detected in the highest proportions in the medium-, followed by the soft-, solid-, soft-to-solid-, and medium-to-solid-feeders. P was mainly found in species feeding on medium ingesta, followed by soft-to-solid-, solid-, medium-to-solid-, and finally soft-feeders. Cl was detected in the highest proportions in medium-feeders, followed by species feeding on soft-to-solid, soft, solid, and finally medium-to-solid ingesta. The highest proportions of F were found in radulae of medium-, followed by solid-, and finally medium-to-solid-feeders. In soft and soft-to-solid-feeders, no F was found.

#### Composition-type IV

The highest Si-content was detected in solid-, followed by medium-, medium-to-solid-, and finally soft-to-solid-feeders. No Si was found in species feeding on soft ingesta.

#### Composition-type V

The highest Cu proportions were detected in the solid-feeder *Reymondia horei* and less in the cephalopods foraging on soft-to-solid ingesta. All other radulae seem to lack Cu.

#### OB

S was detected in the highest proportions in species feeding on soft, followed by solid, soft-to-solid, medium-to-solid, and finally medium ingesta. The highest Na-proportions were detected in solid-, followed by the soft-, soft-to-solid-, medium-, and finally medium-to-solid-feeders. K was detected in the highest proportions in solid-, followed by medium-, medium-to-solid-, soft-to-solid-, and finally soft-feeders.

#### Correlations between parameters

In some cases we could detect correlations (please see Supplementary Tables 4–16 for correlation coefficients). In general, radular area highly correlates with radular length and radular width. Additionally, in most cases, proportions of all elements highly correlate with each individual element.

For individual elemental proportions, we here only highlight some, but the picture is rather puzzling. When all species are pooled together (see Supplementary Table 4) Ca and Cl, F and Ca, Fe and Ca, P and Ca, P and Cl, Si and Ca, Si and Fe correlate highly. For all soft ingesta feeders pooled together (see Supplementary Table 5) Mg and Na, K and P, Ca and P, Cl and P, Cl and K, Cl and Ca highly correlate. For all soft-to-solid ingesta feeders pooled together (see Supplementary Table 6) K and P, K and S, Ca and K, Cl and P, Cl and K highly correlate. For all medium ingesta feeders pooled together (see Supplementary Table 7) S and Si, K and Mg, Ca and P, F and P, F and Ca, Cl and S highly correlate. For all medium-to-solid ingesta feeders pooled together (see Supplementary Table 8) Si and Na, Si and Mg, K and Na, Ca and P, Ca and K, F and P, F and K, F and Ca, Cl and P, Cl and Ca highly correlate. For all solid ingesta feeders pooled together (see Supplementary Table 9) Si and Mg, P and Si, S and Si, K and P, Ca and P, Cl and P correlate highly.

When the species studied are sorted by their taxonomic group we find, for most parameters no high correlations. However, for Caenogastropoda (see Supplementary Table 10) K and Si, F and P, F and K, F and Ca, Cl and P highly correlate. In Cephalopoda (see Supplementary Table 11) Cl and Ca correlate highly. In Heterobranchia (see Supplementary Table 12) K and Mg, Ca and P, F and P, Cl and Na are highly correlated. In Neritimorpha (see Supplementary Table 13) Si and Na, Si and Mg, Ca and P, Cl and P, Cl and Ca are highly correlated. In Patellogastropoda (see Supplementary Table 14) Si and Mg, P and Mg, S and P, K and Na, K and P, K and S, Ca and Mg, Ca and P highly correlate. In Polyplacophora (see Supplementary Table 15) P and Na, S and Mg, K and P, Fe and Si, F and K, Cl and Na are highly correlated. In Vetigastropoda (see Supplementary Table 16) S and Na highly correlate.

PCA on the individual parameters (elemental proportions, radular length, radular width, radular area, tooth rows) for all species pooled together detected no clustering (see Supplementary Fig. 31 A with highlighted systematic groups and B with highlighted ingesta categories).

## Discussion

A detailed list of previous studies aimed at determining the chemical composition of the molluscan radula is provided in Supplementary Table 17. Most of the previous research has been done on the Polyplacophora with the focus exclusively on the dominant lateral teeth [for reviews see^33–36,80^], except for one study on *Lepidochitona cinerea* determining the elemental composition of all tooth types^32^. Many of these analyses focused on the Fe biomineralization and the phase transformations during maturation [e.g.^23,29,81–89^]. Overall, in previous studies F, Na, Mg, Si, P, S, Cl, K, Ca, Fe, and Cu was detected in the dominant lateral teeth (= lateral teeth II) of Polyplacophora. For *Lepidochitona cinerea*, in our previous paper, we did not detect Cl, F, and Cu and in *Acanthochitona fascicularis* — no Si and Cu.

The following Fe proportions of mature dominant lateral teeth were previously determined in Polyplacophora: for *A. fascicularis* — 59.2% ^90^, 62%^85,91^ or few percent^92^ were detected. For *Plaxiphora *— 86.6%^90^, 17–27%^85^, or 26.7%^93^; for *Cryptochiton *— 51.8%^81^ or 69%^29^; for *Ischnochiton* — 62%^85^; for *Onithochiton* — 66%^85^ or 0.2%^93^; for *Cryptoplax —* ~ 90 weight % in the cap, ~ 30 weight % in the core, junction zone, and basis^83^; and for *Chiton* — 97%^94^ were detected. For mature *L. cinerea*, we previously^32^ found Fe proportions of maximal 32% (atomic ratio, atomic %) and for *A. fascicularis *— maximal 29% in the dominant lateral teeth.

For Polyplacophora, P was previously reported^81,83,85,93,95^ in form of iron phosphate^86,96,97^ or apatitic calcium phosphate^92,93,95,98–100^. F related to Ca^100,101^ was also previously reported for the dominant lateral teeth of chitons. In *Acanthopleura*^85,92^ and *Onithochiton*^85^, Ca was abundant to maximal ~ 30 atomic % and P to maximal ~ 20 atomic %. For *Lepidochitona cinerea,* we detected Ca in proportions of maximal 8% and P to 7% and for *Acanthochitona fascicularis* to maximal 6% and P to 9%.

Additionally, Si^83,85,98,99^ and Mg^85,93^ were previously detected in the dominant lateral teeth of chitons. S was also previously determined^83^. It is associated with the tanning of the organic matrix and with the appearance of proteins^82^. Additionally,^102^ detected Zi, K, F, S, Na, and Cl in radular segments of *Clavarizona*^90^, Ca, P, Mg, S, Na, Zi, K, Al, Cu, and Si in radulae of *Acanthopleura* and *Plaxiphora*, and^83^ Mg (with max ~ 5.5 weight %), K (with max ~ 1.0 weight %), Na (with max ~ 2.0 weight %), Si (with max ~ 1.0 weight %), Al (with max ~ 0.5 weight %), and S (with max ~ 0.8 weight %) in *Cryptoplax*. These elements, except for Zi and Cu, which were not found, occurred in smaller proportions (0–5%) in both *Lepidochitona cinerea* and *Acanthochitona fascicularis*. For the central, lateral I, and marginal teeth we detected less minerals than in the dominant lateral teeth in both species.

In Cephalopoda, only one study on the radular chemistry exists, to the best of our knowledge. In Octopoda^43^ targeted, but did not detect Si and Fe. We here determined Na, S, Cu, Si, P, Cl, Ca, and Mg in the radula of *Histioteuthis spec.* and *Loligo vulgaris* with proportions < 4%.

Within the Gastropoda, the Patellogastropoda received the most attention^41–43,90,103–115^. However, few studies focused on the overall radular composition, since most analyses, e.g. ashing and treatments with different acids or Raman spectroscopies, EDX, rather targeted the presence and crystalline shape of Fe and Si [e.g.^41,103,104,106–111,114^]. In fewer studies, other elements were of interest [e.g.^42,43,90,105,112,113^]. Overall, in Patellogastropoda, Na, Mg, Si, P, Cl, K, Ca, Fe, Cu, and S were previously found. For *Patella vulgata*, we here detected Na, Mg, Si, P, K, Ca, Fe, S, but no Cu and Cl. We additionally determined F. Similar to previous studies, we detected Si and Fe in high proportions in the dominant teeth (18–38%), whereas all other elements were present in smaller proportions only.

For the remaining gastropod taxa, only few analyses on the radular chemistry were conducted and usually the presence of elements, but not their proportions, could be determined. One of the earliest studies was done by^41^ depicting results from Bergh, who performed complex chemical analyses of ashing and dissolving radulae from the Caenogastropods *Charonia lampas* (detecting P, Ca, and Fe), *Lamellaria perspicua* (detecting no Si), and *Gibberulus gibberulus* (probably detecting none of these elements, this is not clear) in different acids. Additionally,^41^ presented his own results on the radulae of the Caenogastropod *Tonna galea* and the Heterobranch *Helix nemoralis* discovering P, Ca, and Fe in both by employing the same experiment. Sollas^42^ was the first, who studied the radular chemistry in an elevated quantity of taxa, and^43^ proceeded. Overall, their protocols are rather complex, involving analytical chemistry methods (ashing, staining, boiling, treating with acids, and using diffusion column) or physics (radula’s refractive index). Sollas^42^ determined rather the presence of elements and^43^ specifically tested the occurrence of Si and Fe. For the Caenogastropoda *Potamopyrgus antipodarum*, *Lacuna vincta*, *Murex branchialis*, and *Aporrhais pespelecani*, the Heterobranchia *Scaphander lignarius*, *Aplysia punctata*, and *Jorunna tomentosa,*^43^ determined no Si and no Fe. For the Heterobranchia (*Cornu aspersum*),^42^ determined Si and P*.* She detected Si in specimens collected during winter and phosphoric acid (P) in specimens collected during spring.^116^ performed EDX analyses on five specimen of *C. aspersum* detecting Ca in all specimen and Si in one, even though specimens were also inventoried in spring depicting the inconsistency of elements embedded. For the Vetigastropoda (*Haliotis tuberculata*),^42^ detected Si, Ca, and Fe.

The following species were studied by^42^ and^43^, but for many species their results are contradictory. In the Caenogastropoda *Littorina littorea*^42^ detected Mg, P, Ca, and Fe, whereas^43^ found no Ca and no Fe. *Nucella lapillus* and *Buccinum undatum* were also studied by^42^, but the results are not clear from the publication, and^43^ detected no Si and no Fe in both species. For the Vetigastropoda (*Emarginula fissure* and *Calliostoma zizyphinum*)^42^ detected P, Ca, and Fe, whereas^43^ determined the absence of Fe and Si. Then^117^ and^26,118^ were the first to close the existing gap in knowledge about the radular composition of Vetigastropoda. Gray^117^ detected Na, Mg, Si, Cl, Ca, and Fe (EDX), and^26,118^ Mg, Cl, Ca, and Fe (by EDX and inductively coupled plasma-optical emission spectrometry) in the limpet *Megathura crenulata*. Within Vetigastropoda, we detected Na, S, Cu, Si, P, Cl, Ca, and Mg; all of them in low proportions < 2%. Cu and S were not documented before, whereas Fe was detected in previous studies^117,118^. For the Neritimorpha, only one past study addresses the mineral content detecting S, Cl, K, Ca, Mg, Si, and Fe^119^. We additionally detected Na and P in *Vittina turrita*; all elements are abundant in very low proportions (< 4%). In the Caenogastropoda, we detected Fe, Mg, Ca, Cl, P, F, Si, Cu, S, Na, and K. Cu, F, Na, Si, S, and Cl were not determined before. In all species, proportions are < 6%. For the Heterobranchia, we detected more elements (Mg, Ca, Cl, P, F, Si, S, Na, K) than described in past publications^41–43,116^. Mg, Cl, F, S, Na, and K were not detected before. All elements are abundant at proportions < 15%.

Overall, the above data depicts that it is rather difficult to compare the percentages measured between studies, because in some weight percentages and in others atomic ratios were determined. Besides, methodology, sample preparation, and the analysed sample itself (whole radula or individual radular parts) differs. In addition, the presence and abundance of elements could potentially be influenced by the food available (e.g. plants containing or lacking Si) or by the chemistry of the saliva. In some taxa, specifically carnivorous gastropods, the saliva is acid [e.g.^120,121^], so potentially the contact of the outermost radular teeth with the saliva leads to reduced proportions. Both ideas await further research.

The generally accepted hypothesis on radular mineralization evolution states that all gastropods — besides Patellogastropoda, Neritimorpha, and Vetigastropoda — probably lack Fe in the radula [e.g.^122–124^]. However, Fe was detected previously in gastropod species [for *Tonna galea*, *Charonia lampas*, and *Helix nemoralis* see^41^, for *Littorina littorea* see^42^] and our own analyses determined it in *Reymondia horei* and *Littorina littorea*. Thus, this means that iron is not lacking, rather its proportions are reduced in these gastropod lineages (see Fig. 1).

Previous studies relate the radular length to the ingesta type. Herbivorous taxa were found to possess longer radulae than carnivorous ones^125^. Littorinid species, feeding on algae covering rocks, were found to possess longer radulae than species feeding from plant surface^126–130^. For *Patella* species, it was determined that the radular length increases with increasing usage and wear^131^ and, when algae are less abundant and the radula must thus be used more frequently to obtain the food necessary, its length increases^132^.

In general, we detected a similar pattern for the species studied here as the longest radulae with the highest quantity of tooth rows were found in species foraging on harder ingesta types (medium-to-solid, solid, medium) and the shortest ones in soft-substrate feeders. We, however, could not directly relate herbivory with longer radulae and carnivorous feeding with shorter ones. We additionally detected some relationship between radular length and proportions of elements (e.g. in *Patella vulgata*), so potentially more mineralized radulae are longer, because their maturation and mineralization requires more time and a longer contact to the overlain epithelia in the radular sac and mineralization zone. However, this does not seem to be the case for every species, as *Lepidochitona cinerea* and *Acanthochitona fascicularis* have relatively short heavily mineralized radulae. Thus, in these polyplacophoran species, the overlain epithelia can presumably incorporate more minerals at the same time or the radular replacement rate is faster in *P. vulgata* in contrast to the one in the Polyplacophora. Unfortunately, the radular replacement rate is known for few taxa: for Polyplacophorans (*Acanthopleura*, *Plaxiphora*, *Patelloida, Mopalia*), a rate of 0.36–0.80 rows per day was determined^90,133,134^ and for *P. vulgata,* a rate of 1.5 rows/day was described^135^. In Caenogastropoda, for *Lacuna* (Littorinidae), the rate of 3 rows/day^136^, for three *Littorina* species (Littorinidae) — 5–6 rows/day depending on the temperature^2,135^, and for *Pomatias elegans*— 5.02 rows/day^2^ was determined. For Heterobranchia, the rate of 2.9 rows/day in *Lymnaea stagnalis *^137^, 5.02 rows/day in *Agriolimax reticulatus*^2^, 3.6 rows/day in adult *Helix aspersa* (= *Cornu aspersum*) was detected^2^. For *Cepaea nemoralis,* the whole radula was found to be renewed within 30–35 days^138^. Thus, in general, a higher degree of mineralization is inversely related to the higher replacement rate (teeth that possess larger proportions of minerals are replaced slower). However, radular replacement seems to depend on many factors, such as water temperature, metabolic rates, or age of animals^135,136,139,140^. Further studies on these questions are required.

In general, we detected that radulae of species, foraging on the solid ingesta, possess heavy mineralized teeth and species feeding on the soft ingesta show the smallest proportions. In biological materials, heterogeneities can have their origin in geometry, chemistry, and/or structure [for a review see^141^]. In the dominant lateral teeth of chitons and limpets they have their origin in the distribution of the inorganic components and in the architecture of organic components^23,25,27–31^. We have previously correlated the hardness and elasticity values in *Lepidochitona cinerea* with the iron and the calcium proportions^32^, which was previously also described for limpet teeth^24,104,108,142^ and for other chitons^23,25,29,30^. For the paludomid gastropods, we previously measured elasticity modulus values ranging from 2 GPa at the tooth basis to 8 GPa in the cusp in solid substrate feeders, whereas soft substrate feeders possessed significantly softer teeth (4.6 GPa)^37,38,40^. In these species, we here detected inorganic elements in rather small proportions. We thus propose that specific cross-linking conditions of the chitin due to tanning^1^, fiber arrangement, and density^22,23,26,28,31,88,143–145^ rather cause the heterogeneities in mechanical properties. We previously also detected that the capability of wet teeth to rely on one another and to redistribute the mechanical stress increases the radula’s resistance to structural failure in paludomid gastropods^146,147^. This altogether probably enables the feeding on harder ingesta types. Whether these mechanisms are also applicable for the other molluscan species, await further investigations.

## Methods

### Specimen studied and dissection

Mollusks were obtained from various sources (see Table 1 for details): individuals of *Littorina littorea* and *Lepidochitona cinerea* were collected at the North Sea in summer 2019 and those of *Patella vulgata* in autumn 2020. The gastropods *Cornu aspersum*, *Rochia conus*, *Haliotis tuberculata*, *Vittina turrita*, *Faunus ater*, and *Anentome helena* were bought from online pet shops. All of them were shortly boiled and preserved in 70% ethanol. Individuals of *Aeolidia papillosa*, *Onchidoris bilamellata*, *Polycera quadrilineata*, and *Doris pseudoargus* were received from the Biologische Anstalt Helgoland in May 2021, kept in aquarium for 2 weeks in Hamburg, before the gastropods naturally died and then were preserved in 70% ethanol. Frozen specimens of *Loligo vulgaris* were bought from Fische Schmidt (store specialized on edible fish, Eppendorfer Baum 18, 20,249 Hamburg) for the dissection course of the Universität Hamburg, radulae were directly extracted from defrozen squids and preserved in 70% ethanol. Samples of *Lavigeria grandis*, *L. nassa*, *Paramelania damoni*, *Cleopatra johnstoni*, *Reymondia horei*, *Spekia zonata*, *Paludomus siamensis*, *Buccinum undatum*, *Acanthochitona fascicularis*, *Histioteuthis sp.* were extracted from already preserved (70% ethanol) specimens, some of them already inventoried in museum collections (Zoologisches Museum Hamburg, ZMH; Museum für Naturkunde Berlin, ZMB).

Species identification was reviewed by employing the relevant literature, the nomenclature and systematic position were checked using molluscabase.org. Not previously inventoried specimens were incorporated in the malacological collection of the ZMH, which is now part of the Leibniz-Institut für die Analyse des Biodiversitätswandels (LIB).

Overall, data from 72 adult specimens were analysed for this study. For each species, three adult specimens were selected, except for *Histioteuthis spec.* with two. We have chosen specimens of similar size per species, since the relationship between specimens’ length and radular size is puzzling. Some previous studies relate both parameters^148^ and others rather see a loose relationship or could not relate them for every species^125,149^. Additionally, the specimens chosen for each species were collected at the same time since seasonal dependencies in radular length were previously reported^18^. All data presented here is new, except for the elemental composition and radular morphology of *Lepidochitona cinerea*, which was taken from^32^. In this previous study, we analysed the ontogeny of the elemental composition and the mechanical parameters hardness and elasticity in three specimens of *L. cinerea*. For the present study, we included only the data from the working zone (the mature part) for the purpose of comparison between species.

Habitus images were either taken employing the Keyence Digital Microscope VHX-5000 (KEYENCE, Neu-Isenburg, Germany) or by using an iPad Pro (11 zoll; Apple Inc., Cupertino, USA) equipped with a 12-megapixel wide angle lens. Each specimen was dissected, the radula was carefully extracted by tweezers and then manually freed from surrounding tissue.

### Scanning electron microscopy (SEM)

For images of the whole radula or the radular working zone, radulae (three per species, except for *Histioteuthis spec.* with two) were cleaned in an ultrasonic bath for 2–20 s and afterwards arranged on scanning electron microscopy (SEM) sample holders (see Supplementary Figs. 1–24). All radulae were first documented with the Keyence Digital Microscope VHX-5000 or VHX-7000 (KEYENCE, Neu-Isenburg, Germany). Here the length and width of each radula were measured and the quantity of tooth rows counted. From the length and width, the radular area was calculated. Two radulae per species were then visualized uncoated employing the Tabletop Microscope TM 4000 Plus (Hitachi, Tokyo, Japan) for more detailed images and one radula per species was coated and documented with the Zeiss LEO 1525 (One Zeiss Drive, Thornwood, USA) to receive images with a very high resolution (except for images of *L. cinerea*, they were taken from^150^, and of *H. spec.*, as their radulae were documented uncoated and afterwards used for the EDX). Based on the morphology and arrangement of teeth, which were also categorized (e.g. central tooth, lateral tooth I, lateral tooth II, marginal tooth, inner teeth, outer teeth, etc.) according to their shape, size, and position on the membrane, radulae were assigned to different radular types (e.g. docogloss, isodont, rhipidogloss, etc.), if a suitable category could be determined from literature [e.g.^5,151–154^]. Then, the radulae, which were previously documented uncoated (two per species), were rewetted with 70% ethanol and loosened from the SEM sample holder and used for elemental analysis.

### Elemental analysis (EDX)

Wet radulae were arranged on glass object slides (Carl Roth, Karlsruhe, Germany) with double-sided adhesive tape. They were positioned along their longitudinal axis so that the outermost teeth of one side were directly attached to the slide. The adjacent and more inner teeth were located above, followed by the central teeth, the inner teeth from the other side, and finally, on top, outer teeth again. Each radula was then dried for three days under ambient temperature and afterwards surrounded with a small, metallic ring ensuring an almost parallel sample surface. Epoxy resin (RECKLI EPOXI WST, RECKLI GmbH, Herne, Germany) was filled into the metallic ring and left polymerizing at room temperature for three days. This specific epoxy was chosen, since it does not infiltrate the teeth. Object slide and tape were then removed and, to receive longitudinal sections of each tooth, the embedded radulae were polished until the outer teeth were on display (controlled by examining the samples in the light microscope) using sandpapers of different roughness. Then samples were smoothed with aluminium oxide polishing powder suspension of 0.3 μm grainsize (PRESI GmbH, Hagen, Germany) on a polishing machine (Minitech 233/333, PRESI GmbH, Hagen, Germany). After polishing, the samples were cleaned from the polishing powder by an ultrasonic bath lasting five minutes. Samples were then coated with platinum (5 nm layer) and the elemental compositions of specific areas of the embedded teeth were examined employing the SEM Zeiss LEO 1525 (One Zeiss Drive, Thornwood, New York, USA) equipped with an Octane Silicon Drift Detector (SDD) (micro analyses system TEAM, EDAX Inc., New Jersey, USA) always using an acceleration voltage of 20 keV and the same settings (e.g. lens opening, working distance, etc.). Before measuring a sample the detector was always calibrated using cupper. We performed elemental mappings for test purposes, but for elements that are present in rather lower proportions, this method is not sensitive enough. We thus focused on the elemental analysis of small areas (10–400 μm^2^, depending on the tooth) trying to analyse the largest possible area.

The elements H (hydrogen), C (carbon), N (nitrogen), O (oxygen), Pt (platinum), Al (aluminium), Ca (calcium), Na (sodium), Mg (magnesium), Si (silicon), P (phosphorus), S (sulphur), Cl (chlorine), K (potassium), F (fluorine), Cu (copper), and Fe (iron) were detected and their proportions measured. We used the data of atomic ratio (atomic %) for this study. These values were received with two positions after the decimal point, lower proportions were not detectable with this method and therefore they were given as 0.00. We did not analyse and discuss the following elements, as they are either the elemental basis of chitin (H, C, N, O), the coating (Pt), or the polishing powder (Al, O).

After analysing the outer teeth, each sample was again polished and smoothened until the next tooth type was on display; cleaning procedure and EDX analyses were again performed. These steps were repeated until all teeth were analysed. In this study, we present the results of the radular working zone, which is not covered by epithelia. Thus, all teeth are mature. Overall, we use data of 1448 analysed teeth from 49 specimens.

### Statistical analyses

All statistical analyses (mean, standard deviations) and visualizations with boxplots, pie charts, or trend lines were performed with JMP Pro, Version 14 (SAS Institute Inc., Cary, NC, 1989–2007). Correlation coefficients and PCA were also conducted in JMP.

### Composition- and biomineralization-types

With EDX analysis, the proportions of the individual elements, present in a defined area, can be documented, whereas the specific bonding and structure of molecules cannot be analysed. However, from the percentile occurrence, in comparison with past studies on the radular chemical composition involving, we propose that the elements, detected here, are potentially part of the following molecules or minerals. These were assigned to different composition- or biomineralization-types:

#### Category Type 1

Characterized by the presence of Fe. Potentially present in the form of magnetite as documented in polyplacophoran or goethite in limpets [e.g.^30,31,99,109,155–159^].

#### Category Type 2

Characterized by the presence of Mg and Ca. Elements are potentially involved in the protein packing, an increase in density of chitin fibres and in material stiffness as documented in limpet teeth^26^.

#### Category Type 3

Characterized by the presence of Ca, P, Cl and/or F. These elements (Ca:P:Cl/F) occur approximately in these ratios to one another: 5:3:1. They are potentially part of apatite, either fluorapatite, Ca_5_[F|(PO_4_)_3_], or chlorapatite, Ca_5_[Cl|(PO_4_)_3_] as previously described for radular teeth of polyplacophorans [e.g.^85,90,91,95,98,160–162^].

#### Category Type 4

Characterized by the presence of Si. Potentially present in the form of silica as documented in limpet teeth [e.g.^80,115^].

#### Category Type 5

Characterized by the presence of Cu.

#### Category OB (organic bonds)

The presence of Na, K, and S is often related to the protein bonding [e.g.^163,164^].

### Ingesta categories

The precise trophic preference of molluscan species is completely understudied. Some past approaches on various molluscan species determining the food preferences were based on the analysis of ingesta consumption, grazing activity, food choice experiments, stomach content, and faecal analyses [e.g.^10,165–177^]. These studies overall show, that very various food types can be intaken by one individual species. However, this cannot be generalized, since some taxa are less flexible with regard to the ingesta intaken [e.g. Nudibranchia, see^178^].

For this study, we defined ingesta categories that are rather broad, because (a) either the species’ food preference has only been described anecdotally and (b) even if the specific food type was known, its precise mechanical properties and its structural resistance to feeding is difficult to determine [see^179,180^] and that is why these properties remain unknown. We collated the data on ingesta from the literature, if available, and assigned it to the following ingesta categories (please, see Table 1 for food types per species and corresponding references):

*Soft* Algae from sand or mud, sea anemones. 

*Medium* Macro algae, soft parts of barnacles, encrusted Bryozoa, or Porifera.

*Solid* Algae/plants from rocks and/or cora ls.

*Soft to solid* Fish, crustaceans, and cephalopods; algae from rocks and sand; gastropods, fish eggs, shrimps, and carrion; Polychaeta, fish eggs, bivalves, carrion, etc. 

*Medium to solid* Algae, fleshy macro algae, also from rocks.

### Systematics 

The cladogram, depicting the systematic position of the species studied, was created by combining data from published phylogenies^12,181–189^ with data on the latest systematic position of the species from *molluscabase.org*.

### Human or animals rights

For this article no research was conducted on live vertebrates and/or higher invertebrates.

## Supplementary Information

Supplementary Information.

## Data Availability

All data is provided in the supplementary material.
