# Correction: Elemental analyses reveal distinct mineralization patterns in radular teeth of various molluscan taxa

**DOI:** 10.1038/s41598-025-20246-9

**Published:** 2025-10-07

**Authors:** Wencke Krings, Jan-Ole Brütt, Stanislav N. Gorb

**Affiliations:** 1 Department of Behavioral Biology, Institute of Cell and Systems Biology of Animals, Universität Hamburg, Martin-Luther-King-Platz 3, 20146 Hamburg, Germany; 2 Department of Mammalogy and Palaeoanthropology, Leibniz Institute for the Analysis of Biodiversity Change, Martin-Luther-King-Platz 3, 20146 Hamburg, Germany; 3 Department of Functional Morphology and Biomechanics, Zoological Institute, Christian-Albrechts-Universität Zu Kiel, Am Botanischen Garten 9, 24118 Kiel, Germany

Correction to: *Scientific Reports* 10.1038/s41598-022-11026-w, published online 07 May 2022

The original version of this Article contained an error in Figure 1, where “Si” and “Fe” in the group “Patellogastropoda” were swapped. The original Figure 1 and accompanying legend appear below.

**Fig. 1:**
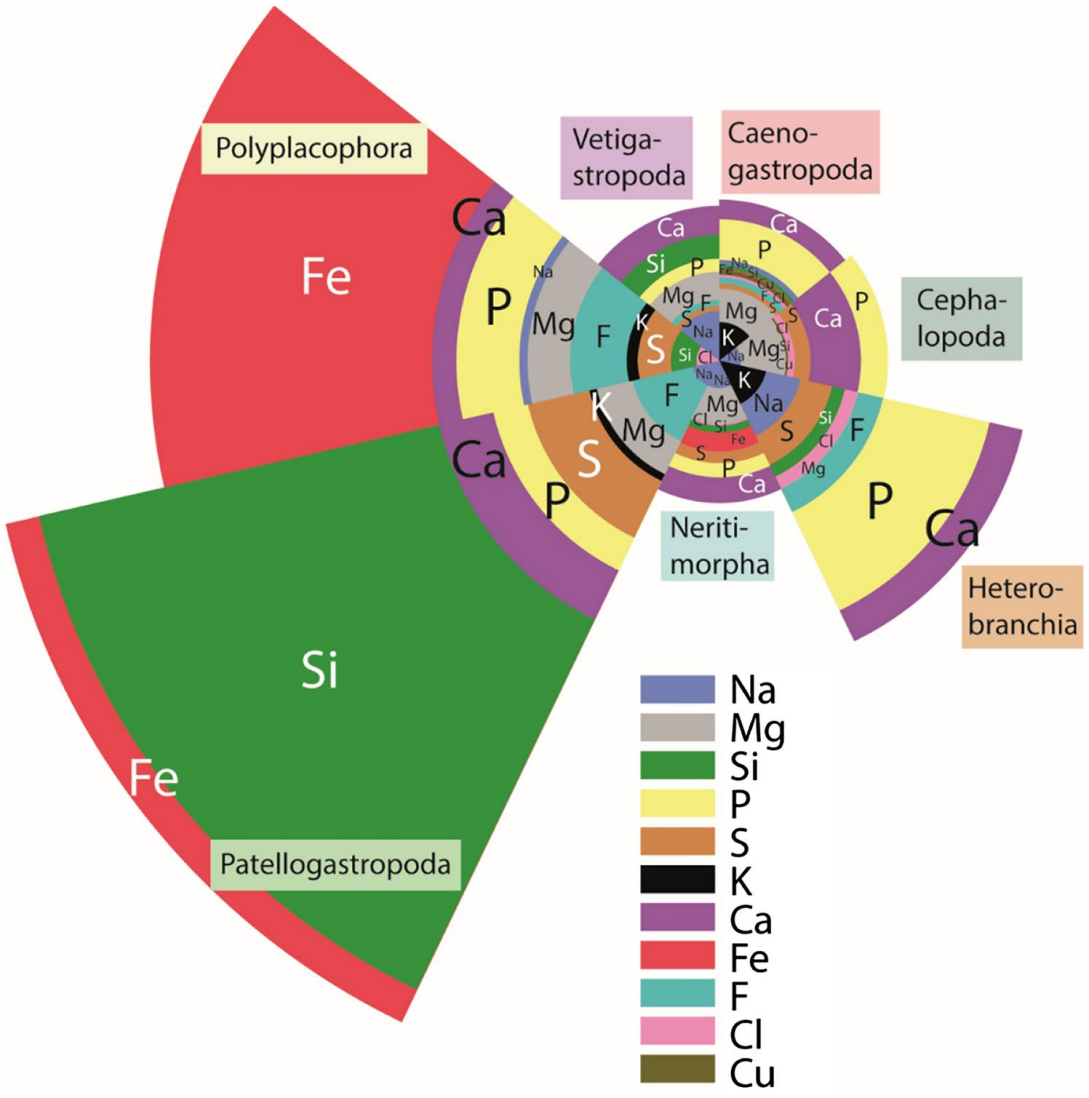
Elemental proportions of the species’ radular teeth, summarized for taxa (Patellogastropoda, Polyplacophora, Vetigastropoda, Caenogastropoda, Cephalopoda, Heterobranchia, Neritimorpha) to ease comparison in the radular mineral content.

The original Article has been corrected.

