# Supplementary material for: Elemental analyses reveal distinct mineralization patterns in radular teeth of various molluscan taxa
Source: Sci Rep. 2022 May 7;12:7499. doi: 10.1038/s41598-022-11026-w (PMC9079087; doi:10.1038/s41598-022-11026-w)
Supplement: Supplementary file 1 — Supplementary Information. [file 41598_2022_11026_MOESM1_ESM.pdf]

Krings et al., 2022

Elemental analyses reveal distinct mineralization patterns in radular teeth of various Molluscan taxa

Wencke Krings[1,2,3*], Jan-Ole Brütt[1,2], Stanislav N. Gorb[3]

[1] Department of Behavioral Biology, Institute of Cell and Systems Biology of Animals, Universität Hamburg, Martin-Luther-King-Platz 3, 20146 Hamburg, Germany
[2] Department of Mammalogy and Palaeoanthropology, Leibniz Institute for the Analysis of Biodiversity Change, Martin-Luther-King-Platz 3, 20146 Hamburg, Germany
[3] Department of Functional Morphology and Biomechanics, Zoological Institute, Christian-Albrechts-Universität zu Kiel, Am Botanischen Garten 9, 24118 Kiel, Germany
*corresponding author: wencke.krings@uni-hamburg.de

Supplementary materials

Additional references for Supplementary:

190. Krings, W., Karabacak, H. & Gorb, S. N. From the knitting shop: the first physical and dynamic model of the taenioglossan radula (Mollusca: Gastropoda) aids in unravelling functional principles of the radular morphology. J. R. Soc. Interface 18(182), 20210377 (2121).

191. Krings, W., Marcé-Nogué, J., Karabacak, H., Glaubrecht, M. & Gorb, S. N. Finite element analysis of individual taenioglossan radular teeth (Mollusca). Acta Biomater. 115, 317-332 (2020).

192. Krings, W. & Gorb, S. N. Radula packing and storage facilitated by tooth morphology in selected taenioglossan Gastropoda. J. Molluscan Stud. 87(2), eyab007 (2021).

193. Lee, A. P., Brooker, L. R., Bronswijk, W. van, Macey, D. J. & Webb, J. Contribution of Raman spectroscopy to identification of biominerals present in teeth of *Acanthopleura rehderi*, *Acanthopleura curtisiana*, and *Onithochiton quercinus*. Biopolymers 72, 299-301 (2003).

194. van der Wal et al., 1989 van der Wal, P., Videler, J. J., Havinga, P., Pel, R. Architecture and chemical composition of the magnetite-bearing layer in the radula teeth of *Chiton olivaceus* (Polyplacophora). In: Crick, R. E. (eds) Origin, Evolution, and Modern Aspects of Biomineralization in Plants and Animals. Springer, Boston, MA (1989).

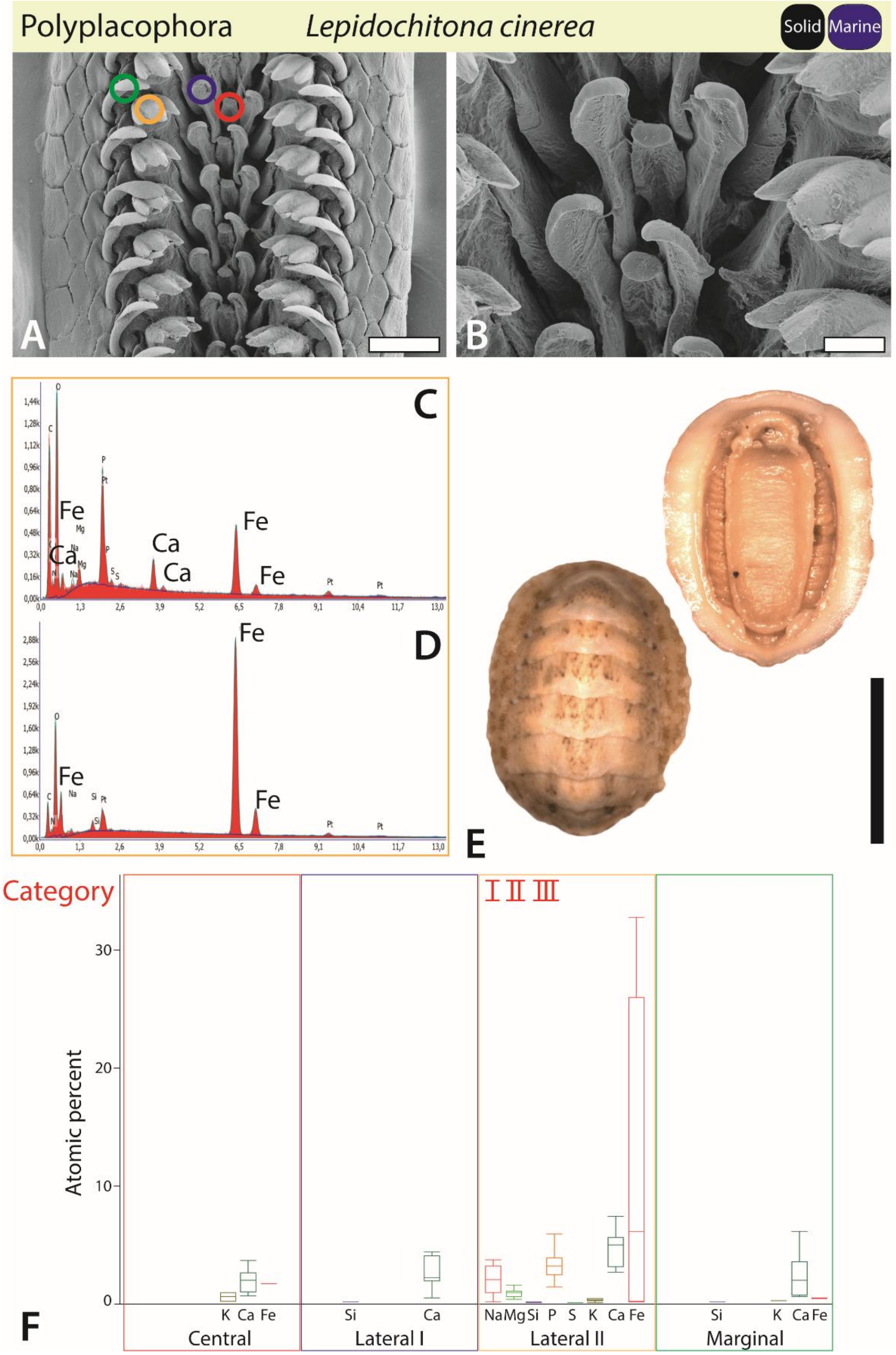

Supplementary Figure 1. ***Lepidochitona cinerea***: A-B. SEM images of the working zone of one representative radula (adapted from 32). The circles indicate the area of the EDX analysis: green, marginal; yellow, lateral II; blue, lateral I; red, central teeth. C-D. Representative EDX spectra of the lateral tooth II. E. Habitus from one representative specimen in dorsal and ventral views. F. Results from EDX analyses: elemental proportions, given in atomic percent, for central, lateral I, lateral II, and marginal teeth. Proposed biomineralization categories for each tooth type are written in red. Scale bars: A, 100 µm; B, 30 µm; E, 5 mm.

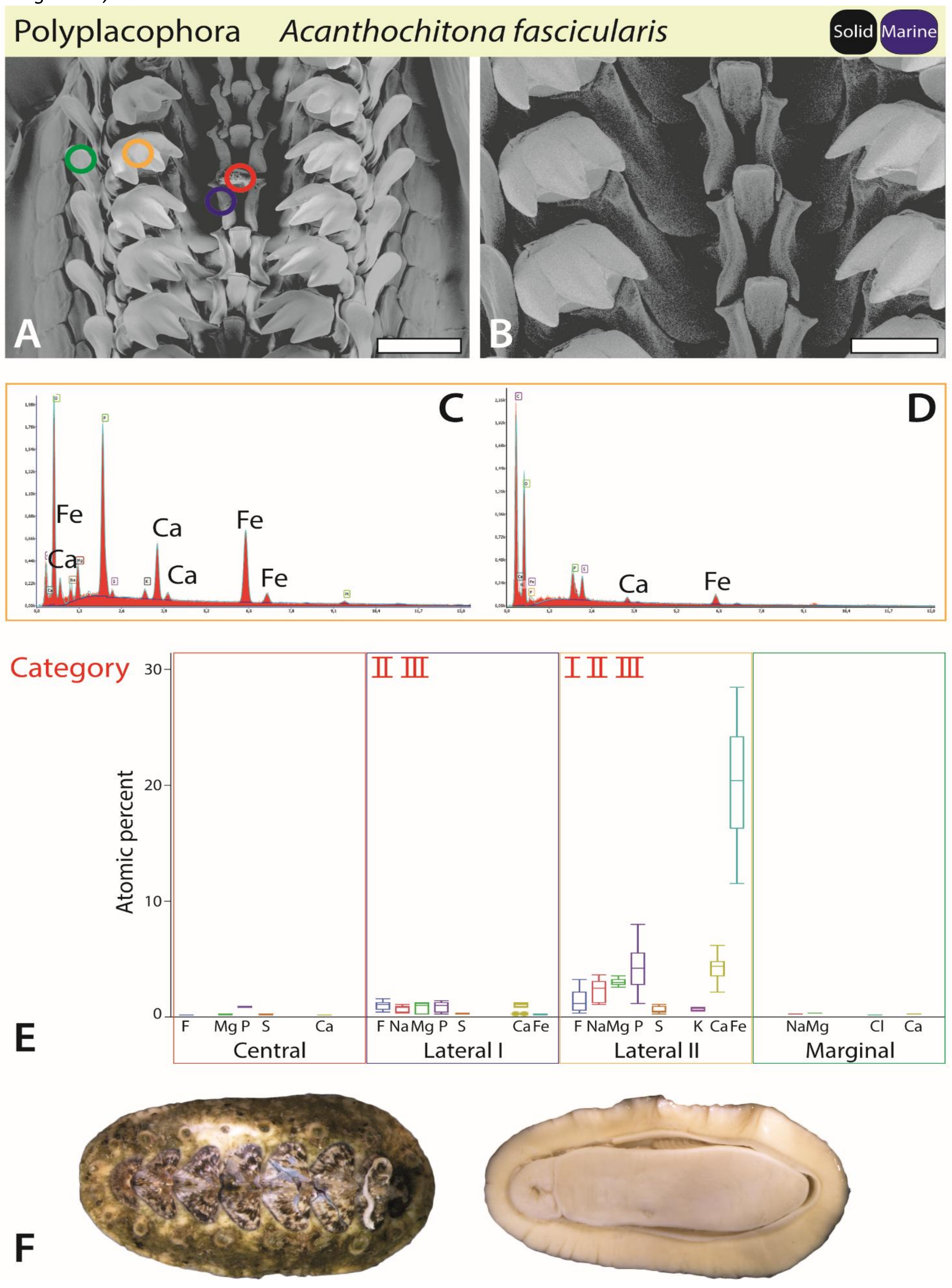

Supplementary Figure 2. ***Acanthochitona fascicularis***: A-B. SEM images of the working zone of one representative radula. The circles indicate the area of the EDX analysis: green, marginal; yellow, lateral II; blue, lateral I; red, central teeth. C-D. Representative EDX spectra of the lateral tooth II. E. Results from EDX analyses: elemental proportions, given in atomic percent, for central, lateral I, lateral II, and marginal teeth. F. Habitus from one representative specimen in dorsal and ventral views. Proposed biomineralization categories for each tooth type are written in red. Scale bars: A, 200 µm; B, 100 µm.

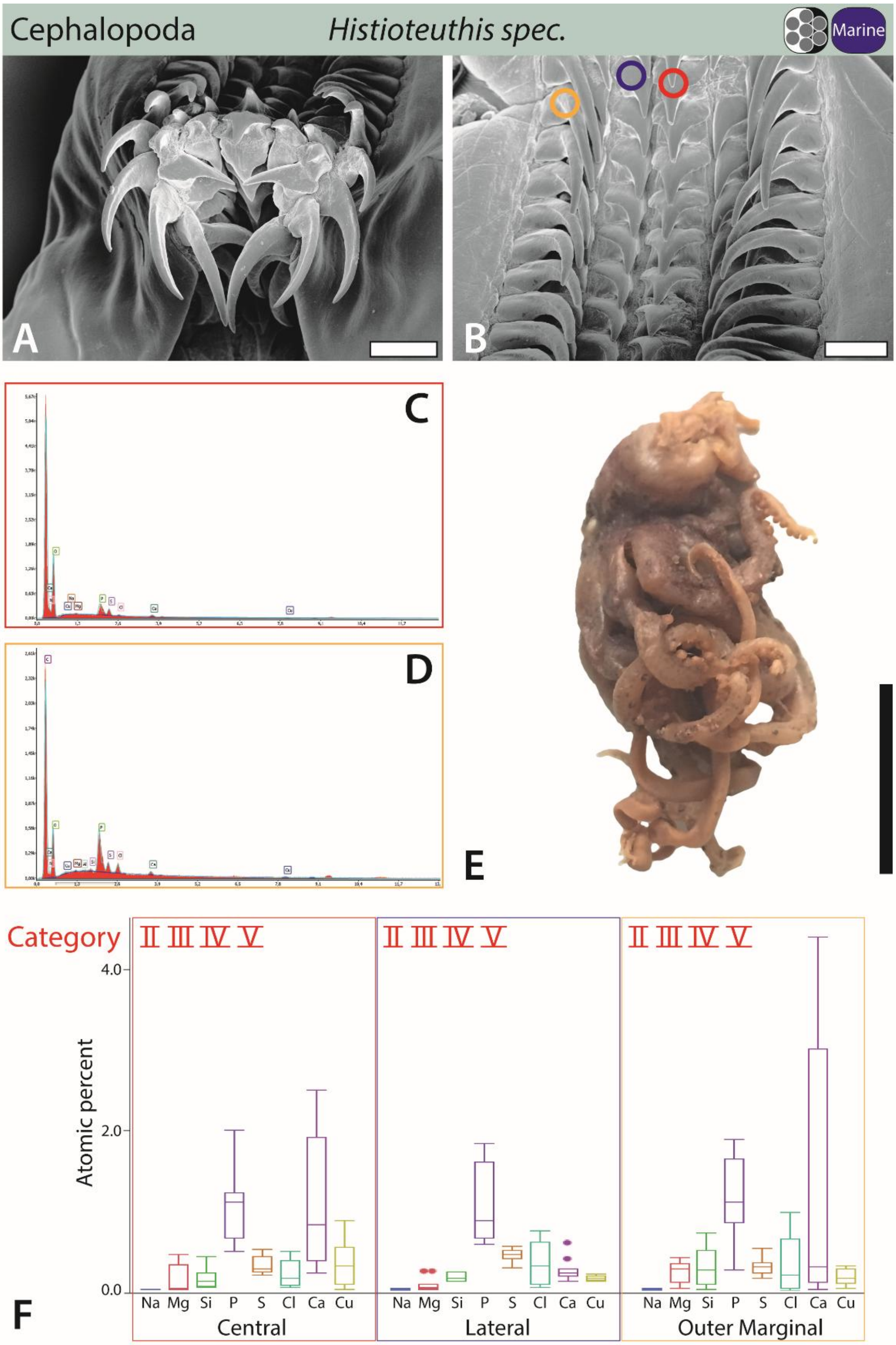

Supplementary Figure 3. *Histioteuthis spec.*: A-B. SEM images of the working zone of one representative radula. The circles indicate the area of the EDX analysis: yellow, outer marginal; blue, lateral; red, central teeth. C-D. Representative EDX spectra of central (C) and outer marginal (D) teeth. E. Habitus of one representative specimen. F. Results from EDX analyses: elemental proportions, given in atomic percent, for central, lateral I, and outer marginal teeth. Proposed biomineralization categories for each tooth type are written in red. Scale bars: A, 100 µm; B, 60 µm; E, 2 cm.

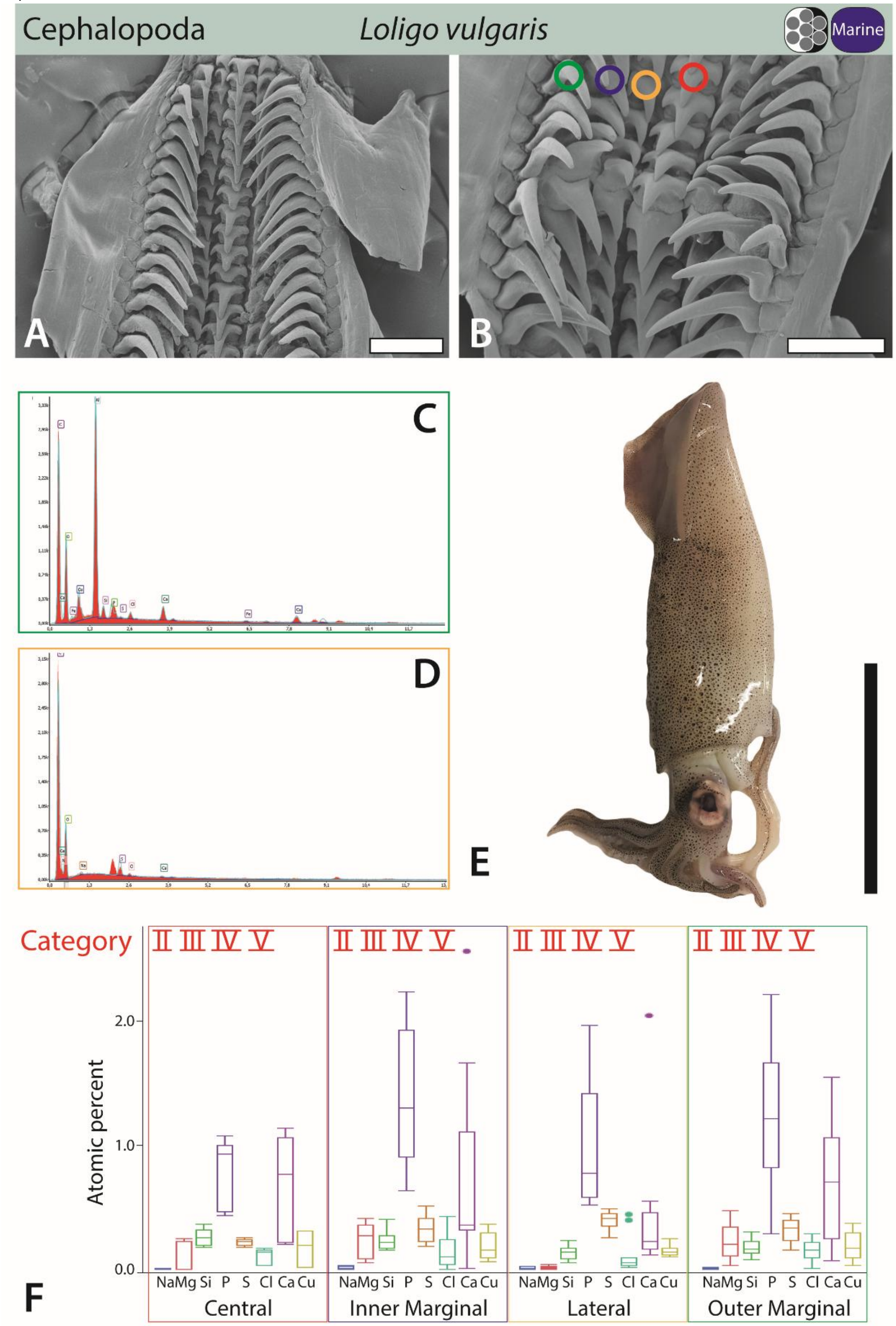

Supplementary Figure 4. ***Loligo vulgaris***: A-B. SEM images of the working zone of one representative radula. The circles indicate the area of the EDX analysis: green, outer marginal; blue, inner marginal; yellow, lateral; red, central teeth. C-D. Representative EDX spectra of outer marginal (C) and lateral (D) teeth. E. Habitus of one representative specimen. F. Results from EDX analyses: elemental proportions, given in atomic percent, for central, inner marginal, lateral, and outer marginal teeth. Proposed biomineralization categories for each tooth type are written in red. Scale bars: A, 300 µm; B, 400 µm; E, 10 cm.

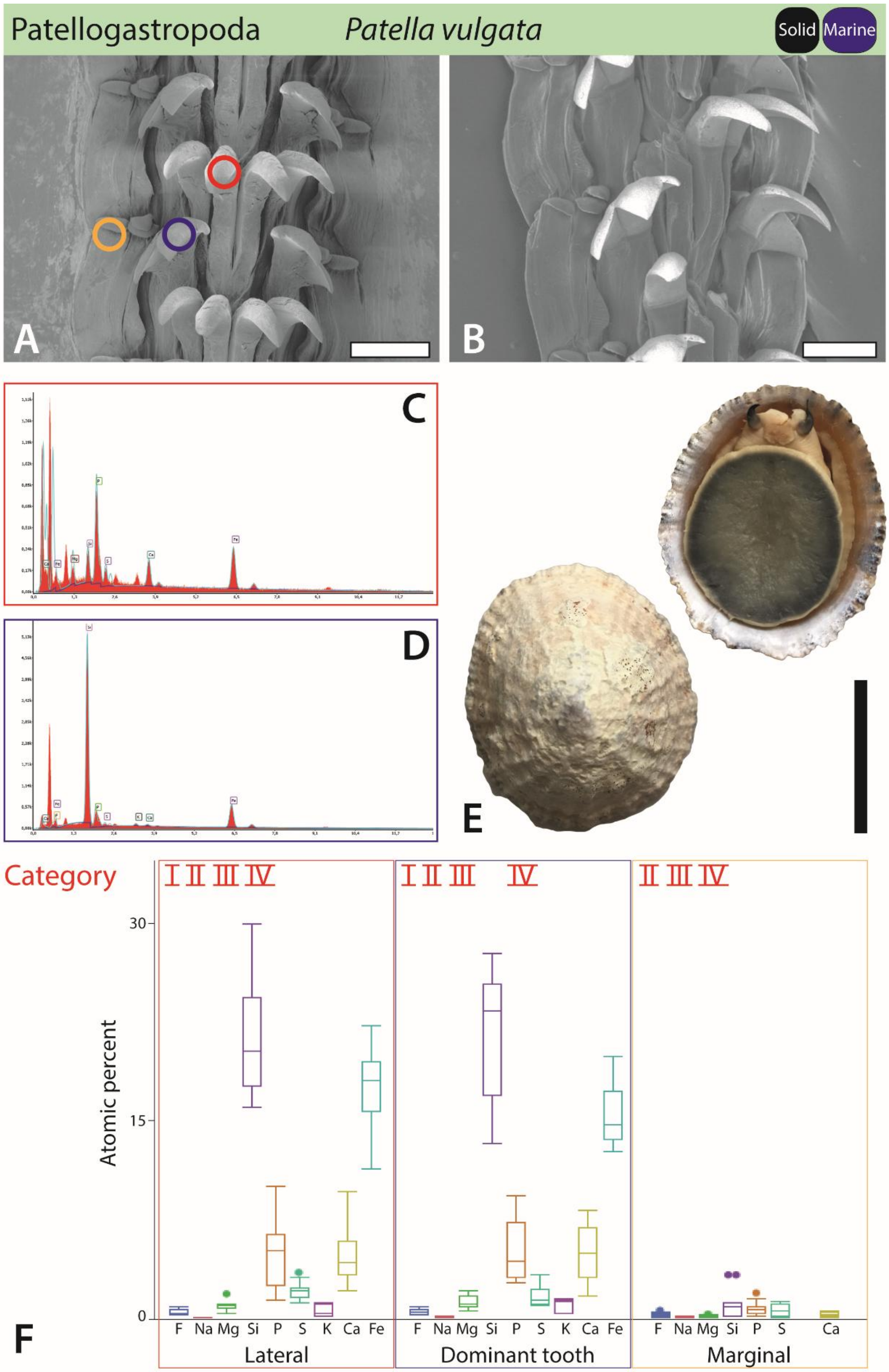

Supplementary Figure 5. ***Patella vulgata***: A-B. SEM images of the working zone of one representative radula. The circles indicate the area of EDX analysis: yellow, marginal; blue, lateral; red, central teeth. C-D. Representative EDX spectra of central (C) and lateral I (D) teeth. E. Habitus from one representative specimen in dorsal and ventral views. F. Results from EDX analyses: elemental proportions, given in atomic percent, for central, lateral, and marginal teeth. Proposed biomineralization categories for each tooth type are written in red. Scale bars: A, 200 µm; B, 150 µm; E, 2.5 cm.

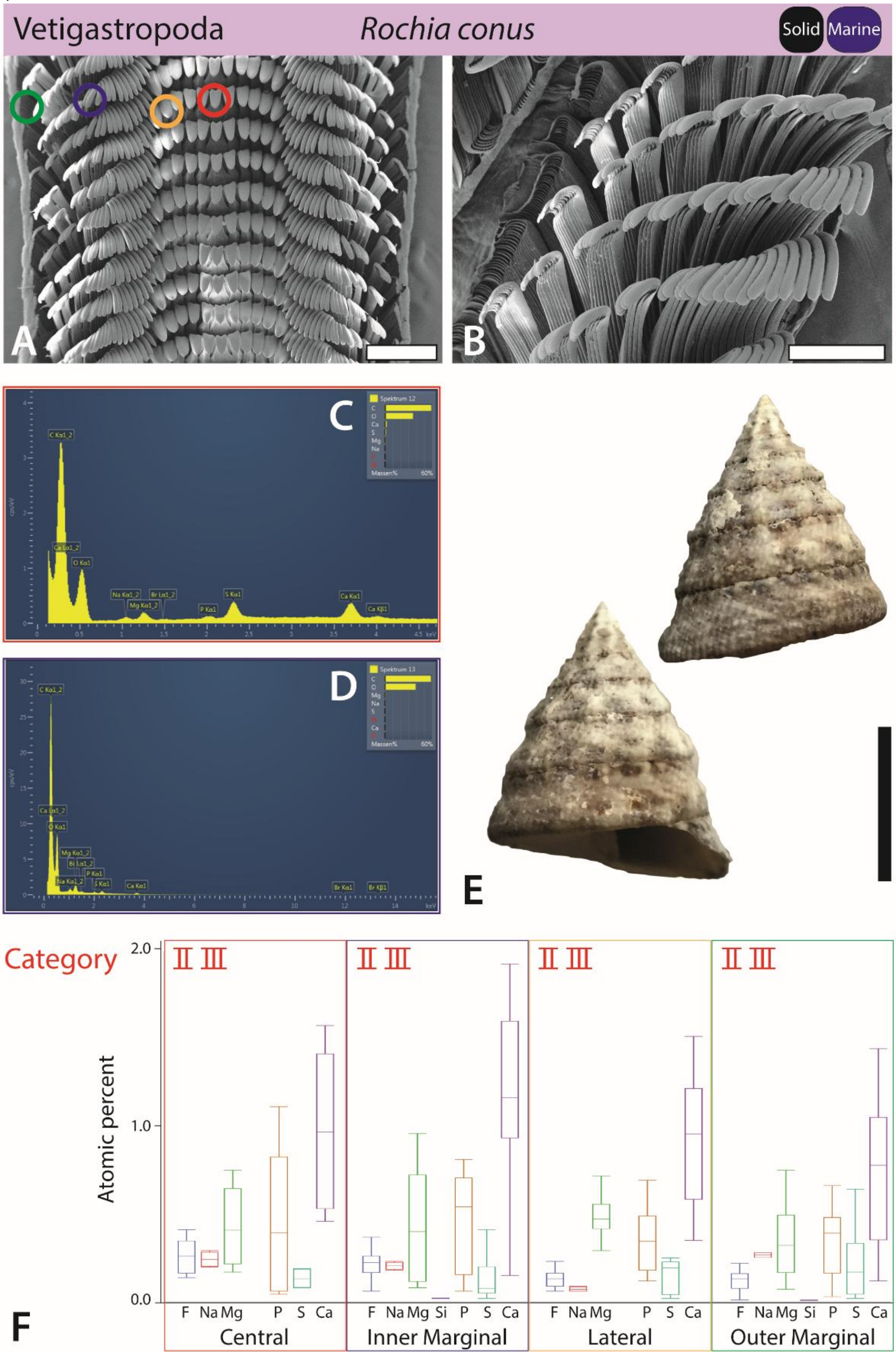

Supplementary Figure 6. ***Rochia conus***: A-B. SEM images of the working zone of one representative radula. The circles indicate the area of the EDX analysis: green, outer marginal; blue, inner marginal; yellow, lateral; red, central teeth. C-D. Representative EDX spectra of central (C) and inner marginal (D) teeth. E. Habitus from one representative specimen in dorsal and ventral views. F. Results from EDX analyses: elemental proportions, given in atomic percent, for central, inner marginal, lateral, and outer marginal teeth. Proposed biomineralization categories for each tooth type are written in red. Scale bars: A, 600 µm; B, 300 µm; E, 3.5 cm.

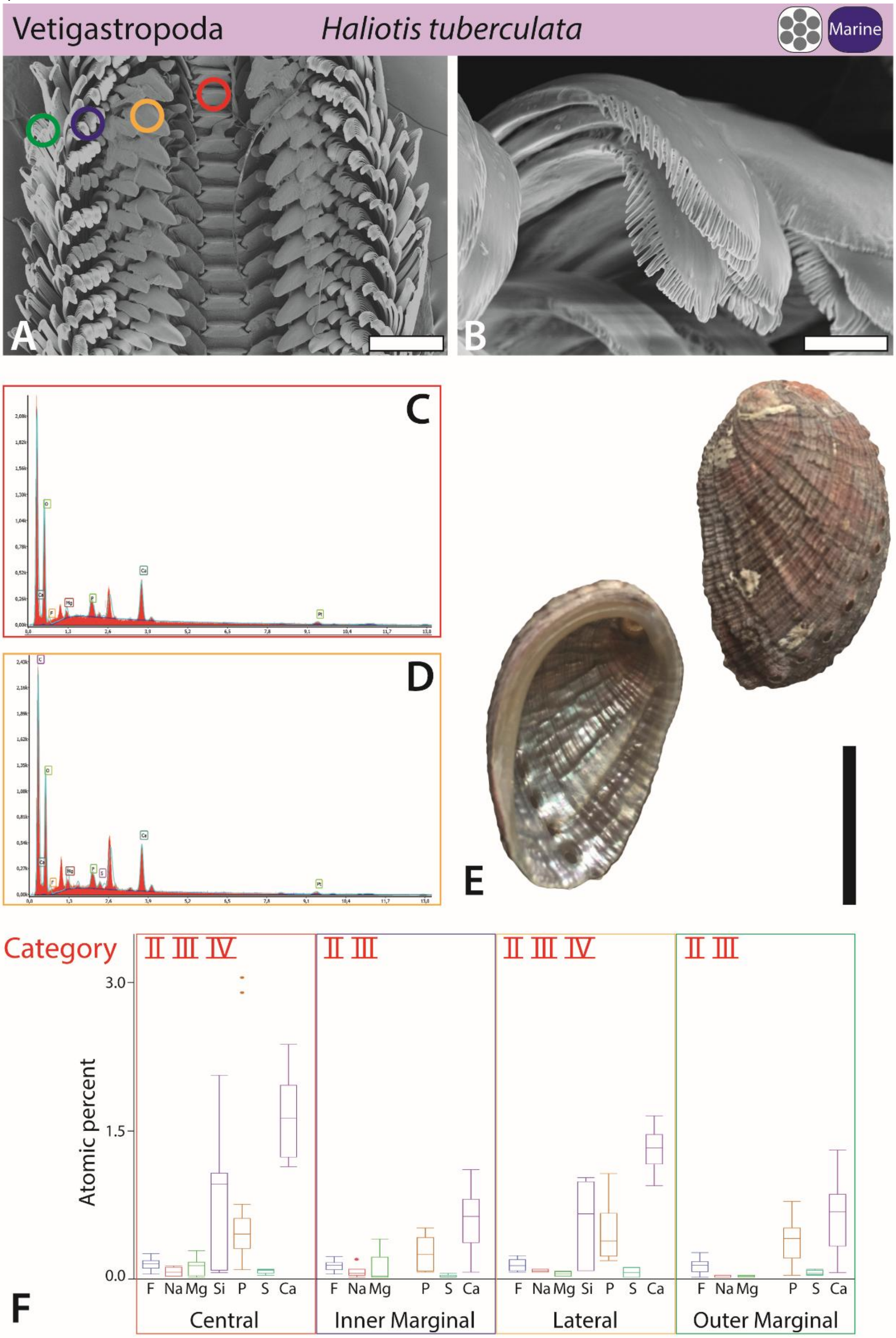

Supplementary Figure 7. *Haliotis tuberculata*: A-B. SEM images of the working zone of one representative radula. The circles indicate the area of the EDX analysis: green, outer marginal; blue, inner marginal; yellow, lateral; red, central teeth. C-D. Representative EDX spectra of central (C) and lateral (D) teeth. E. Habitus from one representative specimen in dorsal and ventral views. F. Results from EDX analyses: elemental proportions, given in atomic percent, for central, inner marginal, lateral, and outer marginal teeth. Proposed biomineralization categories for each tooth type are written in red. Scale bars: A, 400 µm; B, 10 µm; E, 5.5 cm.

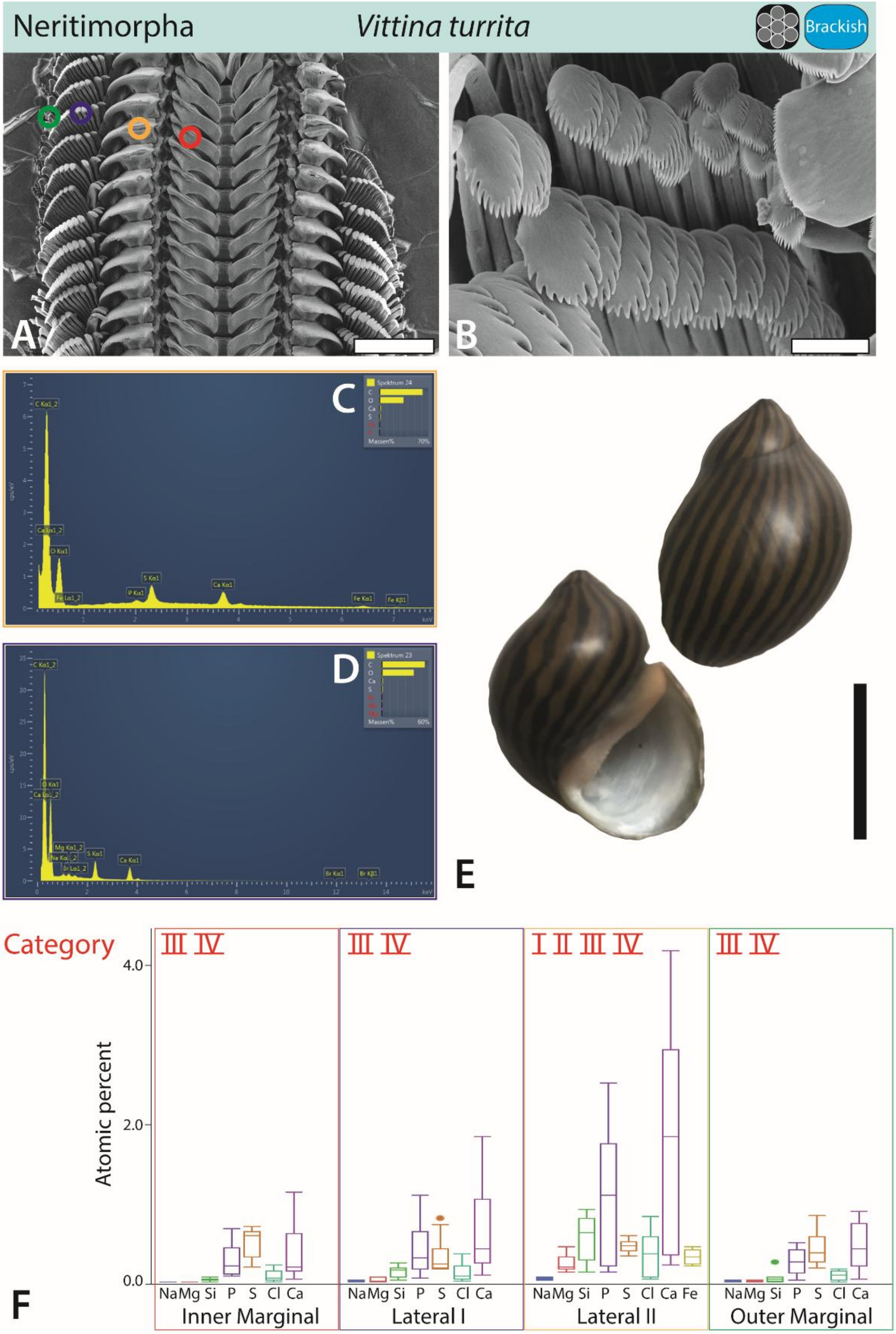

Supplementary Figure 8. ***Vittina turrita***: A-B. SEM images of the working zone of one representative radula. The circles indicate the area of the EDX analysis: green, outer marginal; blue, inner marginal; yellow, lateral II; red, lateral I teeth. C-D. Representative EDX spectra of central (C) and lateral (D) teeth. E. Habitus from one representative specimen in dorsal and ventral views. F. Results from EDX analyses: elemental proportions, given in atomic percent, for inner marginal, lateral I, lateral II (=dominant lateral teeth), and outer marginal teeth. Proposed biomineralization categories for each tooth type are written in red. Scale bars: A, 300 µm; B, 10 µm; E, 1.5 cm.

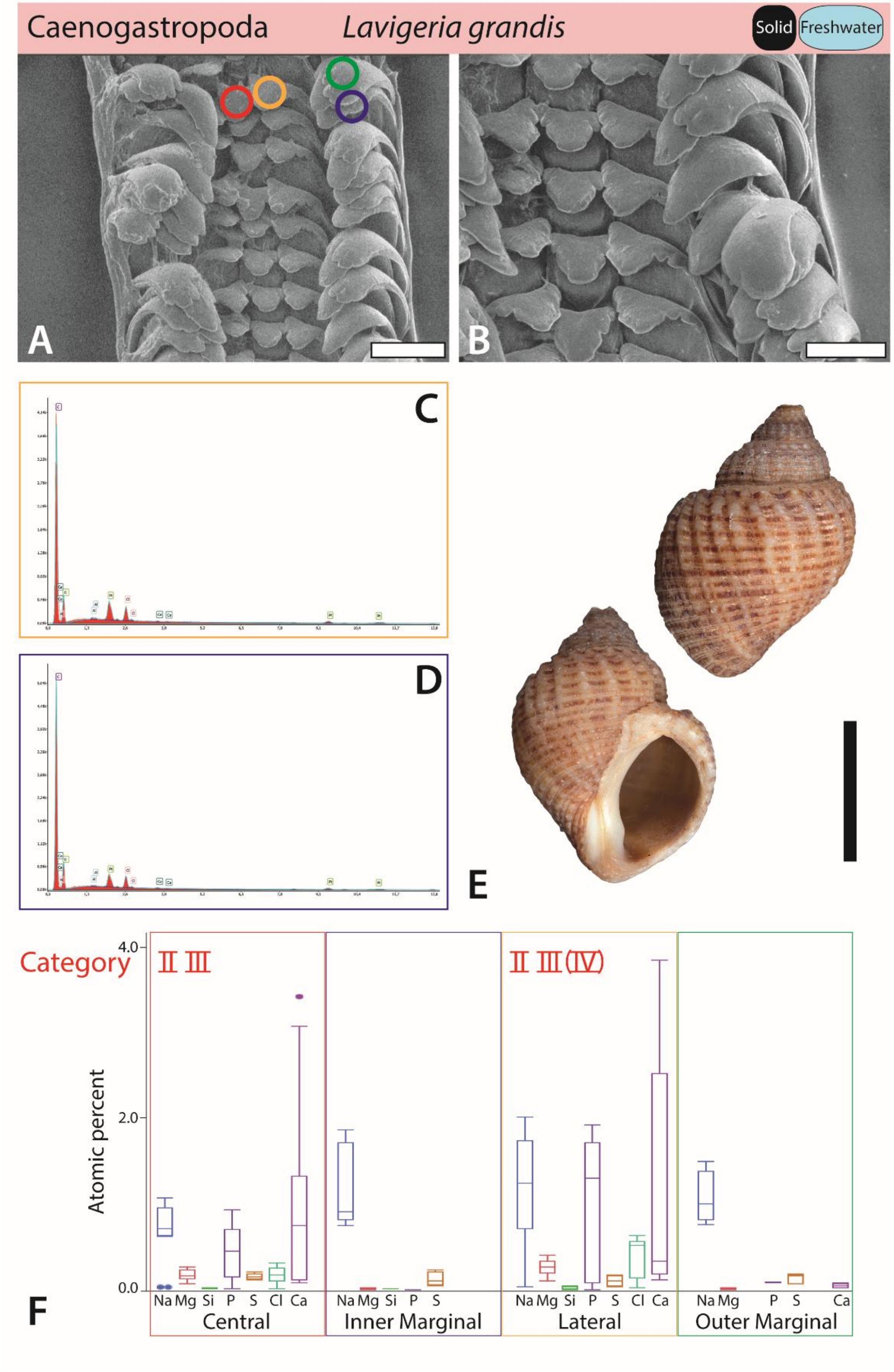

Supplementary Figure 9. ***Lavigeria grandis***: A-B. SEM images of the working zone of one representative radula. The circles indicate the area of the EDX analysis: green, outer marginal; blue, inner marginal; yellow, lateral; red, central teeth. C-D. Representative EDX spectra of lateral (C) and inner marginal (D) teeth. E. Habitus from one representative specimen (ZMB 220.121_3) in dorsal and ventral views. F. Results from EDX analyses: elemental proportions, given in atomic percent, for central, inner marginal, lateral, and outer marginal teeth. Proposed biomineralization categories for each tooth type are written in red. Scale bars: A, 150 µm; B, 100 µm; E, 1 cm.

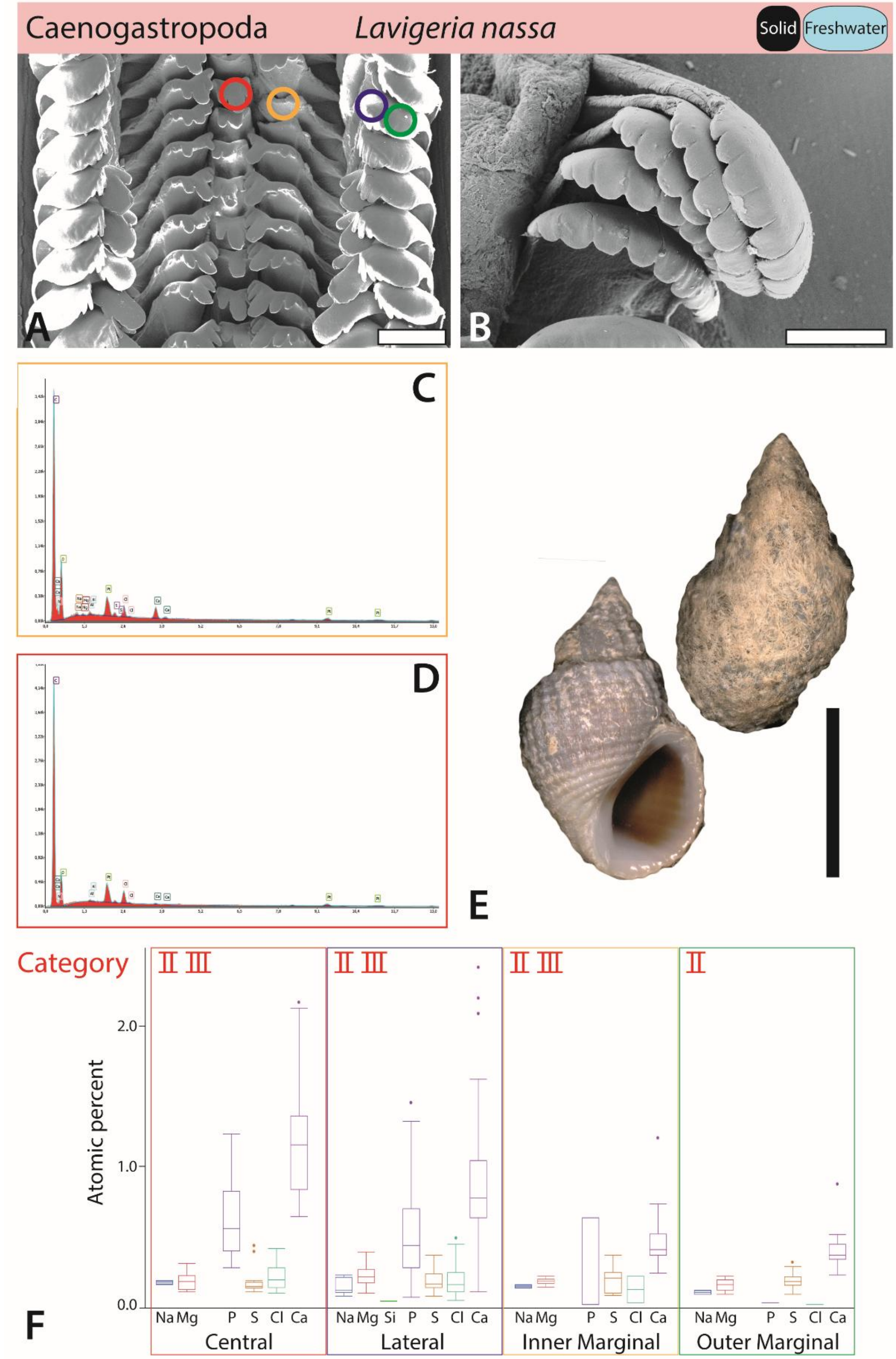

Supplementary Figure 10. ***Lavigeria nassa***: A-B. SEM images of the working zone of one representative radula. The circles indicate the area of the EDX analysis: green, outer marginal; blue, inner marginal; yellow, lateral; red, central teeth. C-D. Representative EDX spectra of lateral (C) and central (D) teeth. E. Habitus from one representative specimen in dorsal and ventral views. F. Results from EDX analyses: elemental proportions, given in atomic percent, for central, lateral, inner marginal, and outer marginal teeth. Proposed biomineralization categories for each tooth type are written in red. Scale bars: A, 80 µm; B, 40 µm; E, 1 cm.

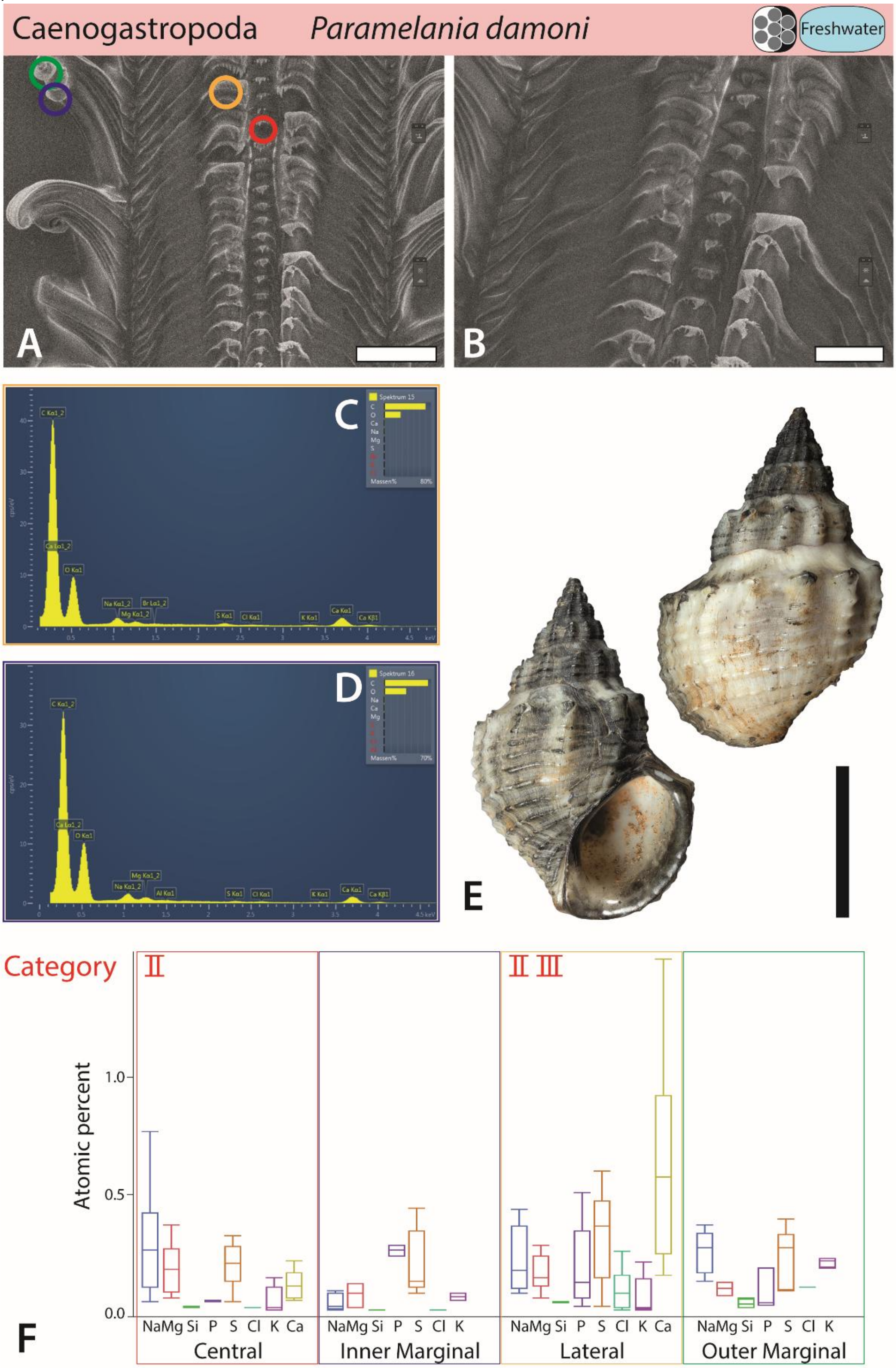

Supplementary Figure 11. *Paramelania damoni*: A-B. SEM images of the working zone of one representative radula. The circles indicate the area of the EDX analysis: green, outer marginal; blue, inner marginal; yellow, lateral; red, central teeth. C-D. Representative EDX spectra of lateral (C) and inner marginal (D) teeth. E. Habitus from one representative specimen (ZMB 92361_1) in dorsal and ventral views. F. Results from EDX analyses: elemental proportions, given in atomic percent, for central, inner marginal, lateral, and outer marginal teeth. Proposed biomineralization categories for each tooth type are written in red. Scale bars: A, 100 µm; B, 50 µm; E, 1 cm.

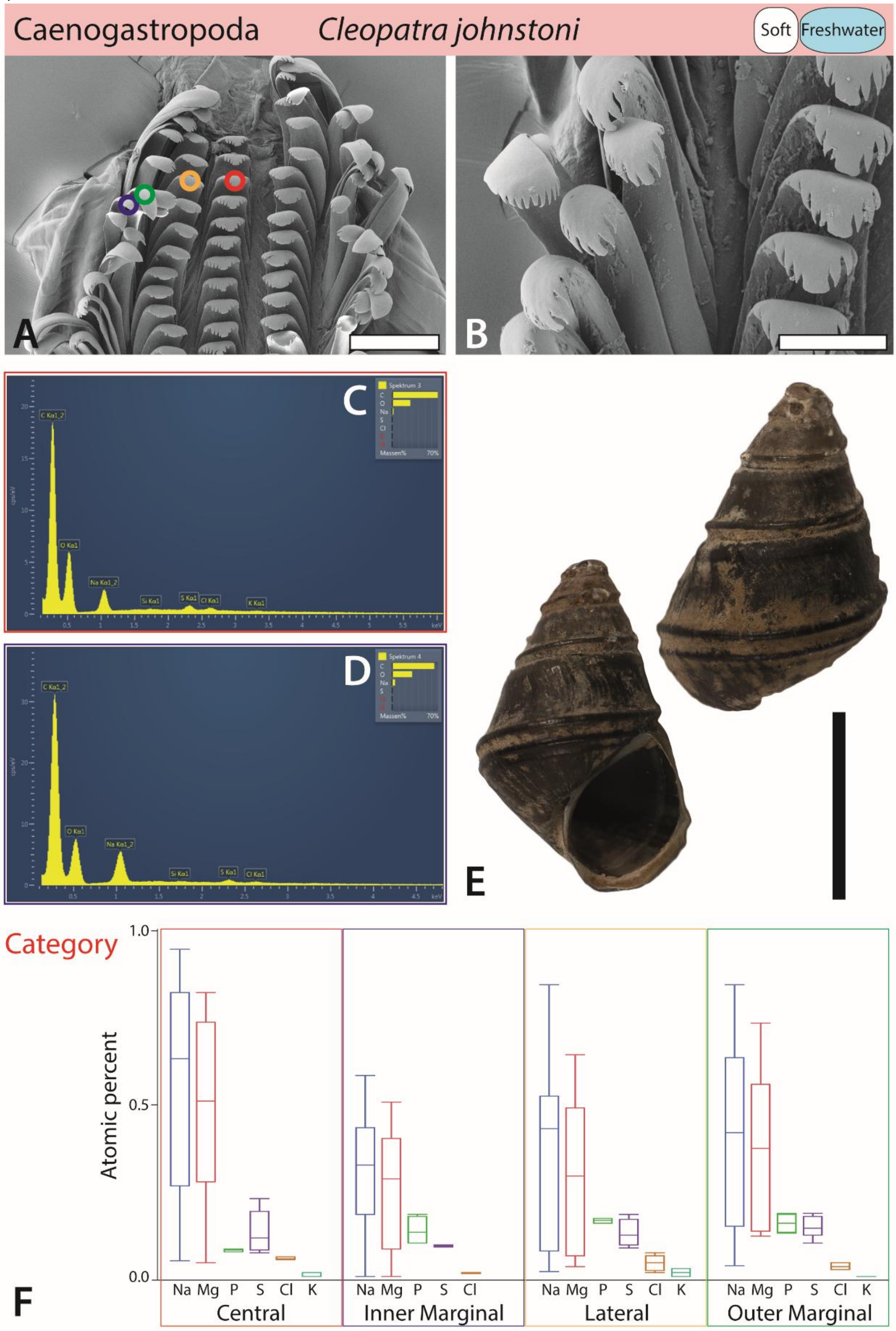

Supplementary Figure 12. ***Cleopatra johnstoni***: A-B. SEM images of the working zone of one representative radula. The circles indicate the area of EDX analysis: green, outer marginal; blue, inner marginal; yellow, lateral; red, central teeth. C-D. Representative EDX spectra of central (C) and inner marginal (D) teeth. E. Habitus from one representative specimen in dorsal and ventral views. F. Results from EDX analyses: elemental proportions, given in atomic percent, for central, inner marginal, lateral, and outer marginal teeth. Proposed biomineralization category for each tooth type are written in red (none for this species). Scale bars: A, 200 µm; B, 80 µm; E, 1 cm.

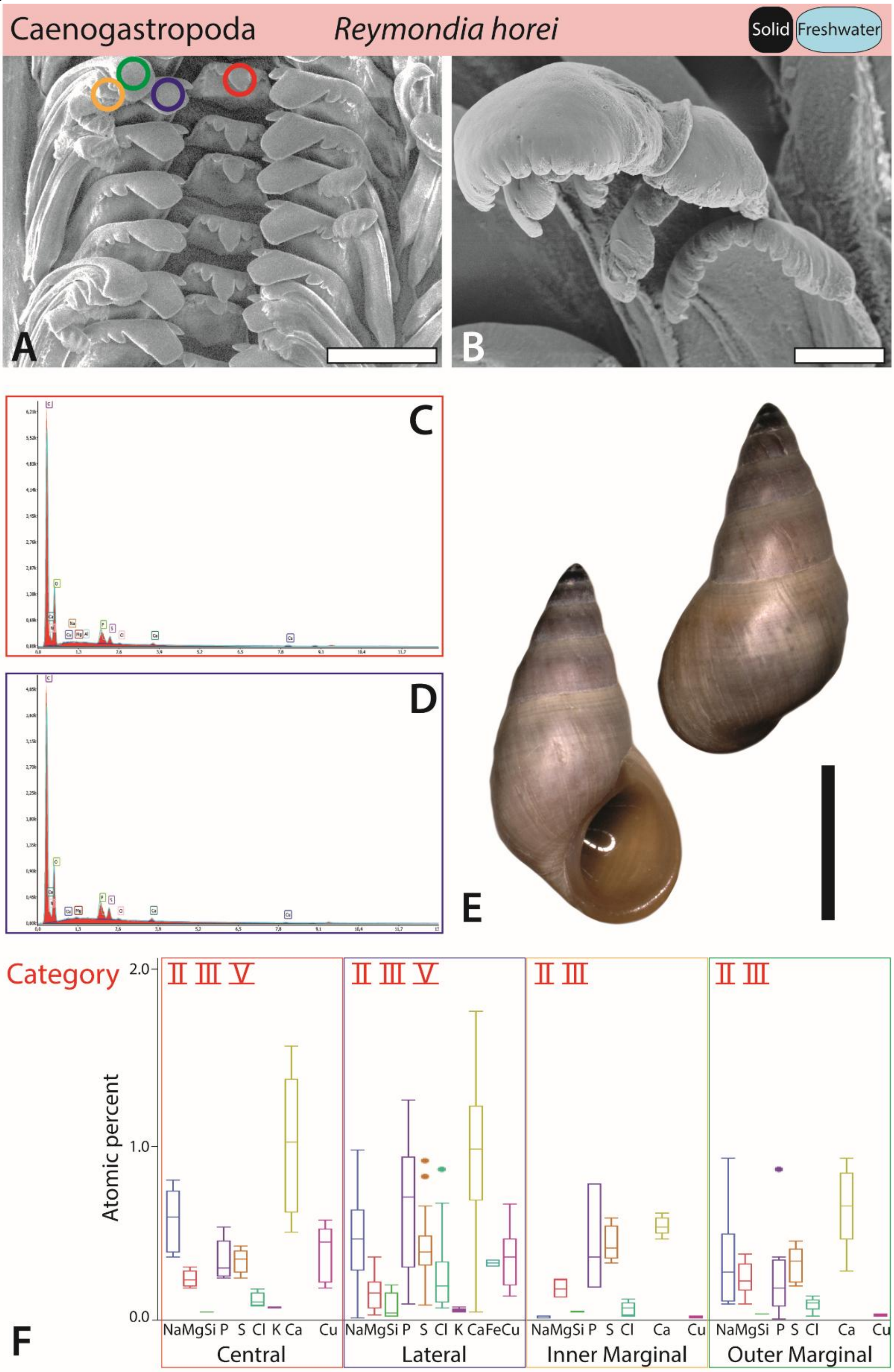

Supplementary Figure 13. ***Reymondia horei***: A-B. SEM images of the working zone of one representative radula. The circles indicate the area of the EDX analysis: green, outer marginal; yellow, inner marginal; blue, lateral; red, central teeth. C-D. Representative EDX spectra of central (C) and lateral (D) teeth. E. Habitus from one representative specimen in dorsal and ventral views. F. Results from EDX analyses: elemental proportions, given in atomic percent, for central, lateral, inner marginal, and outer marginal teeth. Proposed biomineralization categories for each tooth type are written in red. Scale bars: A, 100 µm; B, 20 µm; E, 5 mm.

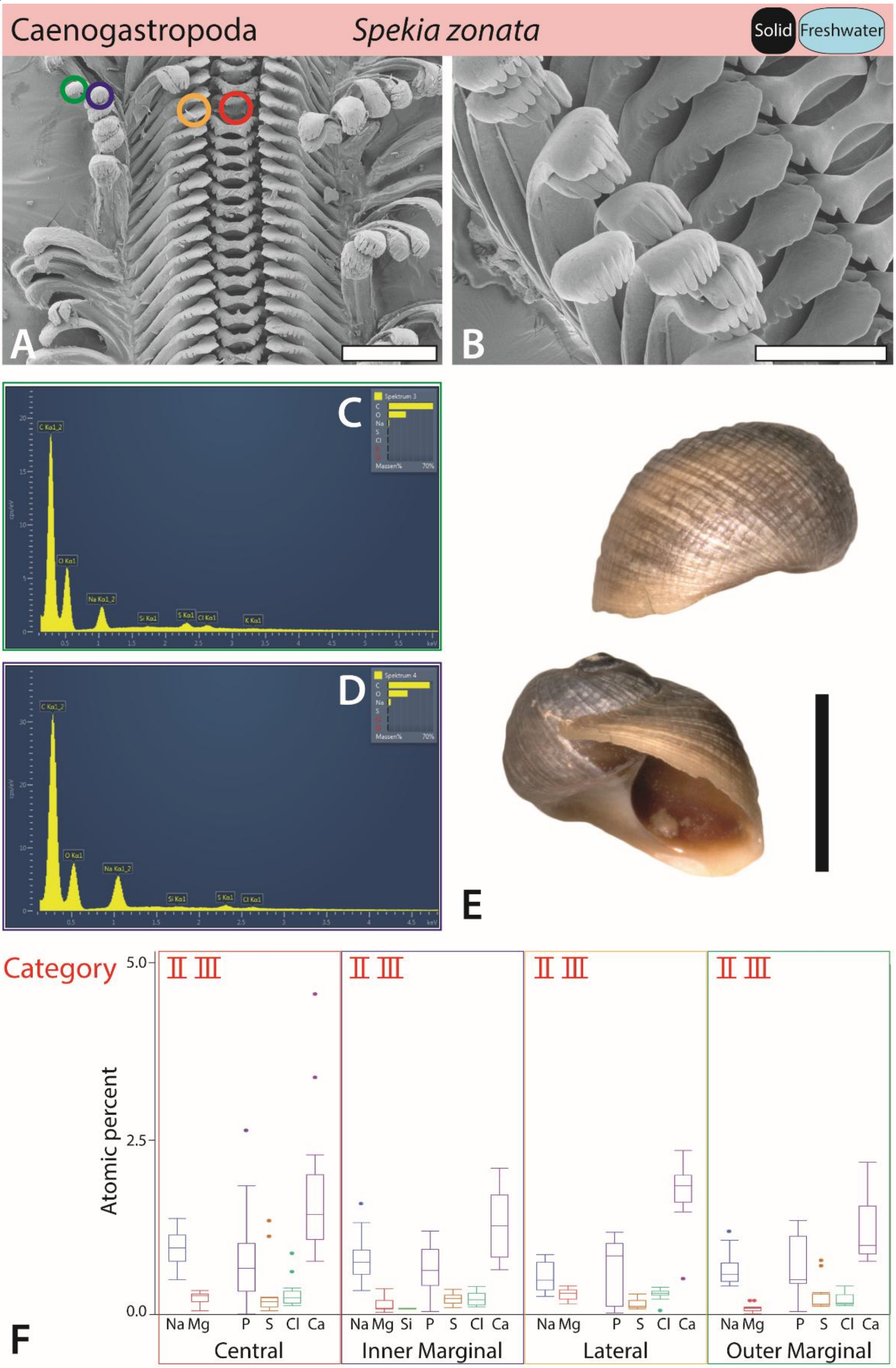

Supplementary Figure 14. ***Spekia zonata***: A-B. SEM images of the working zone of one representative radula (see also 190, 191). The circles indicate the area of the EDX analysis: green, outer marginal; blue, inner marginal; yellow, lateral; red, central teeth. C-D. Representative EDX spectra of outer marginal (C) and inner marginal (D) teeth. E. Habitus from one representative specimen in dorsal and ventral views. F. Results from EDX analyses: elemental proportions, given in atomic percent, for central, inner marginal, lateral, and outer marginal teeth. Proposed biomineralization categories for each tooth type are written in red. Scale bars: A-B, 200 µm; E, 5 mm.

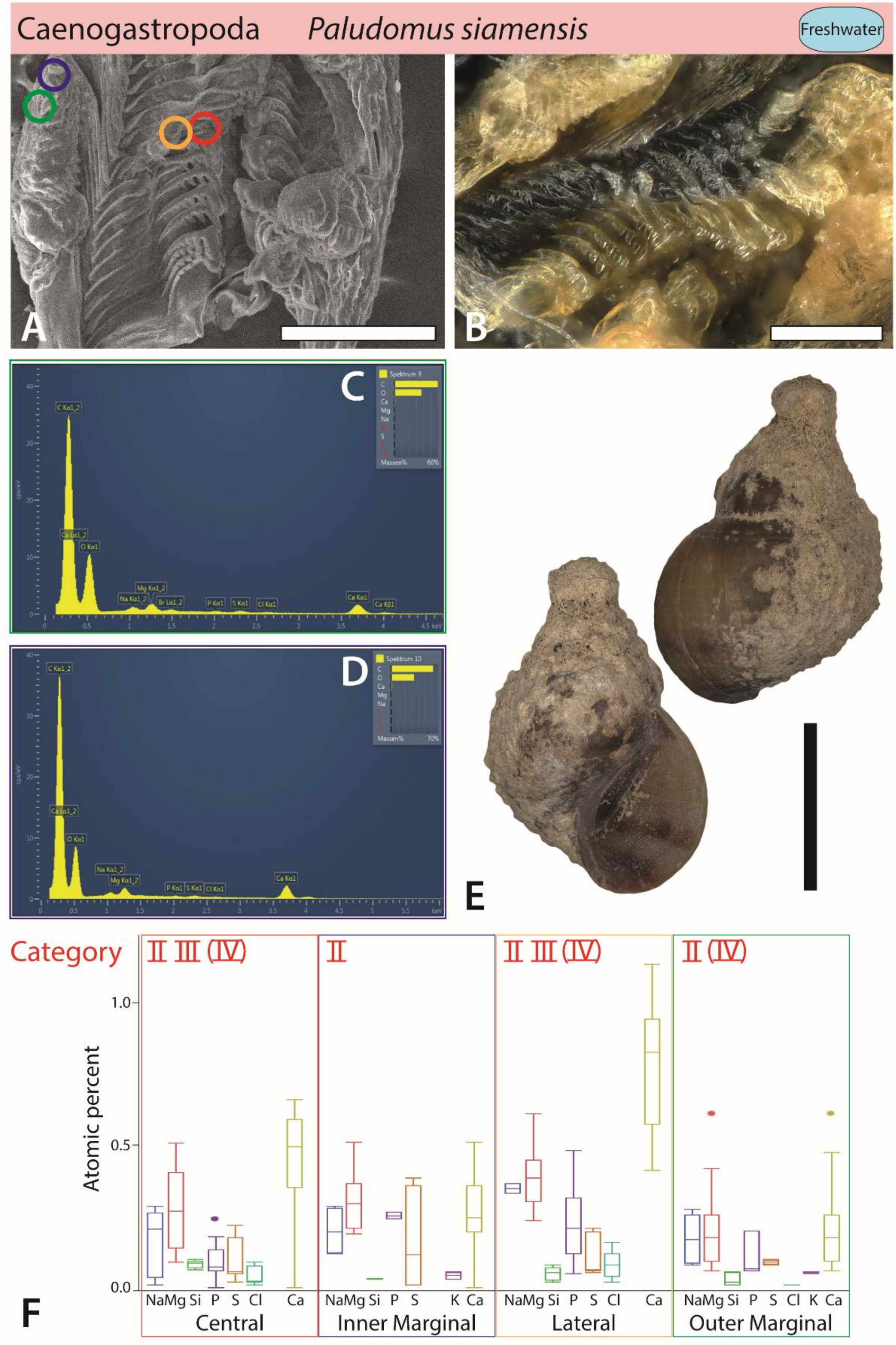

Supplementary Figure 15. *Paludomus siamensis*: A. SEM image and B. picture taken by Keyence of the working zone of one representative radula. The circles indicate the area of the EDX analysis: green, outer marginal; blue, inner marginal; yellow, lateral; red, central teeth. C-D. Representative EDX spectra of outer marginal (C) and inner marginal (D) teeth. E. Habitus from one representative specimen in dorsal and ventral views. F. Results from EDX analyses: elemental proportions, given in atomic percent, for central, inner marginal, lateral, and outer marginal teeth. Proposed biomineralization categories for each tooth type are written in red. Scale bars: A-B, 100 µm; E, 6 mm.

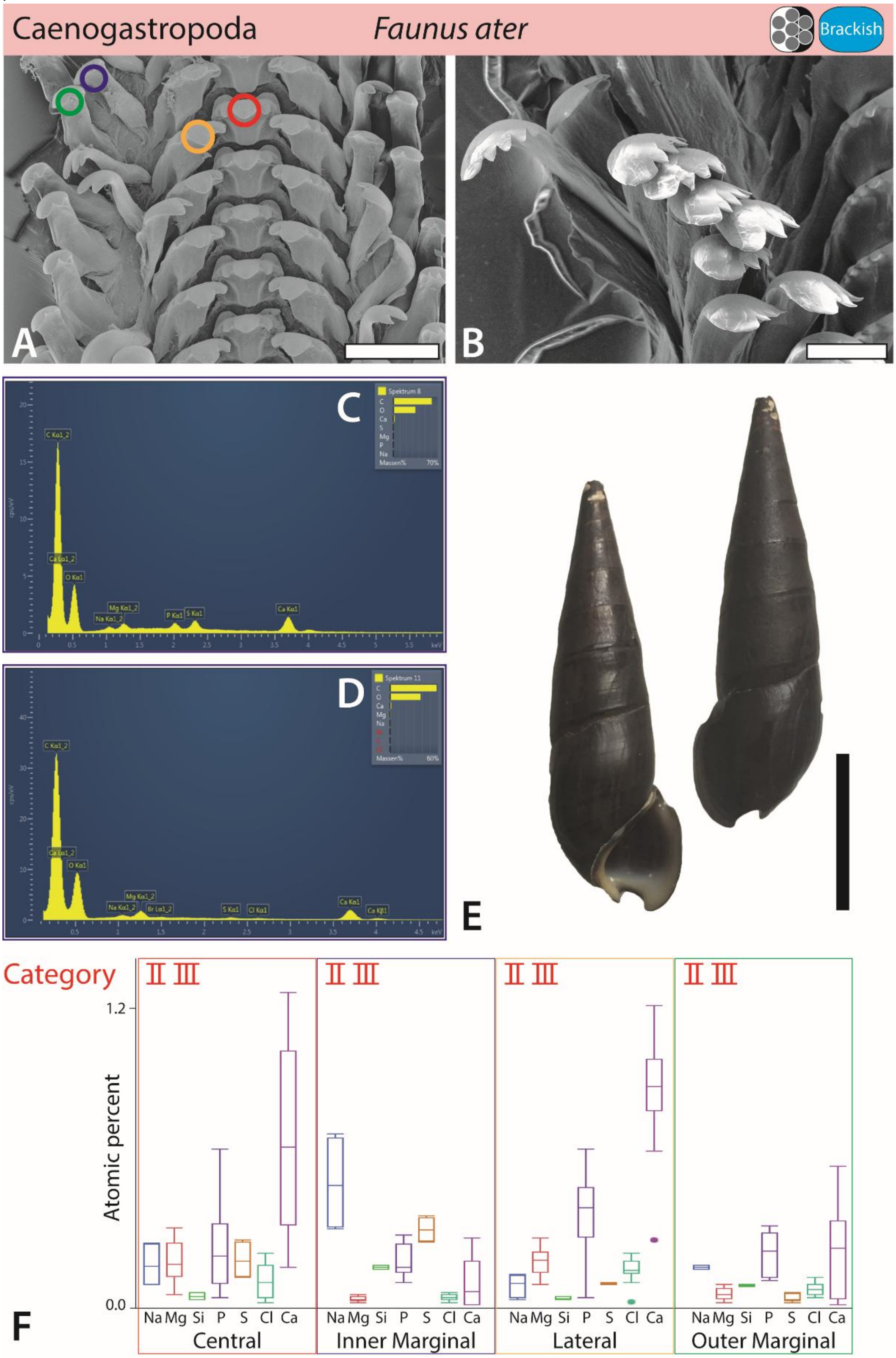

Supplementary Figure 16. ***Faunus ater***: A-B. SEM images of the working zone of one representative radula. The circles indicate the area of the EDX analysis: green, outer marginal; blue, inner marginal; yellow, lateral; red, central teeth. C-D. Representative EDX spectra of outer marginal (C) and inner marginal (D) teeth. E. Habitus from one representative specimen in dorsal and ventral views. F. Results from EDX analyses: elemental proportions, given in atomic percent, for central, inner marginal, lateral, and outer marginal teeth. Proposed biomineralization categories for each tooth type are written in red. Scale bars: A, 200 µm; B, 60 µm; E, 2 cm.

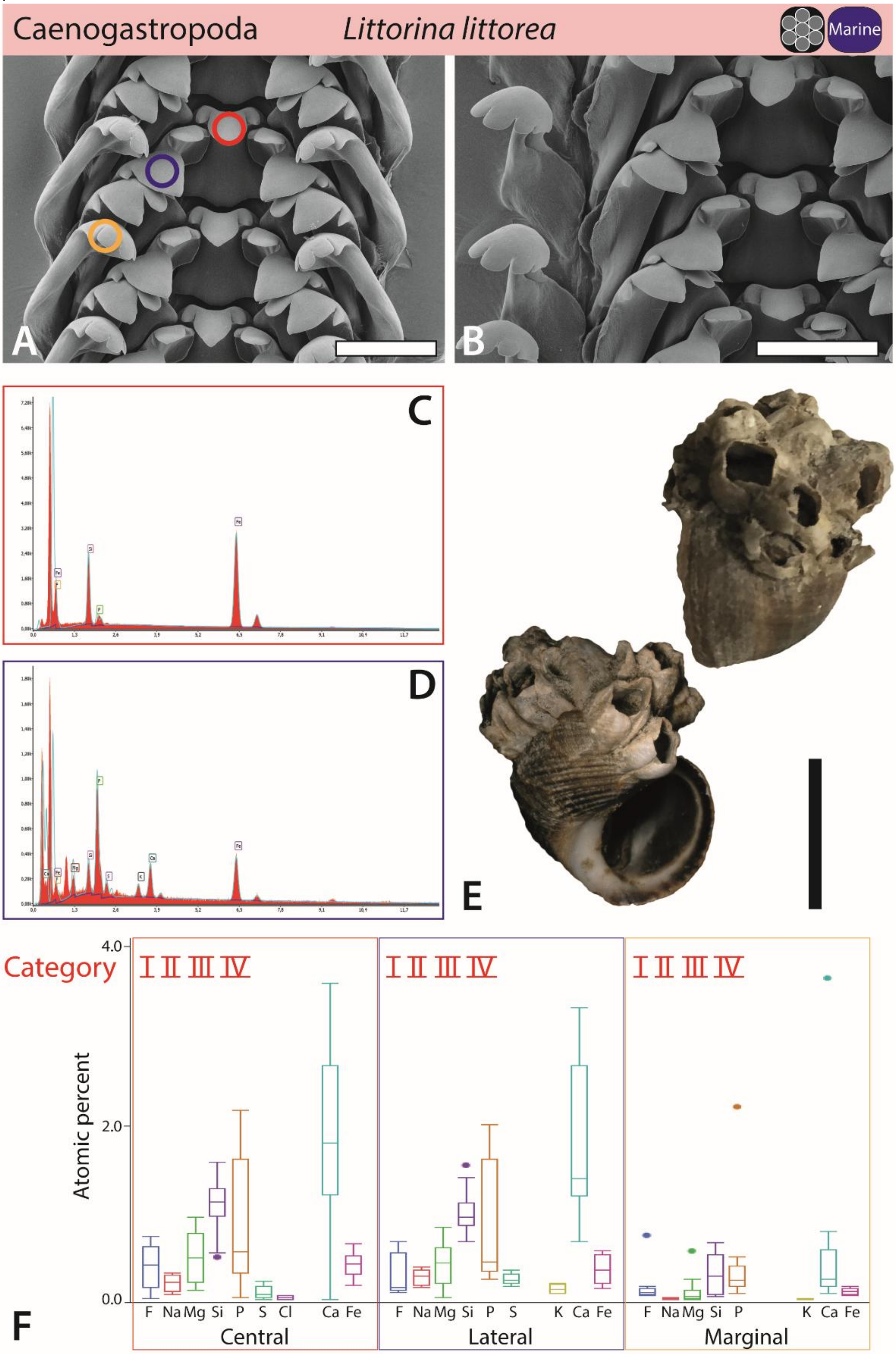

Supplementary Figure 17. ***Littorina littorea***: A-B. SEM images of the working zone of one representative radula (see also 192) . The circles indicate the area of the EDX analysis: yellow, marginal; blue, lateral; red, central teeth. C-D. Representative EDX spectra of central (C) and lateral (D) teeth. E. Habitus from one representative specimen in dorsal and ventral views. F. Results from EDX analyses: elemental proportions, given in atomic percent, for central, lateral, and marginal teeth. Proposed biomineralization categories for each tooth type are written in red. Scale bars: A-B, 80 µm; E, 1 cm.

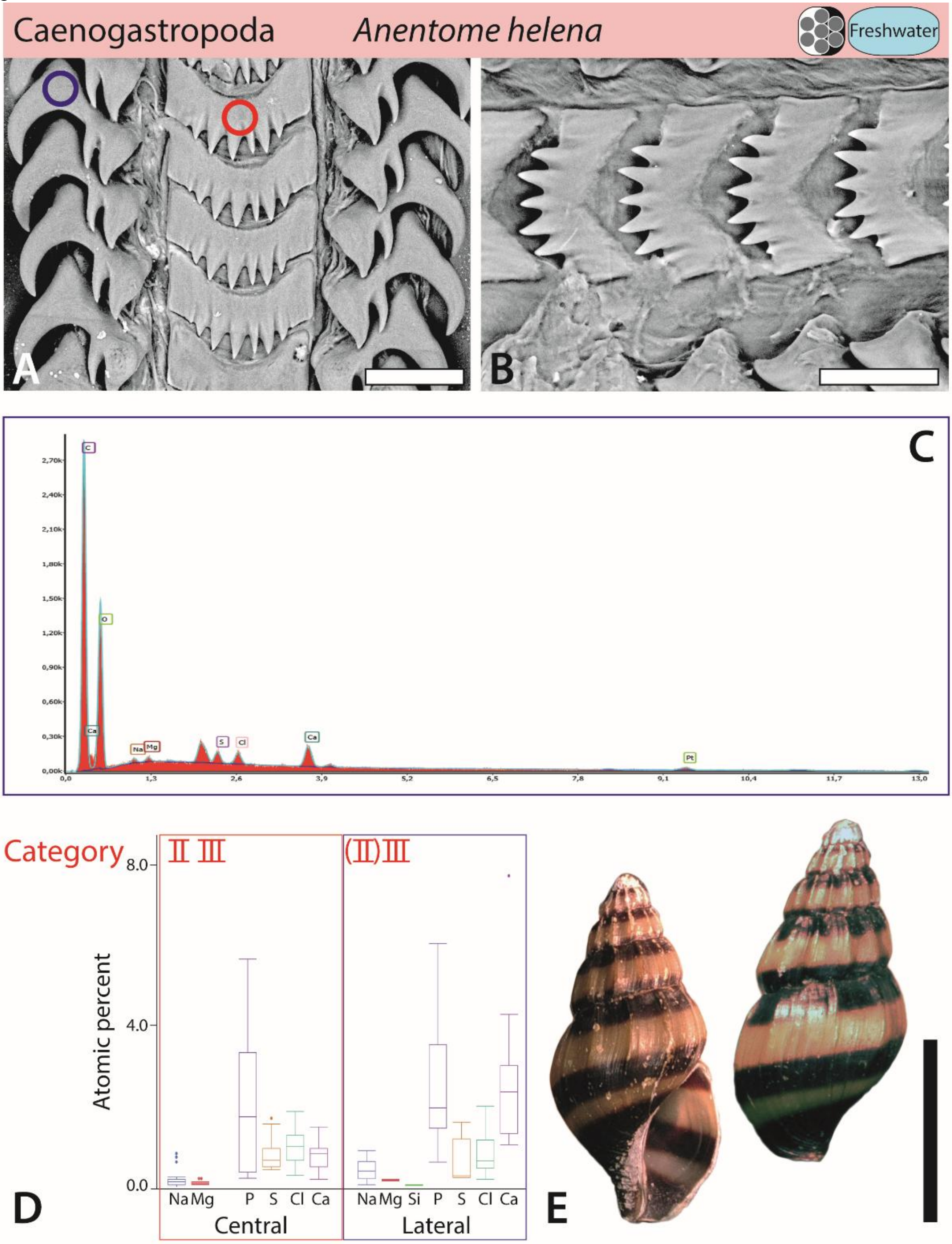

Supplementary Figure 18. ***Anentome helena***: A-B. SEM images of the working zone of one representative radula. The circles indicate the area of the EDX analysis: blue, lateral; red, central teeth. C. Representative EDX spectrum of the lateral tooth. D. Results from EDX analyses: elemental proportions, given in atomic percent, for central and lateral teeth. Proposed biomineralization categories for each tooth type are written in red. E. Habitus from one representative specimen in dorsal and ventral views. Scale bars: A-B, 40 µm; E, 1 cm.

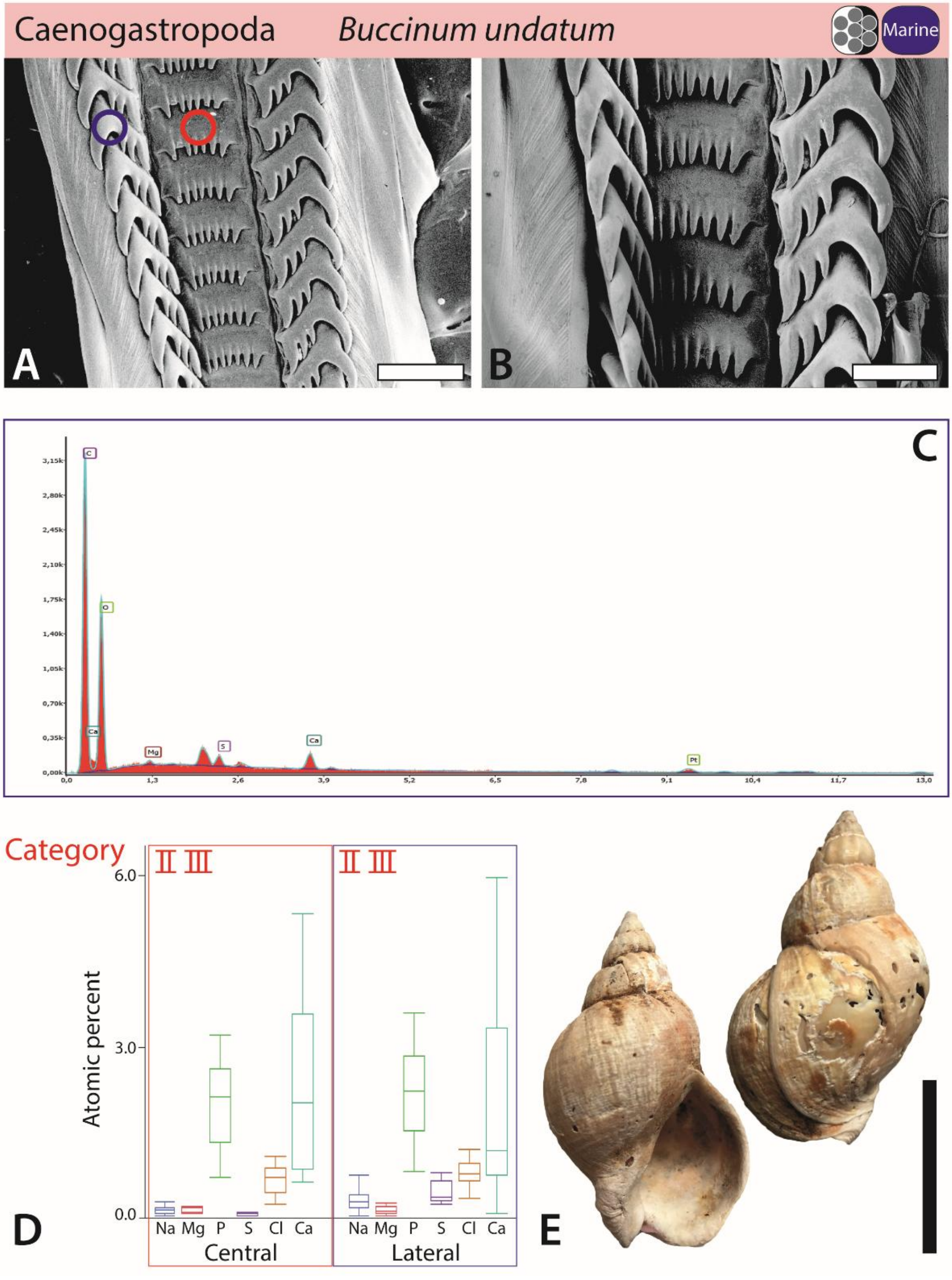

Supplementary Figure 19. ***Buccinum undatum***: A-B. SEM images of the working zone of one representative radula. The circles indicate the area of the EDX analysis: blue, lateral; red, central teeth. C. Representative EDX spectrum of the lateral tooth. D. Results from EDX analyses: elemental proportions, given in atomic percent, for central and lateral teeth. Proposed biomineralization categories for each tooth type are written in red. E. Habitus from one representative specimen in dorsal and ventral views. Scale bars: A, 500 µm; B, 300 µm; E, 3 cm.

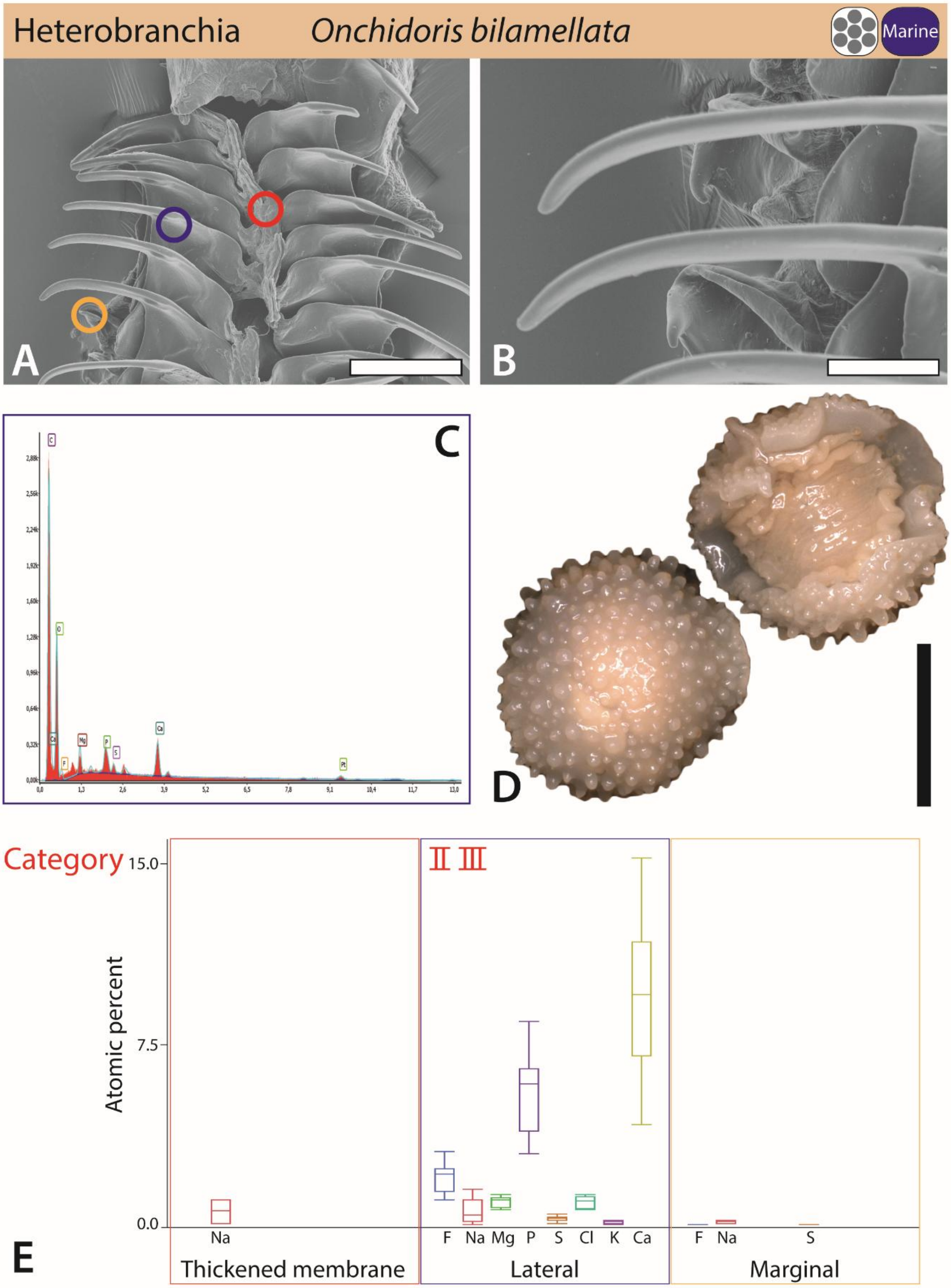

Supplementary Figure 20. *Onchidoris bilamellata*: A-B. SEM images of the working zone of one representative radula. The circles indicate the area of the EDX analysis: yellow, marginal; blue, lateral; red, central teeth. C. Representative EDX spectrum of the lateral tooth. D. Habitus from one representative individual in dorsal and ventral views (images of living gastropods). E. Results from EDX analyses: elemental proportions, given in atomic percent, for central, lateral, and marginal teeth. Proposed biomineralization categories for each tooth type are written in red. Scale bars: A, 150 µm; B, 40 µm; D, 5 mm.

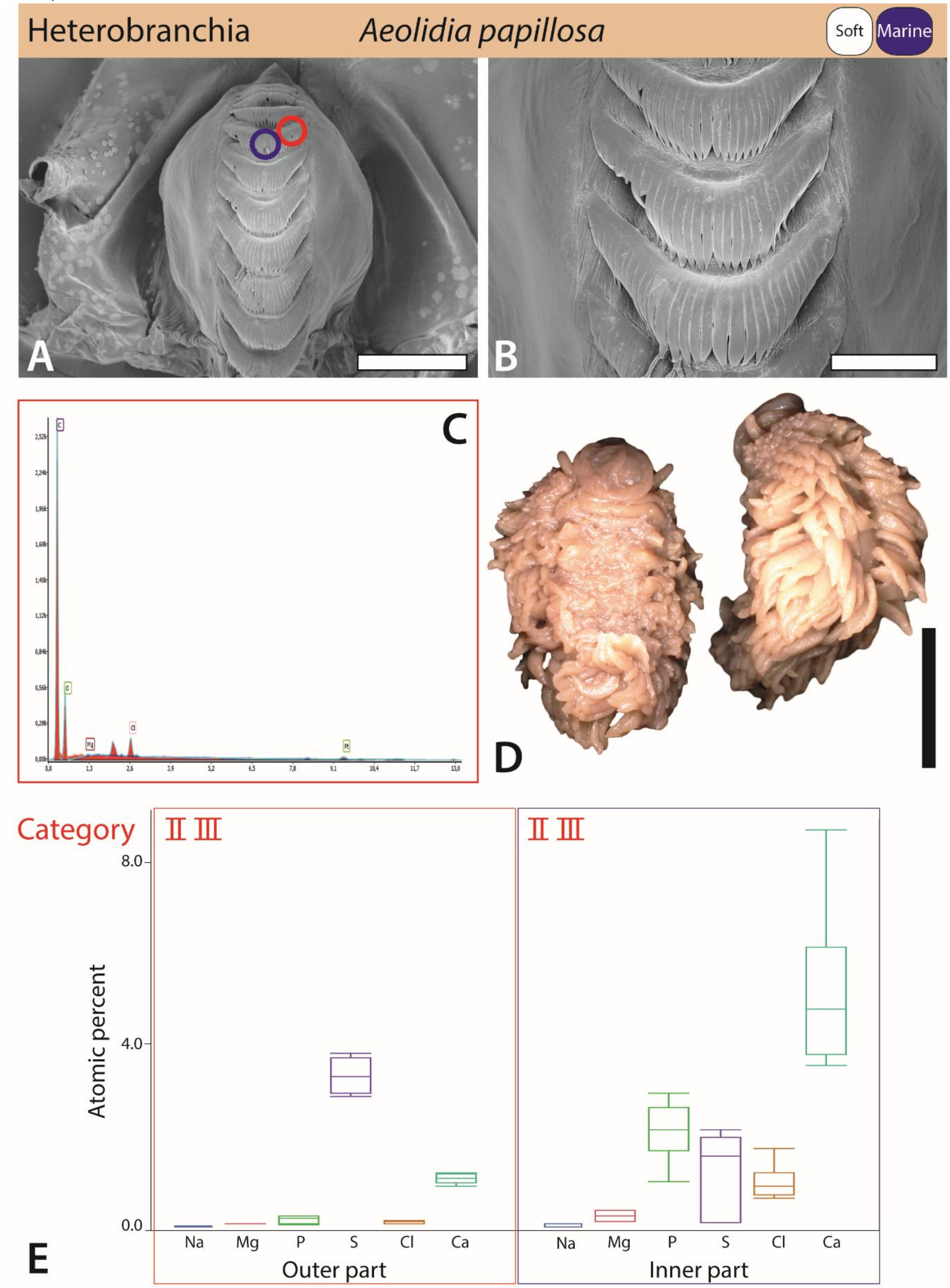

Supplementary Figure 21. ***Aeolidia papillosa***: A-B. SEM images of the working zone of one representative radula. The circles indicate the area of the EDX analysis: blue, inner part; red, outer part. C. Representative EDX spectrum of the outer tooth part. D. Habitus from one representative specimen in dorsal and ventral views. E. Results from EDX analyses: elemental proportions, given in atomic percent, for outer and inner tooth part. Proposed biomineralization categories for each tooth part are written in red. Scale bars: A, 400 µm; B, 150 µm; D, 7 mm.

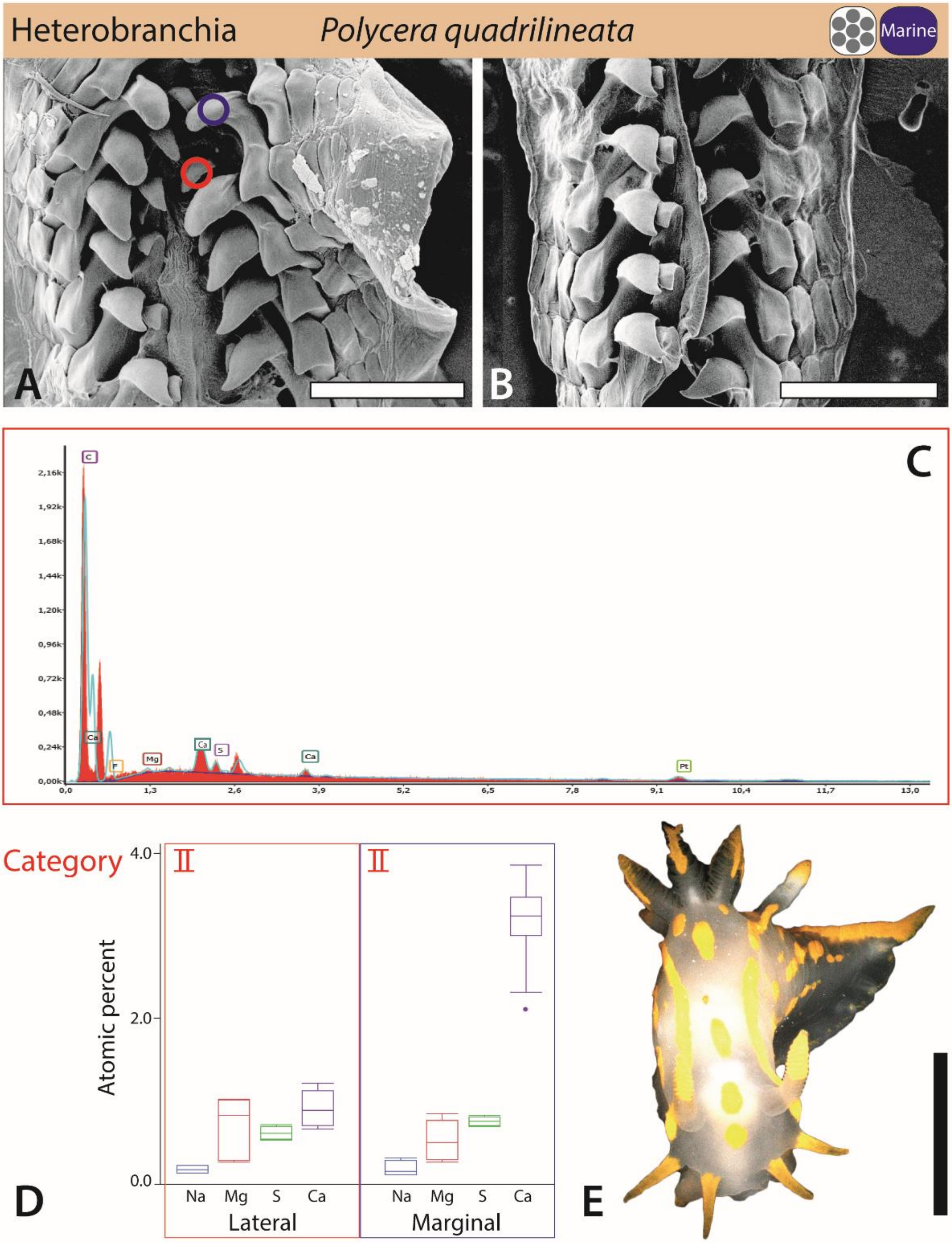

Supplementary Figure 22. ***Polycera quadrilineata***: A-B. SEM images of the working zone of one representative radula. The circles indicate the area of the EDX analysis: red, lateral; blue, marginal teeth. C. Representative EDX spectrum of the lateral tooth. D. Results from EDX analyses: elemental proportions, given in atomic percent, for lateral and marginal teeth. Proposed biomineralization categories for each tooth type are written in red. E. Habitus from one representative individual (image of the living animal). Scale bars: A-B, 400 µm; E, 1 cm.

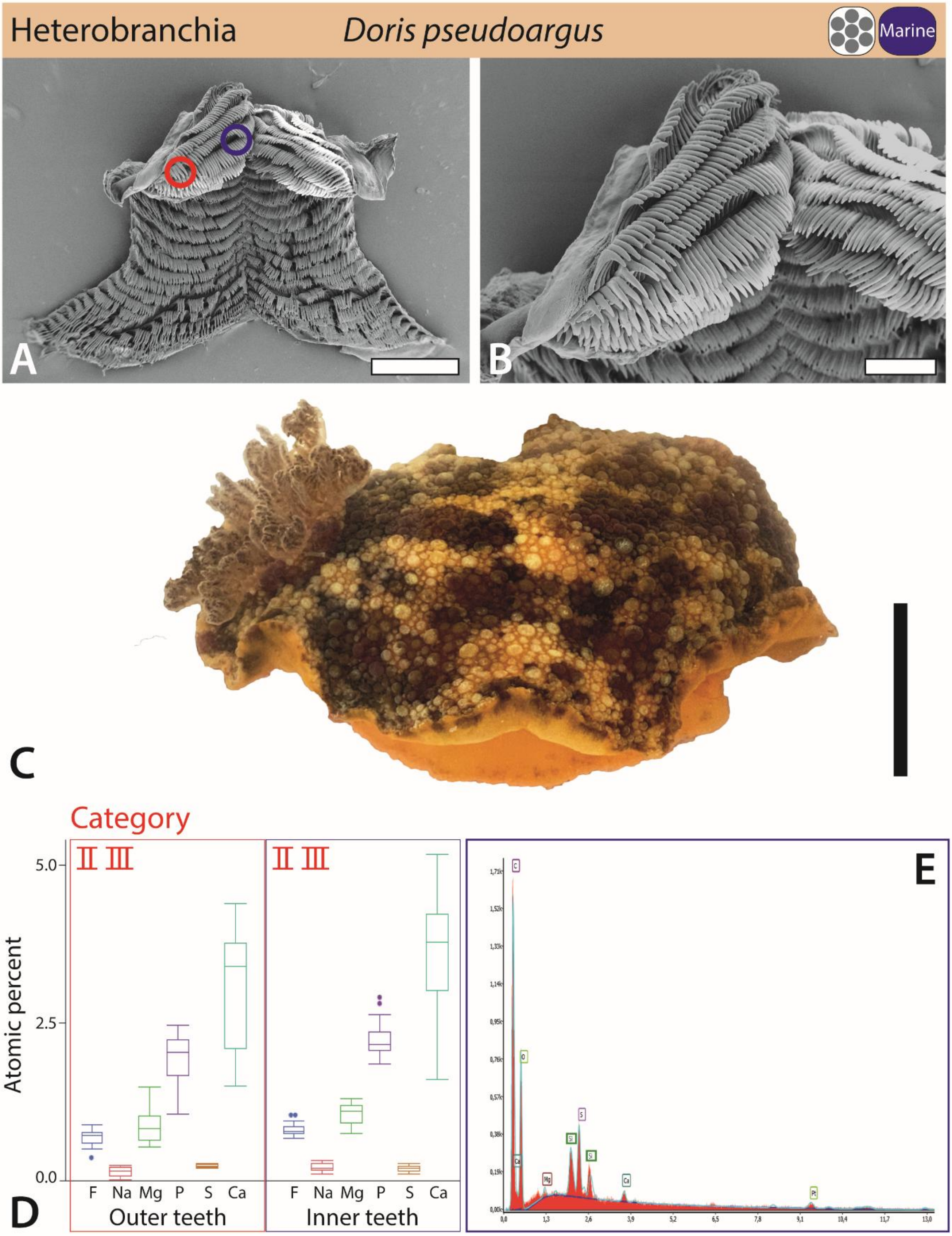

Supplementary Figure 23. ***Doris pseudoargus***: A-B. SEM images of the working zone of one representative radula. The circles indicate the area of the EDX analysis: red, outer teeth; blue, inner teeth. C. Habitus from one representative individual (image of the living animal). D. Results from EDX analyses: elemental proportions, given in atomic percent, for outer and inner teeth. Proposed biomineralization categories for each tooth type are written in red. E. Representative EDX spectrum of the inner teeth. Scale bars: A, 900 µm; B, 300 µm; C, 1 cm.

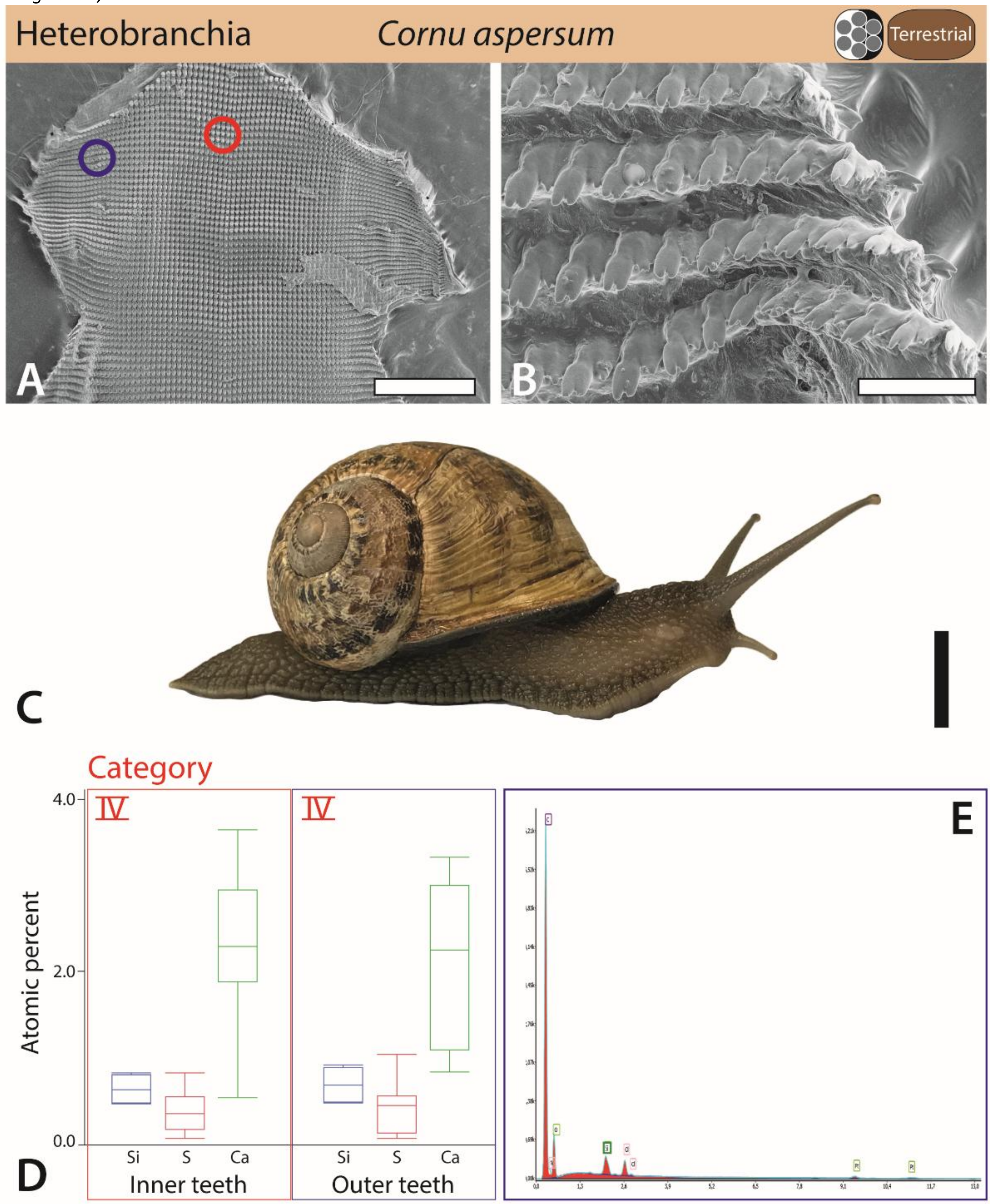

Supplementary Figure 24. ***Cornu aspersum***: A-B. SEM images of the working zone of one representative radula. The circles indicate the area of the EDX analysis: red, inner teeth; blue, outer teeth. C. Habitus from one representative individual (image of the living animal). D. Results from EDX analyses: elemental proportions, given in atomic percent, for inner and outer teeth. Proposed biomineralization categories for each tooth type are written in red. E. Representative EDX spectrum of outer teeth. Scale bars: A, 800 µm; B, 60 µm; C, 1 cm.

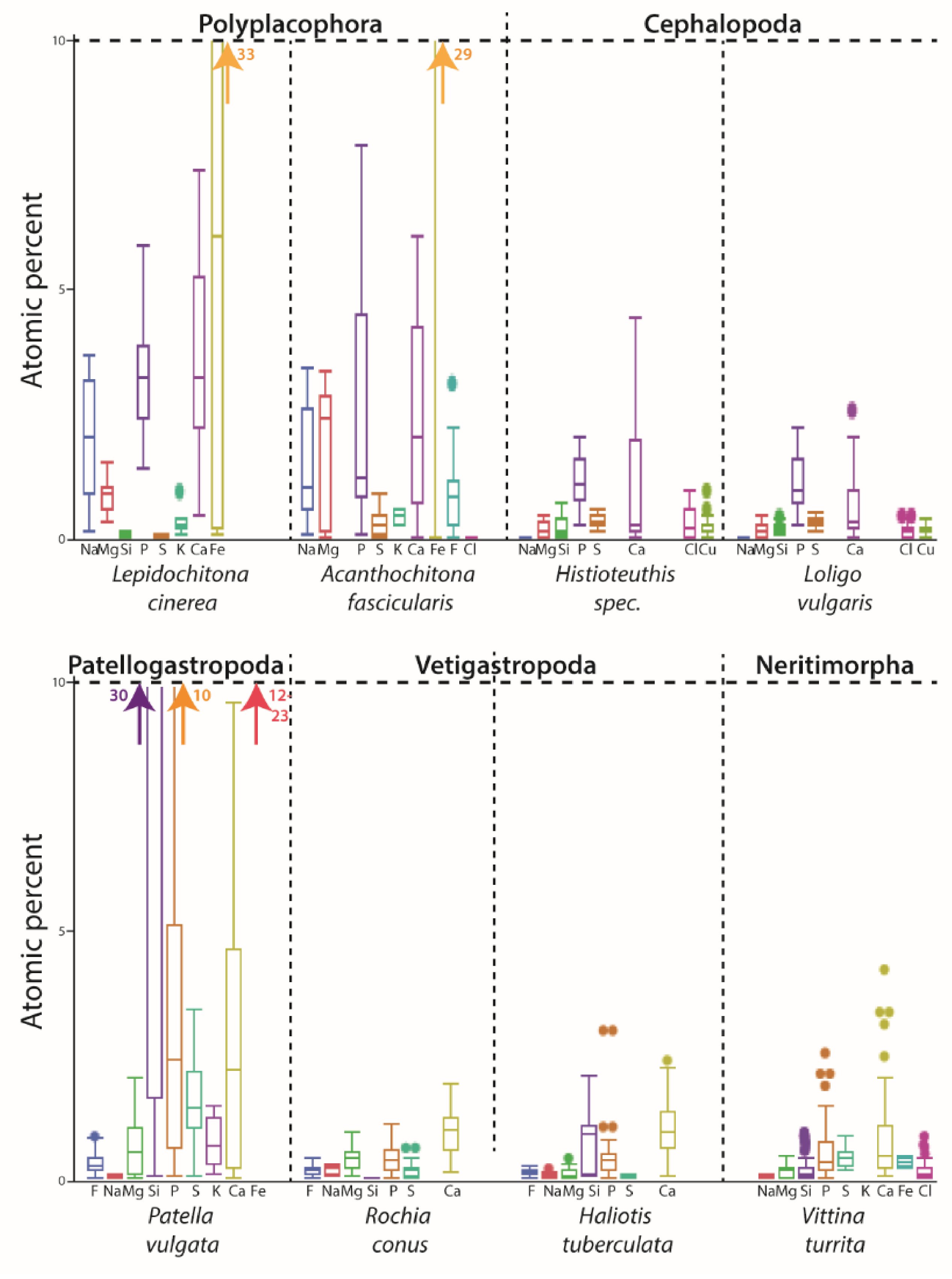

Supplementary Figure 25. Results from EDX analyses, given in atomic percent, summarized for each species. To enable comparison, the scale is identical to the scales in Supplementary Figures 26 and 27.

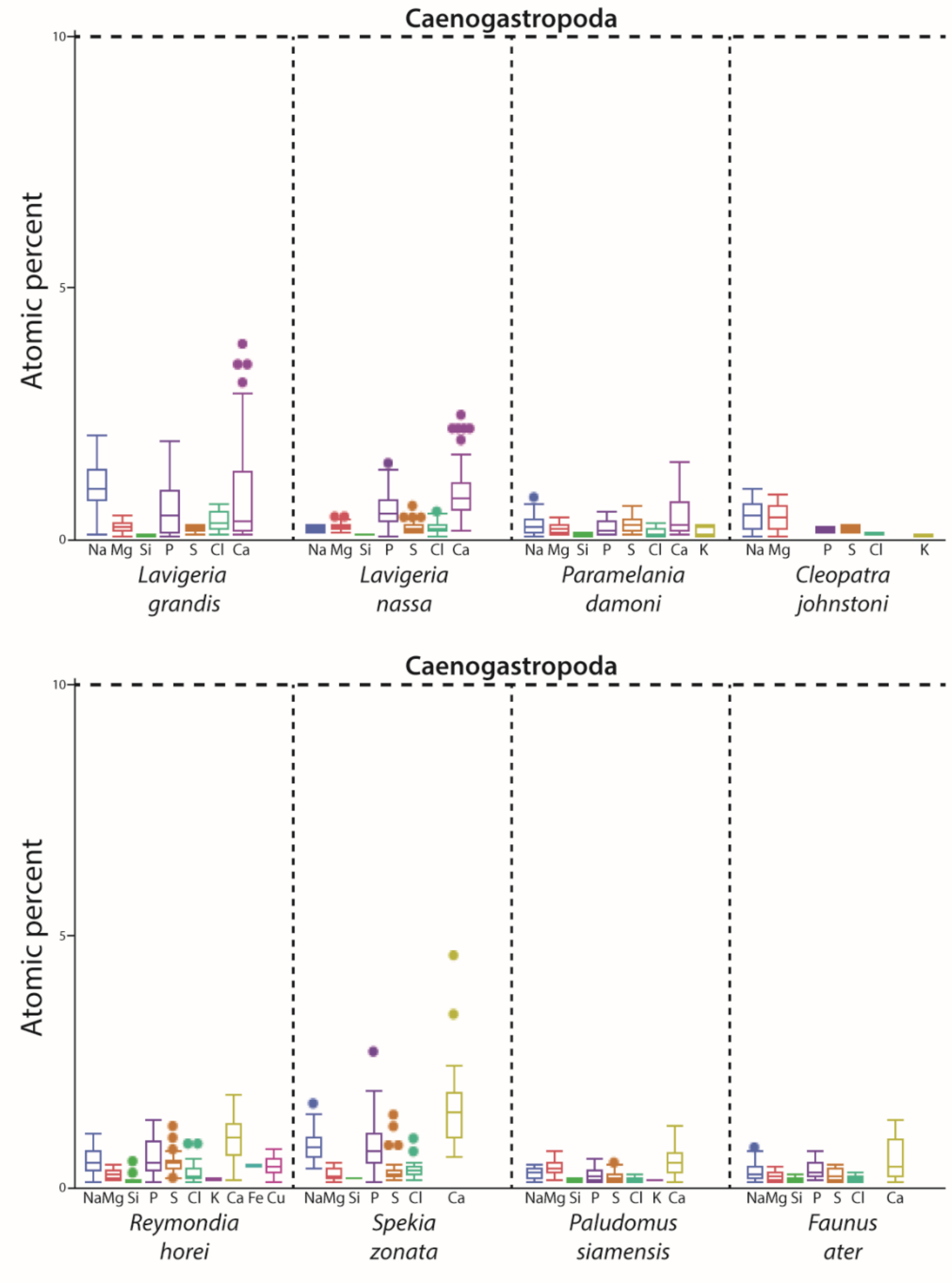

Supplementary Figure 26. Results from EDX analyses, given in atomic percent, summarized for each species. To enable comparison, the scale is identical to the scales in Supplementary Figures 25 and 27.

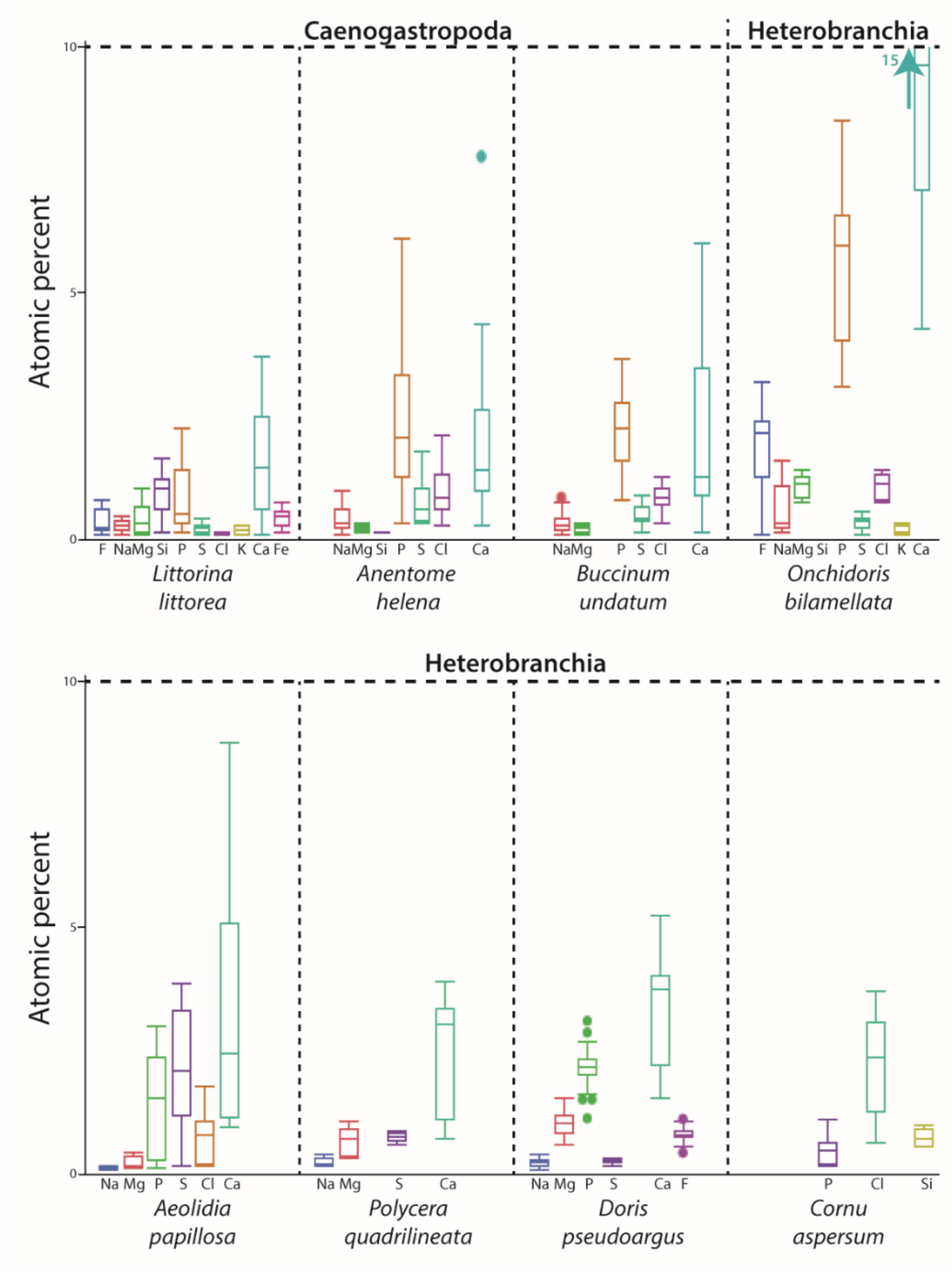

Supplementary Figure 27. Results from EDX analyses, given in atomic percent, summarized for each species. To enable comparison, the scale is identical to the scales in Supplementary Figures 25 and 26.

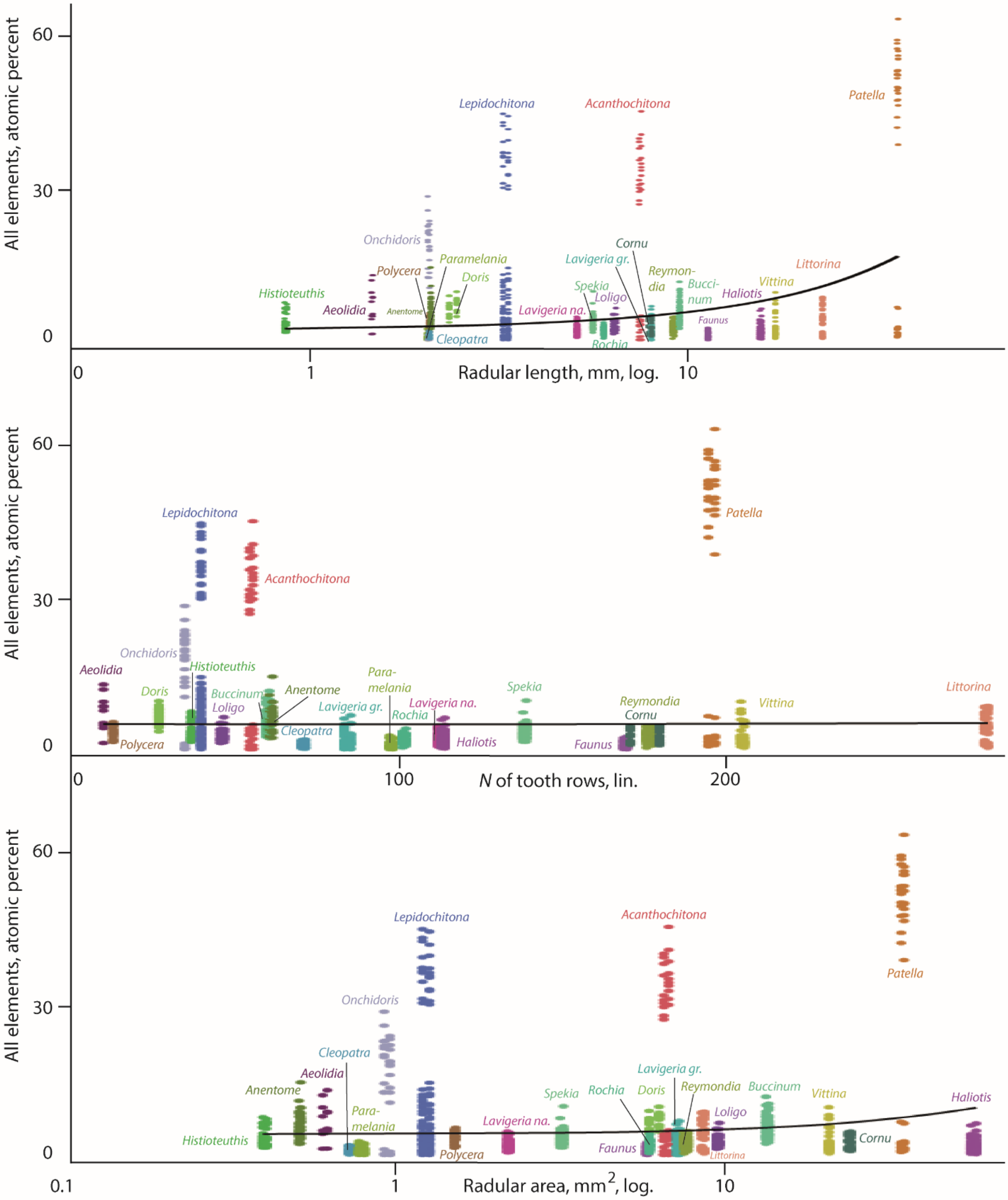

Supplementary Figure 28. Radular parameters (area, length, quantity of tooth rows) plotted against the proportions of all elements studied.

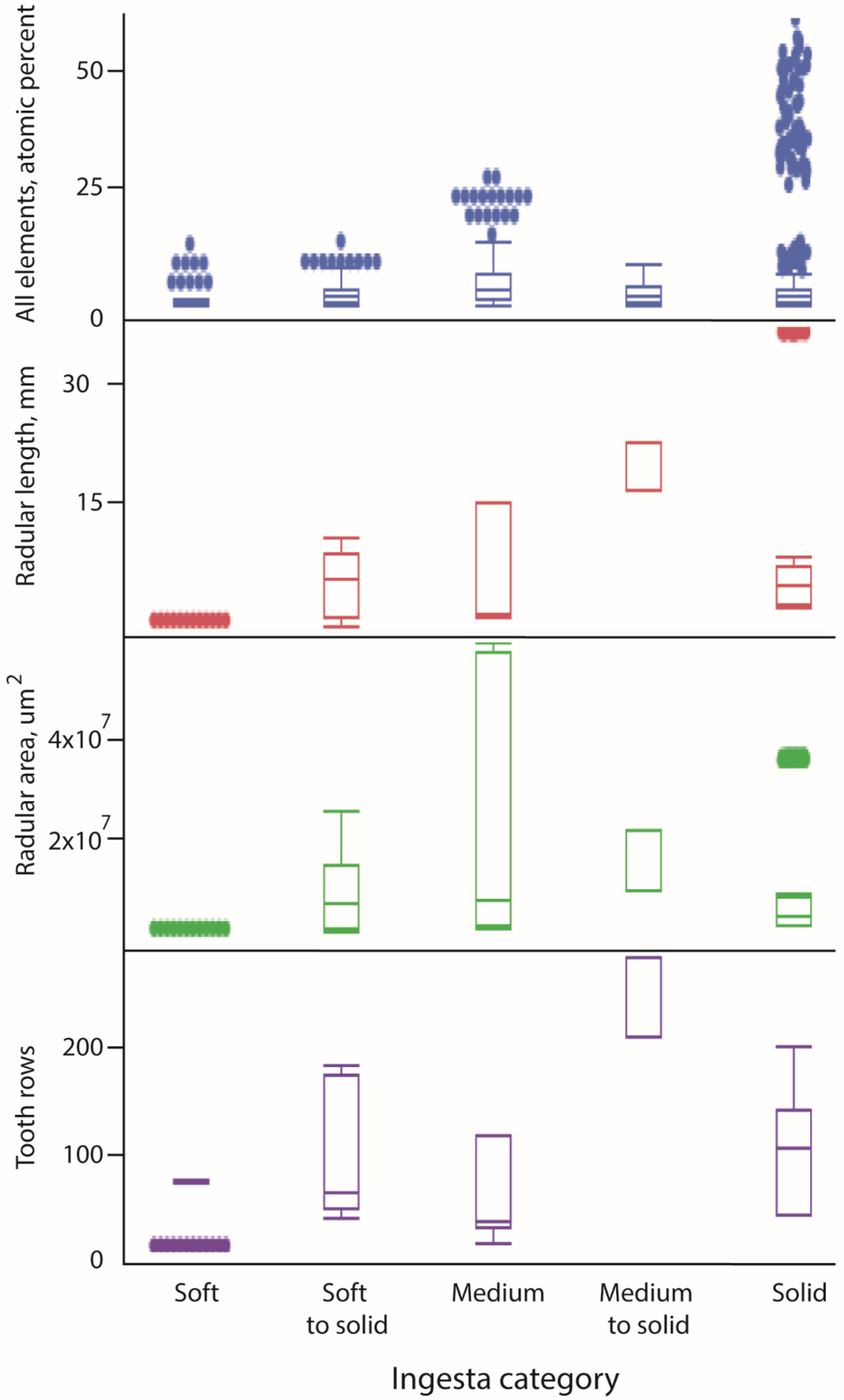

Supplementary Figure 29. Ingesta categories plotted against radular parameters (area, length, quantity of tooth rows) and the proportions of all elements studied.

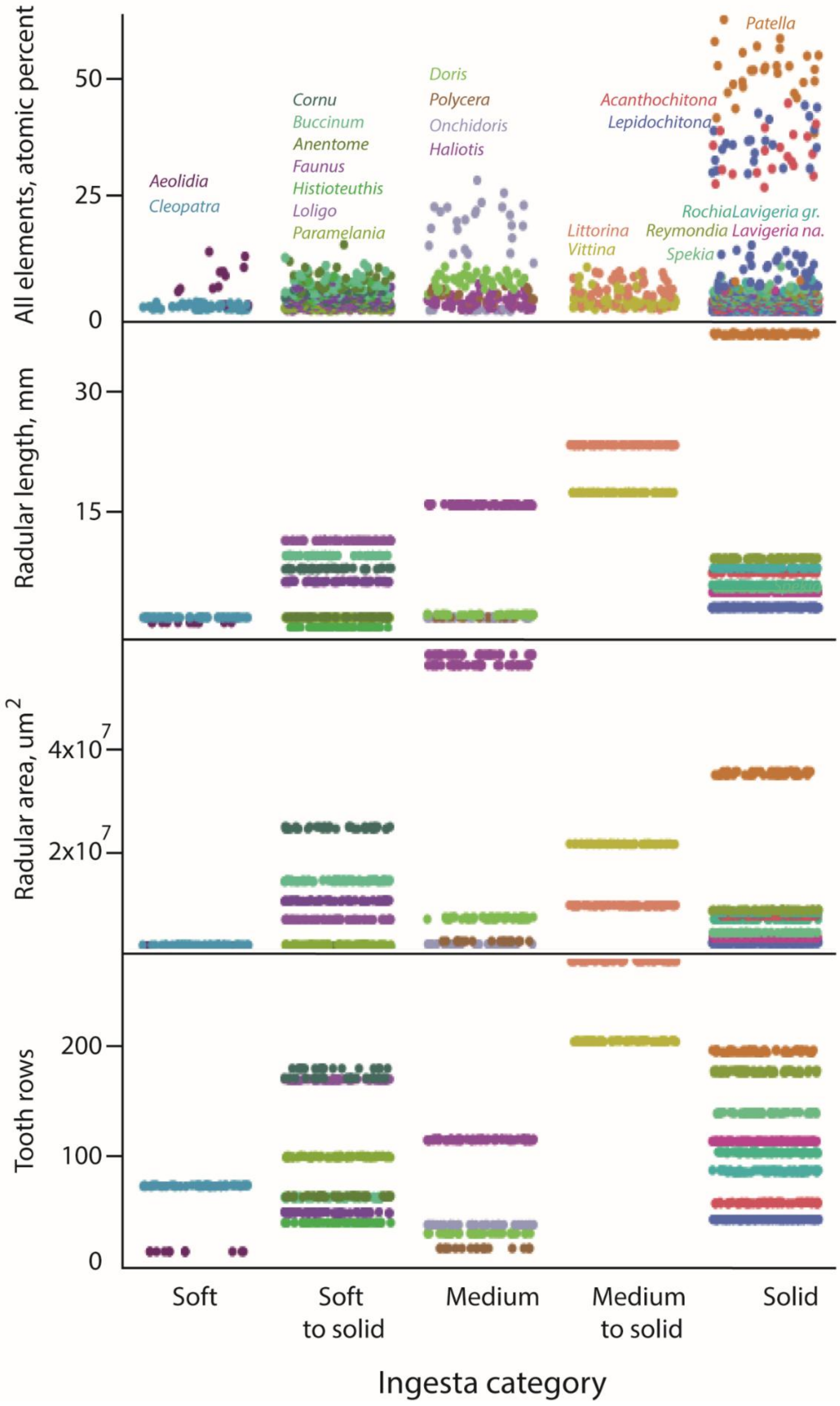

Supplementary Figure 30. Ingesta categories plotted against radular parameters (area, length, quantity of tooth rows) and the proportions of all elements studied. Here, the data on individual species can be seen.

Supplementary Table 1. For all species together: Mean, given in atomic percent, SD, and N for the individual elements studied. N, quantity of teeth that contain the element.

Element	Mean, atomic %	SD	*N* (overall *N* = 1448 studied teeth)
Fe	9.14	10.41	182
Si	1.97	5.76	365
Ca	1.72	1.95	1220
P	1.16	1.50	974
F	0.48	0.60	336
Na	0.39	0.51	619
Mg	0.36	0.48	961
S	0.35	0.44	831
Cl	0.29	0.32	638
Cu	0.24	0.16	146
K	0.19	0.30	100

Supplementary Table 2. For each larger taxon: Sum of means, given in atomic percent, of the individual elements.

Taxon	Sum of means, in atomic %
Patellogastropoda	40.95 %
Polyplacophora	24.01 %
Heterobranchia	11.38 %
Caenogastropoda	4.40 %
Cephalopoda	3.15 %
Vetigastropoda	2.72 %
Neritimorpha	2.69 %

Supplementary Table 3. Proportions of the individual elements, given in atomic percent, (mean, SD, sum of means, N) for each species. N, quantity of teeth that contain the element.

Species	Tooth	*N* measurements total	Element																																			
			All elements			F			Na			Mg			Si			P			S			Cl			K			Ca			Fe			Cu		
			Mean	SD	Sum of means	Mean	SD	*N*	Mean	SD	*N*	Mean	SD	*N*	Mean	SD	*N*	Mean	SD	*N*	Mean	SD	*N*	Mean	SD	*N*	Mean	SD	*N*	Mean	SD	*N*	Mean	SD	*N*	Mean	SD	*N*
Lepidochitona cinerea	**All teeth**	**212**	6.21	11.05	21.75	0.00	0.00	0	1.96	1.21	11	0.86	0.34	10	0.10	0.03	5	3.37	1.39	11	0.05	0.02	6	0.00	0.00	0	0.32	0.20	14	3.64	1.84	128	11.45	11.91	68	0.00	0.00	0
	**Central**	**23**			4.09	0.00	0.00	0	0.00	0.00	0	0.00	0.00	0	0.00	0.00	0	0.00	0.00	0.00	0.00	0.00	0	0.00	0.00	0	0.56	0.51	2	1.88	0.94	16	1.65	0.00	1	0.00	0.00	0
	**Lateral I**	**47**			2.74	0.00	0.00	0	0.00	0.00	0	0.00	0.00	0	0.10	0.00	1	0.00	0.00	0.00	0.00	0.00	0	0.00	0.00	0	0.00	0.00	0	2.64	1.33	20	0.00	0.00	0	0.00	0.00	0
	**Lateral II**	**71**			23.24	0.00	0.00	0	1.96	1.21	11	0.86	0.34	10	0.09	0.05	3	3.37	1.39	11	0.05	0.02	6	0.00	0.00	0	0.29	0.13	10	4.68	1.42	71	11.94	11.96	65	0.00	0.00	0
	**Marginal**	**71**			3.22	0.00	0.00	0	0.00	0.00	0	0.00	0.00	0	0.12	0.00	1	0.00	0.00	0.00	0.00	0.00	0	0.00	0.00	0	0.25	0.00	2	2.40	1.74	21	0.45	0.04	2	0.00	0.00	0
Acanthochitona fascicularis	**All teeth**	**58**	12.40	16.04	28.04	0.90	0.75	36	1.44	1.21	20	1.65	1.26	39	0.00	0.00	0	2.62	2.25	34	0.32	0.29	26	0.02	0.00	2	0.44	0.17	4	2.34	1.97	39	18.31	7.60	22	0.00	0.00	0
	**Central**	**10**			0.79	0.01	0.01	5	0.00	0.00	0	0.03	0.02	5	0.00	0.00	0	0.70	0.05	2	0.04	0.02	4	0.00	0.00	0	0.00	0.00	0	0.01	0.01	3	0.00	0.00	0	0.00	0.00	0
	**Lateral I**	**12**			3.54	0.79	0.34	12	0.54	0.28	6	0.64	0.43	12	0.00	0.00	0	0.67	0.43	12	0.09	0.03	4	0.00	0.00	0	0.00	0.00	0	0.78	0.36	12	0.03	0.00	2	0.00	0.00	0
	**Lateral II**	**20**			35.22	1.21	0.84	19	2.12	0.92	12	2.81	0.26	20	0.00	0.00	0	3.98	1.99	20	0.43	0.28	18	0.00	0.00	0	0.44	0.17	4	4.09	1.00	20	20.14	5.01	20	0.00	0.00	0
	**Marginal**	**16**			0.28	0.00	0.00	0	0.06	0.00	2	0.15	0.01	2	0.00	0.00	0	0.00	0.00	0.00	0.00	0.00	0	0.02	0.00	2	0.00	0.00	0	0.05	0.01	4	0.00	0.00	0	0.00	0.00	0
Histioteuthis spec.	**All teeth**	**46**	3.41	1.73	3.48	0.00	0.00	0	0.02	1.12	15	0.19	0.15	46	0.26	0.21	36	1.14	0.47	46	0.34	0.11	46	0.32	0.29	46	0.00	0.00	0	1.01	1.31	46	0.00	0.00	0	0.20	0.15	46
	**Central**	**8**			3.36	0.00	0.00	0	0.02	0.00	2	0.13	0.19	8	0.16	0.13	8	1.09	0.46	8	0.32	0.11	8	0.21	0.17	8	0.00	0.00	0	1.07	0.85	8	0.00	0.00	0	0.36	0.28	8
	**Lateral**	**12**			2.56	0.00	0.00	0	0.02	0.01	5	0.08	0.08	12	0.18	0.06	3	1.06	0.46	12	0.45	0.08	12	0.34	0.26	12	0.00	0.00	0	0.26	0.13	12	0.00	0.00	0	0.17	0.03	12
	**Outer Marginal**	**26**			3.91	0.00	0.00	0	0.02	0.01	8	0.25	0.12	26	0.30	0.23	25	1.19	0.48	26	0.30	0.10	26	0.34	0.33	26	0.00	0.00	0	1.34	1.58	26	0.00	0.00	0	0.17	0.09	26
Loligo vulgaris	**All teeth**	**48**	2.82	1.10	2.83	0.00	0.00	0	0.02	0.01	15	0.17	0.15	48	0.20	0.08	48	1.16	0.54	48	0.33	0.10	48	0.14	0.11	48	0.00	0.00	0	0.63	0.54	48	0.00	0.00	0	0.18	0.10	48
	**Central**	**6**			2.41	0.00	0.00	0	0.02	0.01	2	0.09	0.12	6	0.27	0.07	6	0.81	0.27	6	0.23	0.03	6	0.12	0.06	6	0.00	0.00	0	0.69	0.39	6	0.00	0.00	0	0.18	0.13	6
	**Lateral**	**12**			2.28	0.00	0.00	0	0.02	0.01	4	0.03	0.01	12	0.14	0.06	12	0.99	0.50	12	0.40	0.07	12	0.12	0.14	12	0.00	0.00	0	0.42	0.52	12	0.00	0.00	0	0.16	0.05	12
	**Inner marginal**	**14**			3.25	0.00	0.00	0	0.02	0.01	5	0.25	0.13	14	0.24	0.08	14	1.38	0.54	14	0.33	0.10	14	0.15	0.13	14	0.00	0.00	0	0.69	0.70	14	0.00	0.00	0	0.19	0.10	14
	**Outer marginal**	**16**			3.05	0.00	0.00	0	0.02	0.01	4	0.23	0.14	16	0.19	0.06	16	1.23	0.56	16	0.32	0.09	16	0.17	0.08	16	0.00	0.00	0	0.70	0.44	16	0.00	0.00	0	0.19	0.11	16
Patella vulgata	**All teeth**	**50**	29.64	25.11	40.95	0.32	0.21	50	0.03	0.01	25	0.64	0.58	49	15.28	10.20	40	3.16	2.86	50	1.56	0.87	36	0.00	0.00	0	0.72	0.54	12	2.77	2.77	50	16.47	2.79	28	0.00	0.00	0
	**Lateral**	**14**			52.49	0.40	0.24	14	0.02	0.01	6	0.90	0.34	14	21.47	4.19	14	5.00	2.56	14	2.04	0.65	14	0.00	0.00	0	0.47	0.45	6	4.68	2.22	14	17.51	2.89	14	0.00	0.00	0
	**Dominant tooth**	**14**			51.12	0.40	0.21	14	0.05	0.02	7	1.20	0.51	14	21.33	4.98	14	5.23	2.28	14	1.66	0.77	14	0.00	0.00	0	0.96	0.54	6	4.87	2.07	14	15.42	2.33	14	0.00	0.00	0
	**Marginal**	**22**			2.79	0.21	0.12	22	0.03	0.02	12	0.10	0.06	21	1.01	1.11	12	0.67	0.42	22	0.55	0.52	8	0.00	0.00	0	0.00	0.00	0	0.22	0.17	22	0.00	0.00	0	0.00	0.00	0
Rochia	**All teeth**	**70**	2.04	0.68	2.32	0.17	0.09	70	0.19	0.02	14	0.42	0.22	70	0.02	0.01	4	0.40	0.25	70	0.17	0.15	29	0.00	0.00	0	0.00	0.00	0	0.95	0.44	70	0.00	0.00	0	0.00	0.00	0

conus	**Central**	**8**			2.49	0.26	0.10	8	0.24	0.04	4	0.43	0.22	8	0.00	0.00	0	0.46	0.44	8	0.13	0.06	4	0.00	0.00	0	0.00	0.00	0	0.97	0.43	8	0.00	0.00	0	0.00	0.00	0
	**Lateral**	**18**			2.13	0.13	0.05	18	0.07	0.01	5	0.49	0.11	18	0.00	0.00	0	0.35	0.17	18	0.16	0.10	6	0.00	0.00	0	0.00	0.00	0	0.93	0.35	18	0.00	0.00	0	0.00	0.00	0
	**Inner marginal**	**22**			2.65	0.22	0.08	22	0.20	0.02	4	0.43	0.30	22	0.02	0.00	2	0.47	0.28	22	0.13	0.12	9	0.00	0.00	0	0.00	0.00	0	1.18	0.47	22	0.00	0.00	0	0.00	0.00	0
	**Outer marginal**	**22**			2.04	0.12	0.06	22	0.27	0.02	2	0.35	0.19	22	0.01	0.00	2	0.34	0.19	22	0.23	0.22	10	0.00	0.00	0	0.00	0.00	0	0.72	0.39	22	0.00	0.00	0	0.00	0.00	0
	**All teeth**	**66**	1.91	1.30	2.49	0.13	0.06	66	0.05	0.08	24	0.09	0.10	30	0.74	0.58	24	0.44	0.50	66	0.05	0.03	23	0.00	0.00	0	0.00	0.00	0	0.99	0.58	66	0.00	0.00	0	0.00	0.00	0
	**Central**	**18**			3.52	0.14	0.06	18	0.07	0.04	7	0.12	0.09	18	0.80	0.62	18	0.69	0.85	18	0.07	0.02	5	0.00	0.00	0	0.00	0.00	0	1.63	0.41	18	0.00	0.00	0	0.00	0.00	0
Haliotis tuberculata	**Lateral**	**10**			2.68	0.14	0.06	10	0.07	0.02	3	0.04	0.02	4	0.58	0.41	6	0.48	0.31	10	0.06	0.06	4	0.00	0.00	0	0.00	0.00	0	1.31	0.22	10	0.00	0.00	0	0.00	0.00	0
	**Inner marginal**	**20**			1.13	0.13	0.05	20	0.07	0.06	8	0.09	0.17	5	0.00	0.00	0	0.25	0.17	20	0.02	0.02	6	0.00	0.00	0	0.00	0.00	0	0.57	0.31	20	0.00	0.00	0	0.00	0.00	0
	**Outer marginal**	**18**			1.25	0.13	0.07	18	0.02	0.01	7	0.02	0.01	3	0.00	0.00	0	0.39	0.20	18	0.06	0.02	8	0.00	0.00	0	0.00	0.00	0	0.63	0.36	18	0.00	0.00	0	0.00	0.00	0
	**All teeth**	**64**	2.25	1.96	2.69	0.00	0.00	0	0.03	0.04	20	0.16	0.13	27	0.21	0.26	64	0.55	0.56	54	0.42	0.18	64	0.17	0.18	64	0.00	0.00	0	0.82	0.90	64	0.33	0.09	16	0.00	0.00	0
	**Lateral I**	**20**			1.75	0.00	0.00	0	0.02	0.01	6	0.04	0.03	5	0.14	0.06	20	0.43	0.30	18	0.33	0.18	20	0.13	0.10	20	0.00	0.00	0	0.66	0.50	20	0.00	0.00	0	0.00	0.00	0
Vittina turrita	**Lateral II**	**16**			4.8	0.00	0.00	0	0.06	0.01	7	0.25	0.10	16	0.58	0.26	16	1.04	0.78	16	0.46	0.07	16	0.35	0.26	16	0.00	0.00	0	1.73	1.29	16	0.33	0.09	16	0.00	0.00	0
	**Inner marginal**	**12**			1.33	0.00	0.00	0	0.01	0.00	3	0.01	0.00	2	0.04	0.02	12	0.28	0.22	7	0.51	0.19	12	0.09	0.07	12	0.00	0.00	0	0.39	0.37	12	0.00	0.00	0	0.00	0.00	0
	**Outer marginal**	**16**			1.35	0.00	0.00	0	0.02	0.01	5	0.02	0.01	4	0.06	0.06	16	0.27	0.16	13	0.44	0.20	16	0.09	0.06	16	0.00	0.00	0	0.45	0.28	16	0.00	0.00	0	0.00	0.00	0
	**All teeth**	**58**	1.85	1.66	3.12	0.00	0.00	0	0.98	0.02	34	0.19	0.11	38	0.03	0.01	16	0.62	0.61	36	0.14	0.05	26	0.28	0.20	32	0.00	0.00	0	0.88	1.13	36	0.00	0.00	0	0.00	0.00	0
	**Central**	**18**			2.58	0.00	0.00	0	0.69	0.34	12	0.18	0.06	18	0.03	0.01	6	0.44	0.29	18	0.16	0.03	8	0.18	0.09	18	0.00	0.00	0	0.90	0.99	18	0.00	0.00	0	0.00	0.00	0
Lavigeria grandis	**Lateral**	**14**			4.07	0.00	0.00	0	1.16	0.68	10	0.27	0.09	14	0.03	0.01	9	1.00	0.78	14	0.11	0.06	6	0.41	0.23	14	0.00	0.00	0	1.09	1.39	14	0.00	0.00	0	0.00	0.00	0
	**Marginal I**	**12**			1.34	0.00	0.00	0	1.16	0.48	6	0.02	0.01	3	0.02	0.00	1	0.01		2	0.13	0.07	6	0.00	0.00	0	0.00	0.00	0	0.00	0.00	0	0.00	0.00	0	0.00	0.00	0
	**Marginal II**	**14**			1.39	0.00	0.00	0	1.08	0.29	6	0.02	0.01	3	0.00	0.00	0	0.10	0.01	2	0.14	0.05	7	0.00	0.00	0	0.00	0.00	0	0.05	0.03	4	0.00	0.00	0	0.00	0.00	0
	**All teeth**	**104**	1.79	0.88	2.08	0.00	0.00	0	0.14	0.51	22	0.19	0.06	98	0.03	0.00	2	0.52	0.33	84	0.18	0.08	98	0.18	0.11	83	0.00	0.00	0	0.84	0.47	104	0.00	0.00	0	0.00	0.00	0
	**Central**	**20**			2.56	0.00	0.00	0	0.17	0.01	4	0.17	0.06	20	0.00	0.00	0	0.64	0.31	20	0.17	0.09	18	0.21	0.10	20	0.00	0.00	0	1.20	0.45	20	0.00	0.00	0	0.00	0.00	0
Lavigeria nassa	**Lateral**	**60**			2.13	0.00	0.00	0	0.14	0.06	10	0.22	0.07	56	0.03	0.00	2	0.51	0.33	60	0.17	0.07	56	0.18	0.11	60	0.00	0.00	0	0.88	0.44	60	0.00	0.00	0	0.00	0.00	0
	**Marginal I**	**12**			1.33	0.00	0.00	0	0.14	0.01	4	0.17	0.02	10	0.00	0.00	0	0.22	0.36	3	0.19	0.09	12	0.12	0.13	2	0.00	0.00	0	0.49	0.25	12	0.00	0.00	0	0.00	0.00	0
	**Marginal II**	**12**			0.87	0.00	0.00	0	0.10	0.01	5	0.15	0.04	12	0.00	0.00	0	0.02	0.00	1	0.18	0.07	12	0.01	0.00	1	0.00	0.00	0	0.41	0.16	12	0.00	0.00	0	0.00	0.00	0
	**All teeth**	**60**	0.71	0.63	1.37	0.00	0.00	0	0.22	0.05	32	0.16	0.09	35	0.03	0.02	10	0.17	0.15	23	0.24	0.14	44	0.08	0.07	20	0.08	0.08	24	0.39	0.39	29	0.00	0.00	0	0.00	0.00	0
	**Central**	**14**			0.95	0.00	0.00	0	0.30	0.24	10	0.19	0.10	14	0.02	0.00	2	0.05	0.00	2	0.20	0.10	10	0.02	0.00	2	0.05	0.06	8	0.12	0.06	13	0.00	0.00	0	0.00	0.00	0
Paramelania damoni	**Lateral**	**16**			1.71	0.00	0.00	0	0.22	0.14	8	0.17	0.08	16	0.04	0.01	3	0.19	0.16	16	0.32	0.19	10	0.09	0.08	16	0.06	0.08	8	0.62	0.40	16	0.00	0.00	0	0.00	0.00	0
	**Marginal I**	**18**			0.66	0.00	0.00	0	0.04	0.03	6	0.07	0.05	3	0.01	0.00	2	0.26	0.03	2	0.20	0.13	14	0.01	0.00	1	0.07	0.01	4	0.00	0.00	0	0.00	0.00	0	0.00	0.00	0
	**Marginal II**	**12**			1.04	0.00	0.00	0	0.26	0.09	8	0.10	0.04	2	0.04	0.02	4	0.09	0.09	3	0.23	0.12	10	0.11	0.00	1	0.21	0.02	4	0.00	0.00	0	0.00	0.00	0	0.00	0.00	0

	**All teeth**	**52**	0.80	0.48	1.16	0.00	0.00	0	0.42	0.18	52	0.39	0.24	33	0.00	0.00	0	0.14	0.04	12	0.14	0.04	30	0.05	0.02	18	0.02	0.01	14	0.00	0.00	0	0.00	0.00	0	0.00	0.00	0
	**Central**	**14**			1.33	0.00	0.00	0	0.54	0.30	14	0.49	0.27	11	0.00	0.00	0	0.08	0.01	2	0.14	0.06	8	0.06	0.00	4	0.02	0.01	6	0.00	0.00	0	0.00	0.00	0	0.00	0.00	0
Cleopatra johnstoni	**Lateral**	**16**			1.04	0.00	0.00	0	0.37	0.26	16	0.30	0.23	6	0.00	0.00	0	0.17	0.01	2	0.13	0.04	10	0.05	0.02	8	0.02	0.01	4	0.00	0.00	0	0.00	0.00	0	0.00	0.00	0
	**Marginal I**	**10**			0.83	0.00	0.00	0	0.32	0.16	10	0.25	0.18	5	0.00	0.00	0	0.14	0.04	4	0.10	0.00	2	0.02	0.00	2	0.00	0.00	0	0.00	0.00	0	0.00	0.00	0	0.00	0.00	0
	**Marginal II**	**12**			1.18	0.00	0.00	0	0.42	0.26	12	0.40	0.21	11	0.00	0.00	0	0.16	0.03	4	0.15	0.03	10	0.04	0.01	4	0.01	0.00	4	0.00	0.00	0	0.00	0.00	0	0.00	0.00	0
	**All teeth**	**72**	2.46	0.96	3.32	0.00	0.00	0	0.42	0.26	44	0.17	0.09	58	0.06	0.06	14	0.52	0.33	56	0.38	0.15	70	0.19	0.16	67	0.06	0.01	10	0.87	0.38	70	0.32	0.02	2	0.33	0.17	52
	**Central**	**8**			3.12	0.00	0.00	0	0.58	0.17	6	0.23	0.05	6	0.04	0.00	2	0.34	0.11	8	0.34	0.06	8	0.11	0.04	8	0.07	0.00	2	1.02	0.39	8	0.00	0.00	0	0.39	0.16	8
Reymondia horei	**Lateral**	**44**			3.66	0.00	0.00	0	0.47	0.23	25	0.15	0.09	38	0.07	0.08	8	0.65	0.33	34	0.40	0.17	44	0.24	0.17	44	0.05	0.01	8	0.96	0.39	44	0.32	0.02	2	0.35	0.15	40
	**Marginal I**	**8**			1.78	0.00	0.00	0	0.02	0.01	3	0.18	0.05	4	0.05	0.00	2	0.44	0.30	3	0.43	0.10	6	0.06	0.04	5	0.04	0.01	4	0.54	0.05	6	0.00	0.00	0	0.02	0.01	2
	**Marginal II**	**12**			1.98	0.00	0.00	0	0.34	0.30	10	0.23	0.09	10	0.03	0.00	2	0.25	0.23	11	0.32	0.09	12	0.09	0.03	10	0.05	0.00	2	0.64	0.22	12	0.00	0.00	0	0.03	0.01	2
	**All teeth**	**64**	3.47	1.34	3.62	0.00	0.00	0	0.73	0.26	60	0.18	0.11	64	0.09	0.01	2	0.68	0.47	64	0.24	0.22	58	0.25	0.13	64	0.00	0.00	0	1.45	0.67	64	0.00	0.00	0	0.00	0.00	0
	**Central**	**14**			4.33	0.00	0.00	0	0.94	0.26	12	0.24	0.08	14	0.00	0.00	0	0.79	0.71	14	0.31	0.39	14	0.30	0.20	14	0.00	0.00	0	1.75	1.04	14	0.00	0.00	0	0.00	0.00	0
Spekia zonata	**Lateral**	**12**			3.67	0.00	0.00	0	0.55	0.21	10	0.29	0.08	12	0.00	0.00	0	0.65	0.44	12	0.15	0.07	10	0.29	0.08	12	0.00	0.00	0	1.74	0.45	12	0.00	0.00	0	0.00	0.00	0
	**Marginal I**	**20**			3.4	0.00	0.00	0	0.78	0.30	20	0.14	0.09	20	0.09	0.01	2	0.65	0.33	20	0.23	0.07	20	0.23	0.09	20	0.00	0.00	0	1.28	0.47	20	0.00	0.00	0	0.00	0.00	0
	**Marginal II**	**18**			3.05	0.00	0.00	0	0.63	0.21	18	0.09	0.05	18	0.00	0.00	0	0.66	0.40	18	0.25	0.21	14	0.22	0.09	18	0.00	0.00	0	1.20	0.46	18	0.00	0.00	0	0.00	0.00	0
	**All teeth**	**56**	0.98	0.67	1.37	0.00	0.00	0	0.23	0.10	14	0.12	0.09	48	0.06	0.05	12	0.25	0.16	49	0.15	0.12	15	0.09	0.06	44	0.00	0.00	0	0.47	0.40	56	0.00	0.00	0	0.00	0.00	0
	**Central**	**16**			1.52	0.00	0.00	0	0.16	0.09	5	0.17	0.08	16	0.03	0.02	4	0.22	0.16	16	0.18	0.08	4	0.10	0.07	16	0.00	0.00	0	0.66	0.37	16	0.00	0.00	0	0.00	0.00	0
Faunus ater	**Lateral**	**14**			1.73	0.00	0.00	0	0.08	0.05	5	0.17	0.05	14	0.03	0.01	4	0.36	0.19	14	0.08	0.01	2	0.14	0.05	14	0.00	0.00	0	0.87	0.24	14	0.00	0.00	0	0.00	0.00	0
	**Marginal I**	**14**			1.25	0.00	0.00	0	0.49	0.19	4	0.02	0.01	9	0.15	0.01	2	0.17	0.06	12	0.30	0.05	4	0.03	0.01	7	0.00	0.00	0	0.09	0.10	14	0.00	0.00	0	0.00	0.00	0
	**Marginal II**	**12**			0.78	0.00	0.00	0	0.15	0.01	2	0.04	0.02	9	0.08	0.01	2	0.21	0.08	7	0.03	0.02	5	0.06	0.03	7	0.00	0.00	0	0.21	0.19	12	0.00	0.00	0	0.00	0.00	0
	**All teeth**	**58**	3.97	2.50	4.44	0.28	0.24	44	0.19	0.20	44	0.34	0.29	58	0.83	0.42	56	0.71	0.66	58	0.15	0.10	40	0.03	0.03	2	0.09	0.08	6	1.48	1.07	54	0.34	0.16	46	0.00	0.00	0
Littorina littorea	**Central**	**24**			5.46	0.37	0.25	18	0.19	0.09	22	0.50	0.28	24	1.09	0.25	24	0.92	0.71	24	0.09	0.07	24	0.03	0.03	2	0.00	0.00	0	1.87	1.00	24	0.40	0.13	24	0.00	0.00	0
	**Lateral**	**16**			5.12	0.26	0.22	16	0.26	0.08	16	0.40	0.25	16	0.98	0.23	16	0.79	0.65	16	0.23	0.06	16	0.00	0.00	0	0.13	0.06	4	1.73	0.82	16	0.34	0.16	16	0.00	0.00	0
	**Marginal**	**18**			1.55	0.15	0.21	10	0.02	0.01	6	0.09	0.13	18	0.29	0.24	16	0.36	0.47	18	0.00	0.00	0	0.00	0.00	0	0.01	0.00	2	0.53	0.91	14	0.10	0.04	6	0.00	0.00	0
	**All teeth**	**62**	0.98	0.55	1.34	0.00	0.00	0	0.19	0.28	24	0.29	0.14	58	0.05	0.03	22	0.15	0.11	39	0.11	0.09	28	0.06	0.04	35	0.05	0.01	6	0.44	0.29	62	0.00	0.00	0	0.00	0.00	0
	**Central**	**18**			1.17	0.00	0.00	0	0.16	0.11	12	0.27	0.14	16	0.08	0.01	6	0.09	0.06	18	0.09	0.07	12	0.04	0.03	17	0.00	0.00	0	0.44	0.18	18	0.00	0.00	0	0.00	0.00	0
Paludomus siamensis	**Lateral**	**16**			1.98	0.00	0.00	0	0.35	0.02	2	0.39	0.10	17	0.05	0.02	8	0.21	0.13	16	0.11	0.07	6	0.08	0.04	16	0.00	0.00	0	0.79	0.21	16	0.00	0.00	0	0.00	0.00	0
	**Marginal I**	**12**			1.19	0.00	0.00	0	0.20	0.09	4	0.30	0.10	10	0.03	0.00	2	0.25	0.02	2	0.16	0.16	6	0.00	0.00	0	0.00	0.00	0	0.25	0.15	12	0.00	0.00	0	0.00	0.00	0
	**Marginal II**	**16**			0.84	0.00	0.00	0	0.17	0.08	6	0.21	0.15	16	0.03	0.02	6	0.11	0.08	3	0.09	0.01	4	0.01	0.00	2	0.00	0.00	0	0.22	0.16	16	0.00	0.00	0	0.00	0.00	0

Anentome helena	**All teeth**	**44**	5.68	2.55	6.27	0.00	0.00	0	0.35	0.11	44	0.14	0.05	18	0.07	0.00	2	2.31	1.58	44	0.72	0.51	18	0.89	0.47	44	0.00	0.00	0	1.79	1.39	44	0.00	0.00	0	0.00	0.00	0
	**Central**	**18**			4.99	0.00	0.00	0	0.22	0.25	18	0.12	0.05	14	0.00	0.00	0	2.07	1.70	18	0.82	0.45	10	0.98	0.41	18	0.00	0.00	0	0.78	0.34	18	0.00	0.00	0	0.00	0.00	0
	**Lateral**	**26**			7.06	0.00	0.00	0	0.43	0.25	26	0.19	0.02	4	0.07	0.00	2	2.47	1.51	26	0.60	0.59	8	0.82	0.50	26	0.00	0.00	0	2.48	1.42	26	0.00	0.00	0	0.00	0.00	0
Buccinum undatum	**All teeth**	**54**	5.41	2.10	5.59	0.00	0.00	0	0.24	0.27	54	0.12	0.06	42	0.00	0.00	0	2.11	0.76	54	0.38	0.21	32	0.74	0.23	54	0.00	0.00	0	2.00	1.61	54	0.00	0.00	0	0.00	0.00	0
	**Central**	**16**			5.34	0.00	0.00	0	0.13	0.08	16	0.14	0.05	12	0.00	0.00	0	2.02	0.77	16	0.06	0.03	4	0.67	0.26	16	0.00	0.00	0	2.32	1.60	16	0.00	0.00	0	0.00	0.00	0
	**Lateral**	**38**			5.62	0.00	0.00	0	0.28	0.18	38	0.12	0.06	30	0.00	0.00	0	2.15	0.77	38	0.43	0.18	28	0.77	0.21	38	0.00	0.00	0	1.87	1.61	38	0.00	0.00	0	0.00	0.00	0
Onchidoris bilamellata	**All teeth**	**38**	11.59	9.82	19.59	1.75	0.90	28	0.53	0.17	18	0.99	0.21	24	0.00	0.00	0	5.63	1.59	24	0.24	0.13	22	0.98	0.26	6	0.09	0.08	10	9.38	2.99	24	0.00	0.00	0	0.00	0.00	0
	**Thickened membrane**	**6**			0.62	0.02	0.00	2	0.59	0.49	3	0.00	0.00	0	0.00	0.00	0	0.00	0.00	0	0.01	0.00	2	0.00	0.00	0	0.00	0.00	0	0.00	0.00	0	0.00	0.00	0	0.00	0.00	0
	**Lateral**	**24**			20.04	2.03	0.59	24	0.64	0.54	11	0.99	0.21	24	0.00	0.00	0	5.63	1.59	24	0.28	0.10	18	0.98	0.26	6	0.11	0.08	8	9.38	2.99	24	0.00	0.00	0	0.00	0.00	0
	**Marginal**	**8**			0.26	0.03	0.00	2	0.16	0.07	4	0.00	0.00	0	0.00	0.00	0	0.00	0.00	0	0.06	0.00	2	0.00	0.00	0	0.01	0.00	2	0.00	0.00	0	0.00	0.00	0	0.00	0.00	0
Aeolidia papillosa	**All teeth**	**12**	6.60	3.71	7.4	0.00	0.00	0	0.03	0.49	6	0.16	0.13	8	0.00	0.00	0	1.34	1.11	10	2.06	1.29	10	0.70	0.53	9	0.00	0.00	0	3.11	2.49	12	0.00	0.00	0	0.00	0.00	0
	**Inner part**	**6**			9.78	0.00	0.00	0	0.04	0.04	3	0.25	0.13	4	0.00	0.00	0	2.11	0.65	6	1.23	0.90	6	0.99	0.38	6	0.00	0.00	0	5.16	1.88	6	0.00	0.00	0	0.00	0.00	0
	**Outer part**	**6**			4.78	0.00	0.00	0	0.02	0.01	3	0.08	0.01	4	0.00	0.00	0	0.18	0.09	4	3.32	0.40	4	0.12	0.04	3	0.00	0.00	0	1.06	0.11	6	0.00	0.00	0	0.00	0.00	0
Polycera quadrilineata	**All teeth**	**18**	3.32	1.20	5.89	0.00	0.00	0	0.17	0.03	8	0.58	0.29	18	0.00	0.00	0	2.09	0.35	42	0.68	0.10	8	0.00	0.00	0	0.00	0.00	0	2.37	1.14	18	0.00	0.00	0	0.00	0.00	0
	**Lateral**	**6**			2.37	0.00	0.00	0	0.16	0.05	3	0.70	0.35	6	0.00	0.00	0	0.00	0.00	0	0.61	0.08	4	0.00	0.00	0	0.00	0.00	0	0.90	0.21	6	0.00	0.00	0	0.00	0.00	0
	**Marginal**	**12**			4.55	0.00	0.00	0	0.18	0.09	5	0.52	0.24	12	0.00	0.00	0	0.00	0.00	0	0.75	0.05	4	0.00	0.00	0	0.00	0.00	0	3.10	0.49	12	0.00	0.00	0	0.00	0.00	0
Doris pseudoargus	**All teeth**	**42**	6.54	1.44	8.76	0.74	0.13	42	0.16	0.07	19	0.95	0.23	42	0.00	0.00	0	3.37	1.39	11	0.20	0.05	16	0.00	0.00	0	0.00	0.00	0	3.34	0.99	42	0.00	0.00	0	0.00	0.00	0
	**Outer teeth**	**20**			6.91	0.67	0.13	20	0.13	0.08	10	0.86	0.26	20	0.00	0.00	0	1.95	0.37	20	0.22	0.02	6	0.00	0.00	0	0.00	0.00	0	3.08	0.90	20	0.00	0.00	0	0.00	0.00	0
	**Inner teeth**	**22**			8.02	0.80	0.10	22	0.20	0.07	9	1.04	0.16	22	0.00	0.00	0	2.22	0.28	22	0.18	0.06	10	0.00	0.00	0	0.00	0.00	0	3.58	1.03	22	0.00	0.00	0	0.00	0.00	0
Cornu aspersum	**All teeth**	**40**	2.72	1.20	3.28	0.00	0.00	0	0.00	0.00	0	0.00	0.00	0	0.67	0.18	8	0.00	0.00	0	0.43	0.27	38	0.00	0.00	0	0.00	0.00	0	2.18	0.90	40	0.00	0.00	0	0.00	0.00	0
	**Inner teeth**	**20**			3.32	0.00	0.00	0	0.00	0.00	0	0.00	0.00	0	0.65	0.17	4	0.00	0.00	0	0.40	0.25	18	0.00	0.00	0	0.00	0.00	0	2.27	0.90	20	0.00	0.00	0	0.00	0.00	0
	**Outer teeth**	**20**			3.25	0.00	0.00	0	0.00	0.00	0	0.00	0.00	0	0.70	0.22	4	0.00	0.00	0	0.46	0.30	20	0.00	0.00	0	0.00	0.00	0	2.09	0.92	20	0.00	0.00	0	0.00	0.00	0

Supplementary Table 4. For all species and all teeth: correlations, estimated by row-wise method, between the individual elements studied, all elements, tooth row quantity, radular width, radular area, and radular length are given.

	All elements	Tooth rows	Radular width	Ca	Cl	Cu	F	Fe	Radular area	K	Radular length	Mg	Na	P	S	Si
All elements	1.0000	0.0042	-0.0331	0.6451	0.7671	0.3003	0.3680	0.8427	0.1302	0.6995	0.3190	0.6501	0.3467	0.7611	0.5572	0.9534
Tooth rows	-	1.0000	0.0832	-0.2900	-0.3919	0.4192	-0.4058	-0.4553	0.3600	0.1808	0.7385	-0.1918	-0.1604	-0.2546	0.0581	0.1766
Radular width	-	-	1.0000	-0.0651	-0.0080	-0.1356	-0.2420	0.1659	0.8090	0.5003	0.2490	0.0773	-0.1828	-0.0488	-0.0102	-0.0423
Ca	-	-	-	1.0000	0.5372	0.2259	0.7680	0.6199	-0.1078	0.3222	-0.1002	0.5331	0.3094	0.7655	0.2573	0.6276
Cl	-	-	-	-	1.0000	0.0572	0.4111	0.0440	-0.0661	0.6914	-0.2076	0.0224	0.0284	0.8127	0.2263	0.0458
Cu	-	-	-	-	-	1.0000	0.0000	-1.0000	0.0773	-0.1058	0.2841	0.2279	0.4248	0.0735	-0.1087	-0.1462
F	-	-	-	-	-	-	1.0000	0.4774	-0.3801	-0.3748	-0.3367	0.4982	0.2849	0.6402	-0.0357	0.3634
Fe	-	-	-	-	-	-	-	1.0000	0.0972	0.2475	-0.1050	0.6293	0.5177	0.6084	0.3979	0.8123
Radular area	-	-	-	-	-	-	-	-	1.0000	0.6269	0.6560	-0.0243	-0.2363	-0.0210	0.2309	0.3747
K	-	-	-	-	-	-	-	-	-	1.0000	0.5732	0.5243	0.4182	0.6121	0.8370	0.6341
Radular length	-	-	-	-	-	-	-	-	-	-	1.0000	0.0254	-0.2097	0.0687	0.3758	0.6224
Mg	-	-	-	-	-	-	-	-	-	-	-	1.0000	0.4927	0.5588	0.2312	0.7014
Na	-	-	-	-	-	-	-	-	-	-	-	-	1.0000	0.3062	-0.1934	-0.1510
P	-	-	-	-	-	-	-	-	-	-	-	-	-	1.0000	0.4598	0.7559
S	-	-	-	-	-	-	-	-	-	-	-	-	-	-	1.0000	0.8313
Si	-	-	-	-	-	-	-	-	-	-	-	-	-	-	-	1.0000

Supplementary Table 5. For all species on soft ingesta: correlations, estimated by row-wise method, between the individual elements studied, all elements, tooth row quantity, radular width, radular area, and radular length are given.

	Na	Mg	P	S	K	Ca	All elements	Radular length	Radular width	Radular area	Tooth rows	Cl
Na	1.0000	0.9798	-0.4860	-0.4031	-0.0090	0.1967	-0.2035	0.4334	-0.4290	0.4322	0.4329	-0.4371
Mg	-	1.0000	-0.0681	-0.1788	0.0911	0.3953	-0.0633	0.3803	-0.3768	0.3801	0.3788	-0.2833
P	-	-	1.0000	0.1701	1.0000	0.7373	0.9275	-0.6417	0.6368	-0.6383	-0.6415	0.7072
S	-	-	-	1.0000	-0.1051	-0.8910	0.5402	-0.8049	0.8026	-0.7986	-0.8050	0.1175
K	-	-	-	-	1.0000	0.0000	-0.0277	0.0181	0.0181	0.0181	-0.0181	0.8902
Ca	-	-	-	-	-	1.0000	0.9000	0.0563	0.0563	0.0563	0.0000	0.9976
All elements	-	-	-	-	-	-	1.0000	-0.8165	0.8162	-0.8080	-0.8172	0.9323
Radular length	-	-	-	-	-	-	-	1.0000	-0.9942	0.9948	0.9994	-0.7278
Radular width	-	-	-	-	-	-	-	-	1.0000	-0.9780	-0.9963	0.7375
Radular area	-	-	-	-	-	-	-	-	-	1.0000	0.9915	-0.7118
Tooth rows	-	-	-	-	-	-	-	-	-	-	1.0000	-0.7292
Cl	-	-	-	-	-	-	-	-	-	-	-	1.0000

Supplementary Table 6. For all species on soft-to-solid ingesta: correlations, estimated by row-wise method, between the individual elements studied, all elements, tooth row quantity, radular width, radular area, and radular length are given.

	Na	Mg	Si	P	S	K	Ca	All elements	Radular length	Radular width	Radular area	Tooth rows	Cl	Cu
Na	1.0000	0.0291	-0.5241	0.3604	0.0764	-0.4508	0.1070	0.3204	-0.0264	-0.1864	-0.0848	0.0995	0.4543	-0.0786
Mg	-	1.0000	0.2078	0.1038	-0.0720	0.3055	0.1166	0.1861	-0.1920	-0.0277	-0.1214	-0.1452	-0.0336	0.3808
Si	-	-	1.0000	0.2245	0.3300	1.0000	0.1705	0.2664	-0.0298	0.5044	0.4553	0.0998	-0.1222	-0.0510

P	-	-	-	1.0000	0.1555	0.7421	0.4183	0.8657	-0.1311	0.1703	0.1630	-0.4524	0.8199	0.4563
S	-	-	-	-	1.0000	0.8022	0.1219	0.3160	-0.0504	0.0989	0.0947	-0.0595	0.2826	-0.3042
K	-	-	-	-	-	1.0000	0.7695	0.2684	-0.0334	-0.0334	-0.0334	-0.0334	0.9417	0.0000
Ca	-	-	-	-	-	-	1.0000	0.7410	0.0232	0.2909	0.3081	-0.0204	0.4731	0.2579
All elements	-	-	-	-	-	-	-	1.0000	-0.0572	0.0954	0.1157	-0.4332	0.8246	0.4445
Radular length	-	-	-	-	-	-	-	-	1.0000	0.4330	0.6219	0.5617	-0.1173	-0.0960
Radular width	-	-	-	-	-	-	-	-	-	1.0000	0.9671	0.3174	-0.0220	-0.0962
Radular area	-	-	-	-	-	-	-	-	-	-	1.0000	0.4168	0.0662	-0.0960
Tooth rows	-	-	-	-	-	-	-	-	-	-	-	1.0000	-0.3108	-0.0955
Cl	-	-	-	-	-	-	-	-	-	-	-	-	1.0000	0.0595
Cu	-	-	-	-	-	-	-	-	-	-	-	-	-	1.0000

Supplementary Table 7. For all species on medium ingesta: correlations, estimated by row-wise method, between the individual elements studied, all elements, tooth row quantity, radular width, radular area, and radular length are given.

	Na	Mg	Si	P	S	K	Ca	All elements	Radular length	Radular width	Radular area	Tooth rows	F	Cl
Na	1.0000	0.2541	0.3488	0.5705	0.2236	-0.0219	0.4870	0.4959	-0.3992	-0.5271	-0.4178	-0.3363	0.4883	1.0000
Mg	-	1.0000	0.3773	0.6078	0.2734	0.7441	0.5527	0.5720	-0.8085	-0.5184	-0.7978	-0.7495	0.6386	-0.8933
Si	-	-	1.0000	0.0483	0.8141	0.0000	0.1209	0.6059	0.0338	0.0338	0.0338	0.0338	-0.0777	0.0000
P	-	-	-	1.0000	0.6382	0.2912	0.9598	0.9835	-0.7252	-0.9242	-0.7522	-0.6764	0.9801	-0.8459
S	-	-	-	-	1.0000	0.4092	0.2181	0.2299	-0.5842	-0.6071	-0.5950	-0.6643	0.6528	0.9618
K	-	-	-	-	-	1.0000	0.3183	0.6218	0.0557	0.0557	0.0557	0.0000	0.3435	0.0000
Ca	-	-	-	-	-	-	1.0000	0.9828	-0.6056	-0.7238	-0.6247	-0.5236	0.9439	-0.7775
All elements	-	-	-	-	-	-	-	1.0000	-0.4865	-0.5094	-0.4956	-0.4246	0.9717	-0.8207
Radular length	-	-	-	-	-	-	-	-	1.0000	0.8291	0.9985	0.9898	-0.6890	0.1998
Radular width	-	-	-	-	-	-	-	-	-	1.0000	0.8576	0.7978	-0.8305	0.1998
Radular area	-	-	-	-	-	-	-	-	-	-	1.0000	0.9861	-0.7114	0.1998
Tooth rows	-	-	-	-	-	-	-	-	-	-	-	1.0000	-0.6476	0.0000
F	-	-	-	-	-	-	-	-	-	-	-	-	1.0000	-0.7820
Cl	-	-	-	-	-	-	-	-	-	-	-	-	-	1.0000

Supplementary Table 8. For all species on medium-to-solid ingesta: correlations, estimated by row-wise method, between the individual elements studied, all elements, tooth row quantity, radular width, radular area, and radular length are given.

	Na	Mg	Si	P	S	K	Ca	Fe	All elements	Radular length	Radular width	Radular area	Tooth rows	F	Cl
Na	1.0000	0.3027	0.7349	0.1349	-0.4718	0.9918	0.3204	0.1346	0.5016	0.6371	-0.6366	-0.6363	0.6374	-0.1543	-0.1472
Mg	-	1.0000	0.6193	0.1632	-0.5287	0.1185	0.2258	0.3255	0.4480	0.3305	-0.3336	-0.3350	0.3271	0.0862	0.1535
Si	-	-	1.0000	0.3542	-0.5770	0.9451	0.5224	0.2057	0.6983	0.6726	-0.6721	-0.6720	0.6735	0.2828	0.3018
P	-	-	-	1.0000	-0.1127	0.3511	0.9374	0.2233	0.8971	0.1314	-0.1319	-0.1319	0.1293	1.0000	0.9983
S	-	-	-	-	1.0000	-0.6417	-0.2580	-0.1534	-0.3129	-0.6663	0.6665	0.6666	-0.6659	0.0010	0.1154
K	-	-	-	-	-	1.0000	0.9194	-0.2280	0.8096	0.0600	0.0600	0.0600	0.0600	0.9191	0.0000
Ca	-	-	-	-	-	-	1.0000	0.3338	0.9579	0.3182	-0.3181	-0.3178	0.3166	0.9285	0.9808

Fe	-	-	-	-	-	-	-	1.0000	0.4299	0.0285	-0.0277	-0.0273	0.0294	0.1946	0.0428
All elements	-	-	-	-	-	-	-	-	1.0000	0.3627	-0.3631	-0.3631	0.3611	0.8990	0.9287
Radular length	-	-	-	-	-	-	-	-	-	1.0000	-1.0000	-0.9999	0.9999	0.0468	-0.1321
Radular width	-	-	-	-	-	-	-	-	-	-	1.0000	1.0000	-0.9998	0.0468	0.1294
Radular area	-	-	-	-	-	-	-	-	-	-	-	1.0000	-0.9998	0.0468	0.1296
Tooth rows	-	-	-	-	-	-	-	-	-	-	-	-	1.0000	0.0468	-0.1439
F	-	-	-	-	-	-	-	-	-	-	-	-	-	1.0000	0.0000
Cl	-	-	-	-	-	-	-	-	-	-	-	-	-	-	1.0000

Supplementary Table 9. For all species on solid ingesta: correlations, estimated by row-wise method, between the individual elements studied, all elements, tooth row quantity, radular width, radular area, and radular length are given.

	Na	Mg	Si	P	S	K	Ca	Fe	All elements	Radular length	Radular width	Radular area	Tooth rows	F	Cl	Cu
Na	1.0000	0.5367	-0.4898	0.3931	-0.2995	0.2752	0.5010	0.1631	0.2902	-0.3326	-0.1370	-0.3181	-0.5428	0.3534	0.2253	0.2403
Mg	-	1.0000	0.7626	0.6259	0.3225	0.3859	0.6228	0.1671	0.6753	0.1357	0.2484	0.1601	-0.2904	0.6340	-0.0645	-0.0804
Si	-	-	1.0000	0.7501	0.8222	0.3729	0.6846	0.2117	0.9438	0.7329	0.3918	0.7314	0.5604	0.4421	-0.1907	0.3519
P	-	-	-	1.0000	0.7079	0.8128	0.7747	-0.0042	0.8697	0.4610	0.1556	0.4596	0.0679	0.2741	0.7388	0.1720
S	-	-	-	-	1.0000	0.7830	0.5768	-0.1478	0.7455	0.7887	0.3785	0.7815	0.5065	-0.0351	0.3868	-0.0610
K	-	-	-	-	-	1.0000	0.6127	0.0357	0.6105	0.5082	0.2352	0.5076	0.1539	0.1413	-0.3759	-0.1058
Ca	-	-	-	-	-	-	1.0000	0.3896	0.7187	0.0703	-0.2456	0.0634	-0.3322	0.3093	0.4794	0.2163
Fe	-	-	-	-	-	-	-	1.0000	0.7597	0.1717	0.2570	0.1788	0.1258	0.3456	-1.0000	-1.0000
All elements	-	-	-	-	-	-	-	-	1.0000	0.4962	0.1651	0.4917	0.1477	0.3727	0.6909	0.3738
Radular length	-	-	-	-	-	-	-	-	-	1.0000	0.5327	0.9952	0.6529	-0.0751	-0.0125	0.1754
Radular width	-	-	-	-	-	-	-	-	-	-	1.0000	0.6015	0.5212	-0.5965	0.0636	0.1754
Radular area	-	-	-	-	-	-	-	-	-	-	-	1.0000	0.6381	-0.0876	0.0172	0.1754
Tooth rows	-	-	-	-	-	-	-	-	-	-	-	-	1.0000	-0.2955	-0.0730	0.1754
F	-	-	-	-	-	-	-	-	-	-	-	-	-	1.0000	0.0000	0.0000
Cl	-	-	-	-	-	-	-	-	-	-	-	-	-	-	1.0000	0.2635
Cu	-	-	-	-	-	-	-	-	-	-	-	-	-	-	-	1.0000

Supplementary Table 10. For all Caenogastropoda: correlations, estimated by row-wise method, between the individual elements studied, all elements, tooth row quantity, radular width, radular area, and radular length are given.

	Na	Mg	Si	P	S	K	Ca	Fe	All elements	Radular length	Radular width	Radular area	Tooth rows	F	Cl	Cu
Na	1.0000	0.0805	-0.4220	-0.0766	-0.1055	0.1726	0.0645	0.0215	0.0997	0.1623	0.0599	0.0872	0.1112	0.1543	0.0797	0.2403
Mg	-	1.0000	0.4899	-0.1037	-0.1994	0.0563	0.1003	0.3535	0.1222	0.1844	-0.2272	-0.0630	0.2014	0.0862	-0.2294	-0.0804
Si	-	-	1.0000	0.2624	-0.2031	0.8765	0.4769	0.2886	0.6648	0.7703	-0.5443	0.5207	0.7512	0.2828	-0.1831	0.3519
P	-	-	-	1.0000	0.3275	0.2708	0.4844	0.2702	0.8297	-0.0983	0.2607	0.2008	-0.3480	1.0000	0.9210	0.1720
S	-	-	-	-	1.0000	0.5901	0.1371	-0.1060	0.3456	-0.1026	0.1762	0.1067	-0.1173	0.0010	0.4418	-0.0610
K	-	-	-	-	-	1.0000	0.6094	-0.2280	0.2635	0.1229	-0.0003	0.0805	0.1810	0.9191	0.6914	-0.1058
Ca	-	-	-	-	-	-	1.0000	0.4187	0.8342	0.0686	0.1714	0.1889	-0.0896	0.9285	0.4459	0.2163
Fe	-	-	-	-	-	-	-	1.0000	0.5118	0.0248	-0.0219	0.0520	0.0266	0.1946	-0.3531	-1.0000

All elements	-	-	-	-	-	-	-	-	1.0000	0.2104	0.2559	0.3376	0.0121	0.8990	0.8440	0.3738
Radular length	-	-	-	-	-	-	-	-	-	1.0000	0.1578	0.6709	0.8381	0.0468	-0.1323	0.1754
Radular width	-	-	-	-	-	-	-	-	-	-	1.0000	0.8304	-0.2179	0.0468	0.2514	0.1754
Radular area	-	-	-	-	-	-	-	-	-	-	-	1.0000	0.2844	0.0468	0.2171	0.1754
Tooth rows	-	-	-	-	-	-	-	-	-	-	-	-	1.0000	0.0468	-0.5491	0.1754
F	-	-	-	-	-	-	-	-	-	-	-	-	-	1.0000	0.0000	0.0000
Cl	-	-	-	-	-	-	-	-	-	-	-	-	-	-	1.0000	0.2635
Cu	-	-	-	-	-	-	-	-	-	-	-	-	-	-	-	1.0000

Supplementary Table 11. For all Cephalopoda: correlations, estimated by row-wise method, between the individual elements studied, all elements, tooth row quantity, radular width, radular area, and radular length are given.

	Na	Mg	Si	P	S	Ca	All elements	Radular length	Radular width	Radular area	Tooth rows	Cl	Cu
Na	1.0000	-0.0862	0.4344	0.1609	0.2682	-0.3012	-0.2053	-0.0924	-0.0934	-0.0922	-0.0869	-0.0759	-0.0786
Mg	-	1.0000	0.2170	0.4344	-0.3656	0.3113	0.5072	-0.0615	-0.0617	-0.0615	-0.0597	0.0676	0.3808
Si	-	-	1.0000	0.0629	-0.1471	-0.1852	-0.0197	-0.1952	-0.1955	-0.1950	-0.1957	-0.1474	-0.0510
P	-	-	-	1.0000	-0.1702	0.2442	0.6100	0.0236	0.0231	0.0237	0.0268	0.1289	0.4563
S	-	-	-	-	1.0000	-0.2801	-0.2726	-0.0501	-0.0505	-0.0501	-0.0466	-0.0250	-0.3042
Ca	-	-	-	-	-	1.0000	0.8964	-0.1890	-0.1895	-0.1890	-0.1844	0.6448	0.2579
All elements	-	-	-	-	-	-	1.0000	-0.2004	-0.2010	-0.2004	-0.1961	0.6322	0.4445
Radular length	-	-	-	-	-	-	-	1.0000	1.0000	1.0000	0.9981	-0.3743	-0.0960
Radular width	-	-	-	-	-	-	-	-	1.0000	0.9999	0.9974	-0.3741	-0.0962
Radular area	-	-	-	-	-	-	-	-	-	1.0000	0.9981	-0.3743	-0.0960
Tooth rows	-	-	-	-	-	-	-	-	-	-	1.0000	-0.3757	-0.0955
Cl	-	-	-	-	-	-	-	-	-	-	-	1.0000	0.0595
Cu	-	-	-	-	-	-	-	-	-	-	-	-	1.0000

Supplementary Table 12. For all Heterobranchia: correlations, estimated by row-wise method, between the individual elements studied, all elements, tooth row quantity, radular width, radular area, and radular length are given.

	Na	Mg	Si	P	S	K	Ca	All elements	Radular length	Radular width	Radular area	Tooth rows	F	Cl
Na	1.0000	0.1215	0.0000	0.4554	-0.3232	-0.0219	0.3690	0.3796	0.0304	-0.2712	-0.2575	0.4494	0.3003	0.7044
Mg	-	1.0000	0.0000	0.3661	-0.5303	0.7441	0.3385	0.3943	0.5955	0.3386	0.3569	0.6736	0.1975	0.1731
Si	-	-	1.0000	0.0000	0.6467	0.0000	0.0449	0.8710	0.1121	0.1121	0.1121	0.1121	0.0000	0.0000
P	-	-	-	1.0000	-0.3555	0.2912	0.9311	0.9714	-0.0716	-0.5598	-0.5397	0.6801	0.9868	0.0900
S	-	-	-	-	1.0000	0.4092	-0.2201	-0.0802	-0.1867	-0.2883	-0.1836	-0.1950	0.1923	-0.8609
K	-	-	-	-	-	1.0000	0.3183	0.6218	0.0557	0.0557	0.0557	0.0000	0.3435	0.0000
Ca	-	-	-	-	-	-	1.0000	0.9688	-0.3715	-0.5087	-0.4316	-0.3027	0.9056	0.5645
All elements	-	-	-	-	-	-	-	1.0000	-0.3709	-0.3782	-0.3967	-0.3234	0.9651	0.4955
Radular length	-	-	-	-	-	-	-	-	1.0000	0.7151	0.9872	0.9936	-0.6198	0.3213
Radular width	-	-	-	-	-	-	-	-	-	1.0000	0.8135	0.6654	-0.6536	0.3502
Radular area	-	-	-	-	-	-	-	-	-	-	1.0000	0.9705	-0.6506	0.3288

Tooth rows	-	-	-	-	-	-	-	-	-	-	-	1.0000	0.6551	0.3175
F	-	-	-	-	-	-	-	-	-	-	-	-	1.0000	-0.7820
Cl	-	-	-	-	-	-	-	-	-	-	-	-	-	1.0000

Supplementary Table 13. For all Neritimorpha: correlations, estimated by row-wise method, between the individual elements studied, all elements, tooth row quantity, radular width, radular area, and radular length are given.

	Na	Mg	Si	P	S	Ca	Fe	All elements	Radular length	Radular width	Radular area	Tooth rows	Cl
Na	1.0000	-0.7308	0.6329	0.3771	0.0118	0.4393	-0.5176	0.5869	0.2285	-0.2285	-0.2285	-0.2285	0.4055
Mg	-	1.0000	0.7413	0.5268	0.1953	0.5420	-0.0276	0.6767	0.0111	-0.0111	-0.0111	-0.0111	0.5420
Si	-	-	1.0000	0.4296	0.0406	0.4616	-0.4164	0.6298	-0.1152	0.1152	0.1152	0.1152	0.4549
P	-	-	-	1.0000	0.0525	0.9961	0.0972	0.9704	0.2946	-0.2946	-0.2946	-0.2946	1.0000
S	-	-	-	-	1.0000	0.0721	0.4442	0.1642	-0.0304	0.0304	0.0304	0.0304	0.0800
Ca	-	-	-	-	-	1.0000	0.0972	0.9687	0.2851	-0.2851	-0.2851	-0.2851	0.9947
Fe	-	-	-	-	-	-	1.0000	0.0971	0.0071	-0.0071	-0.0071	-0.0071	0.0972
All elements	-	-	-	-	-	-	-	1.0000	0.2075	-0.2075	-0.2075	-0.2075	0.9666
Radular length	-	-	-	-	-	-	-	-	1.0000	-1.0000	-1.0000	-1.0000	0.2655
Radular width	-	-	-	-	-	-	-	-	-	1.0000	1.0000	1.0000	-0.2655
Radular area	-	-	-	-	-	-	-	-	-	-	1.0000	1.0000	-0.2655
Tooth rows	-	-	-	-	-	-	-	-	-	-	-	1.0000	-0.2655
Cl	-	-	-	-	-	-	-	-	-	-	-	-	1.0000

Supplementary Table 14. For all Patellogastropoda: correlations, estimated by row-wise method, between the individual elements studied, all elements, tooth row quantity, radular width, radular area, and radular length are given.

	Na	Mg	Si	P	S	K	Ca	Fe	All elements	Radular length	Radular width	Radular area	Tooth rows	F
Na	1.0000	0.4054	0.0444	0.1720	0.0004	0.7917	-0.0573	-0.2786	0.0398	0.0292	0.0292	0.0292	0.0292	0.1990
Mg	-	1.0000	0.6905	0.7199	0.5765	0.2765	0.7898	-0.3393	0.8208	-0.0603	-0.0603	-0.0603	-0.0603	0.3684
Si	-	-	1.0000	0.6218	0.5127	0.1917	0.6912	-0.3365	0.9518	-0.0187	-0.0187	-0.0187	-0.0187	0.3986
P	-	-	-	1.0000	0.7770	0.8953	0.7553	-0.2064	0.8182	0.0251	0.0251	0.0251	0.0251	0.4228
S	-	-	-	-	1.0000	0.7859	0.6292	-0.0483	0.7286	0.0597	0.0597	0.0597	0.0597	0.0943
K	-	-	-	-	-	1.0000	0.5936	-0.0835	0.7368	0.0578	0.0578	0.0578	0.0578	0.2155
Ca	-	-	-	-	-	-	1.0000	-0.2542	0.8481	0.0248	0.0248	0.0248	0.0248	0.2714
Fe	-	-	-	-	-	-	-	1.0000	0.0069	0.0685	0.0685	0.0685	0.0685	-0.0058
All elements	-	-	-	-	-	-	-	-	1.0000	0.0037	0.0037	0.0037	0.0037	0.4760
Radular length	-	-	-	-	-	-	-	-	-	1.0000	1.0000	1.0000	1.0000	-0.1141
Radular width	-	-	-	-	-	-	-	-	-	-	1.0000	1.0000	1.0000	-0.1141
Radular area	-	-	-	-	-	-	-	-	-	-	-	1.0000	1.0000	-0.1141
Tooth rows	-	-	-	-	-	-	-	-	-	-	-	-	1.0000	-0.1141
F	-	-	-	-	-	-	-	-	-	-	-	-	-	1.0000

Supplementary Table 15. For all Polyplacophora: correlations, estimated by row-wise method, between the individual elements studied, all elements, tooth row quantity, radular width, radular area, and radular length are given.

	Na	Mg	Si	P	S	K	Ca	Fe	All elements	Radular length	Radular width	Radular area	Tooth rows	F	Cl
Na	1.0000	0.3369	0.0000	0.8285	-0.0401	0.5759	0.5620	0.1486	0.6320	-0.2192	-0.2162	-0.2156	-0.2120	-0.1266	1.0000
Mg	-	1.0000	0.0000	0.5393	0.7081	0.7346	0.4142	-0.1873	0.6600	0.2766	0.2773	0.2777	0.2789	0.4118	0.0000
Si	-	-	1.0000	0.0000	0.0000	0.0000	0.4262	0.9995	0.5247	0.3227	0.3227	0.3227	0.0000	0.0000	0.0000
P	-	-	-	1.0000	-0.2399	0.9305	0.6599	0.0459	0.7088	-0.1558	-0.1549	-0.1546	-0.1531	0.0171	0.0000
S	-	-	-	-	1.0000	0.5752	-0.1577	-0.0225	0.2565	0.3810	0.3814	0.3815	0.3820	0.2529	0.0000
K	-	-	-	-	-	1.0000	-0.0454	-0.1597	-0.0641	0.2489	0.2514	0.2518	0.2542	0.9045	0.0000
Ca	-	-	-	-	-	-	1.0000	0.4868	0.6421	-0.2840	-0.2835	-0.2834	-0.2828	0.2319	0.0000
Fe	-	-	-	-	-	-	-	1.0000	0.9650	0.2607	0.2613	0.2615	0.2621	0.3292	0.0000
All elements	-	-	-	-	-	-	-	-	1.0000	0.2032	0.2035	0.2036	0.2039	0.4926	1.0000
Radular length	-	-	-	-	-	-	-	-	-	1.0000	0.9999	0.9998	0.9994	0.0767	-1.0000
Radular width	-	-	-	-	-	-	-	-	-	-	1.0000	1.0000	0.9998	0.0767	-1.0000
Radular area	-	-	-	-	-	-	-	-	-	-	-	1.0000	0.9998	0.0767	-1.0000
Tooth rows	-	-	-	-	-	-	-	-	-	-	-	-	1.0000	0.0767	-1.0000
F	-	-	-	-	-	-	-	-	-	-	-	-	-	1.0000	0.0000
Cl	-	-	-	-	-	-	-	-	-	-	-	-	-	-	1.0000

Supplementary Table 16. For all Vetigastropoda: correlations, estimated by row-wise method, between the individual elements studied, all elements, tooth row quantity, radular width, radular area, and radular length are given.

	Na	Mg	Si	P	S	Ca	All elements	Radular length	Radular width	Radular area	Tooth rows	F
Na	1.0000	0.4673	0.3488	-0.1358	0.5186	0.0360	0.3265	-0.7380	-0.7360	-0.7351	-0.7272	0.3462
Mg	-	1.0000	0.0077	-0.1920	0.2969	0.0618	0.0964	-0.6092	-0.6085	-0.6082	-0.6043	0.1491
Si	-	-	1.0000	0.0614	-0.4365	0.3198	0.6584	0.4389	0.4392	0.4393	0.4385	-0.1941
P	-	-	-	1.0000	-0.0998	0.3308	0.6309	0.0617	0.0620	0.0622	0.0669	0.2921
S	-	-	-	-	1.0000	0.0507	0.1816	-0.4564	-0.4563	-0.4562	-0.4481	-0.0577
Ca	-	-	-	-	-	1.0000	0.8458	0.0400	0.0408	0.0411	0.0407	0.2588
All elements	-	-	-	-	-	-	1.0000	-0.0594	-0.0587	-0.0583	-0.0544	0.3530
Radular length	-	-	-	-	-	-	-	1.0000	0.9999	0.9999	0.9973	-0.2318
Radular width	-	-	-	-	-	-	-	-	1.0000	1.0000	0.9977	-0.2312
Radular area	-	-	-	-	-	-	-	-	-	1.0000	0.9978	-0.2308
Tooth rows	-	-	-	-	-	-	-	-	-	-	1.0000	-0.2173
F	-	-	-	-	-	-	-	-	-	-	-	1.0000

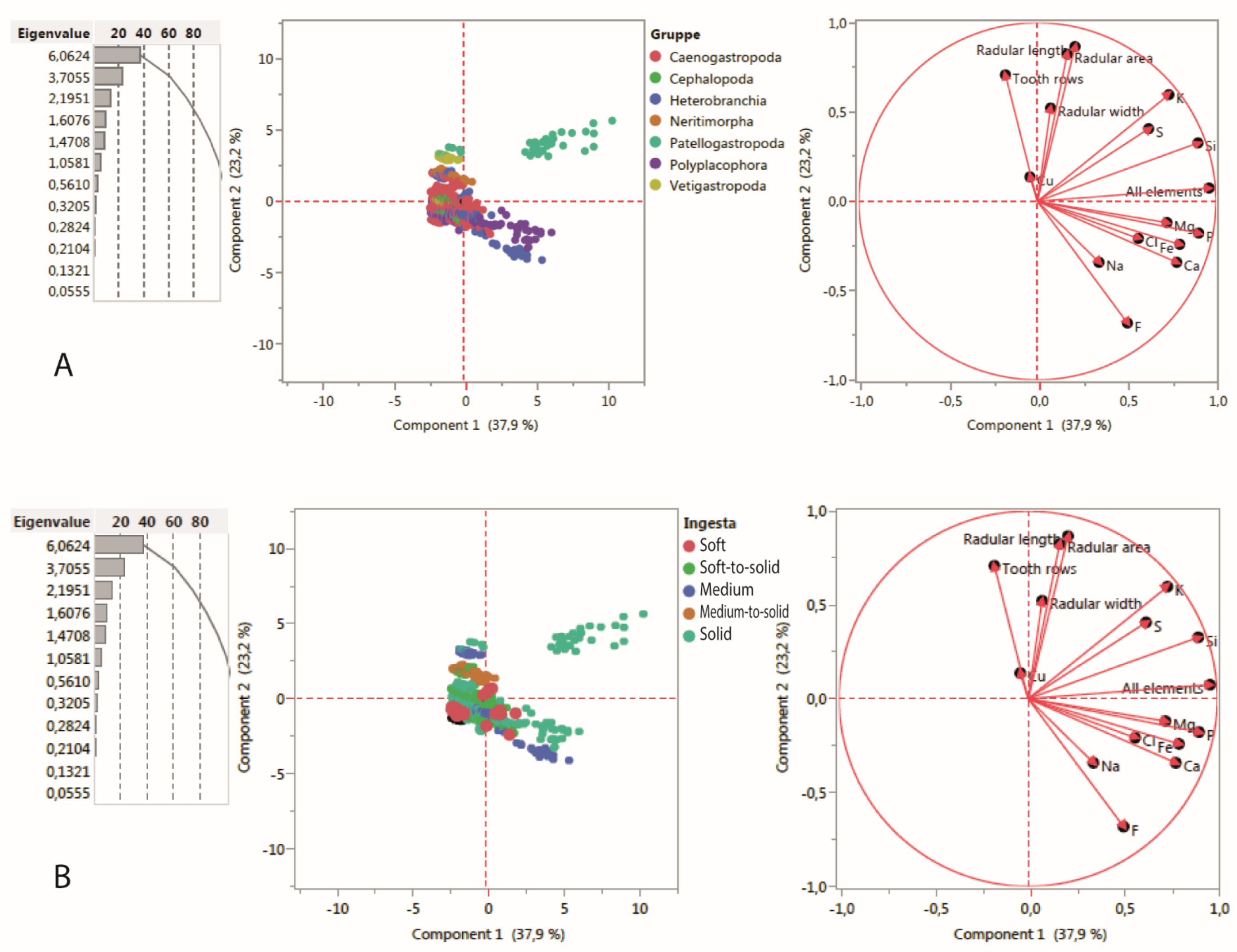

Supplementary Figure 31. PCA of all species pooled together with A. highlighted taxonomic groups and B. highlighted ingesta categories. No clustering can be detected.

Supplementary Table 17. Summary of the literature on radular chemistry sorted by phylogeny. Methodology of elemental analyses, the analysed zone of the radula and the tooth type are listed.

Taxa		Family	Species	Reference	Method	Radular zone	Tooth type and part	Comment	Elements										
									F	Na	Mg	Si	P	S	Cl	K	Ca	Fe	Cu
Aplacophora	Chaetodermatida	Chaetodermatidae	*Falcidens spec.*	(45)	EDXA, ESI	Not defined	Axial plate, denticles					x	x	x			x	x	x
		Prochaetodermatidae	*Chevroderma turnerae*	(44)	X-ray diffraction, X-ray microanalysis	Not defined	Not defined				x		x				x	x	
Gastropoda	Caenogastropoda	Charoniidae	*Charonia lampas*	Bergh from 'Konigl. Danske Videnskabernes Selkabs Skrifur' 5th Raekke, 3 Bind; translation in (41) (termed by Bergh *Buccinum antiquorum* and by (41) *Triton nodiferum*)	Acid treatments and boiling	Whole radula	Not defined, but probably all						x				x	x	
		Strombidae	*Gibberulus gibberulus*	Bergh from 'Konigl. Danske Videnskabernes Selkabs Skrifur,' 5th Raekke, 3 Bind; translation in (41) (termed *Strombus gibberulus*)	Acid treatments and boiling	Whole radula	Not defined, but probably all		?	?	?	?	?	?	?	?	?	?	?
		Tateidae	*Potamopyrgus antipodarum*	(43; termed *Hydrobia jenkinsi*)	Ashing and acid treatment	Not defined, but probably whole radula	Not defined, but probably all					-						-	
		Aporrhaidae	*Aporrhais pespelecani*	(43; termed *Hydrobia jenkinsi*)	Ashing and acid treatment	Not defined, but probably whole radula	Not defined, but probably all					-						-	
		Littorinidae	*Littorina littorea*	(42)	Ashing and acid treatment	Whole radula	All				x		x				x	x	
				(43)	Ashing and acid treatment	Not defined, but probably whole radula	Not defined, but probably all					-						-	
			Lacuna vincta	(43)	Ashing and acid treatment	Not defined, but probably whole radula	Not defined, but probably all					-						-	
		Velutinidae	*Lamellaria perspicua*	Bergh from 'Konigl. Danske Videnskabernes Selkabs Skrifur,' 5th Raekke, 3 Bind; translation in (41) (termed *Marsenia perspicua*)	Acid treatments and boiling	Whole radula	Not defined, but probably all					-							
		Tonnidea	*Tonna galea*	(41; termed *Dolium galea*)	Ashing and acid treatment	Whole radula	All						x				x	x	
		Muricidae	*? Termed Murex branchialis*	(43)	Ashing and acid treatment	Not defined, but probably whole radula	Not defined, but probably all					-						-	
			Nucella lapillus	(42)	Not defined	Whole radula	All						x ?				x ?	x ?	
				(43)	Ashing and acid treatment	Not defined, but probably whole radula	Not defined, but probably all					-						-	
		Buccinidae	*Buccinum undatum*	(42)	Not defined	Whole radula	All						x ?				x ?	x ?	
				(43)	Ashing and acid treatment	Not defined, but probably whole radula	Not defined, but probably all					-						-	
	Heterobranchia	Helicidae	*Cornu aspersum*	(42; termed *Helix aspersa*)	Ashing and acid treatment	Whole radula	All	spring					x						
								winter									x		
				(116)	EDX	Working zone	Central and lateral teeth					x					x		
			Helix nemoralis	(41)	Ashing and acid treatment	Whole radula	All						x				x	x	
		Scaphandridae	*Scaphander lignarius*	(43)	Ashing and acid treatment	Not defined, but probably whole radula	Not defined, but probably all					-						-	
		Aplysiidae	*Aplysia punctata*	(43)	Ashing and acid treatment	Not defined, but probably whole radula	Not defined, but probably all					-						-	
		Discodorididae	*Jorunna tomentosa*	(43)	Ashing and acid treatment	Not defined, but probably whole radula	Not defined, but probably all					-						-	
	Vetigastropoda	Fissurellidae	*Megathura crenulata*	(117)	EDX	Working zone	All			x	x	x			x		x	x	

				(118)	Inductively coupled plasma-optical emission spectrometry	Not defined	Dominant lateral teeth											x	
				(26)	EDX	Not defined	Dominant lateral teeth				x				x		x		
			Emarginula fissura	(42)	Ashing, acid treatment, boiling, staining, refractive index, diffusion column	Whole radula	All						x				x	x	
				(43)	Ashing and acid treatment	Not defined, but probably whole radula	Not defined, but probably all					-						-	
		Calliostomatidae	*Calliostoma zizyphinum*	(42; termed *Trochus ziziphinus*)	Ashing, acid treatment, boiling, staining, refractive index, diffusion column	Whole radula	All						x				x	x	
				(43; termed *Calliostoma zizyphynum*)	Ashing and acid treatment	Not defined, but probably whole radula	Not defined, but probably all					-						-	
		Haliotidae	*Haliotis*	(42)	Ashing, acid treatment, boiling, staining, refractive index, diffusion column	Whole radula	All						x				x	x	
	Neritimorpha	Neritidae	*Nerita atramentosa*	(119)	EDS	Not defined	Dominant lateral tooth and marginal teeth				x	x		x	x	x	x	x	
	Patellogastropoda	Lepetidae	*Lepeta caeca*	(42)	Not defined	Not defined, but probably whole radula	Not defined, but probably all					x							
			Lepeta concentrica	(42; termed *Cryptobranchia concentrica*)	Not defined	Not defined, but probably whole radula	Not defined, but probably all					x							
		Lottiidae	*Nipponacmea schrenckii* (termed by Hua and Li: *Notoacmea schrenckii*)	(115)	EDX	Radular sack and maturation zone	Dominant lateral teeth					x						x	
			Patella ulyssiponensis	(43; termed *Patella athletica*)	Ashing and acid treatment	Whole radula	All			x	x	x	x		x	x	x	x	x
			Patelloida alticostata	(90)	Dissolving and ICP-AES	Whole radula	All			x	x	x	x	x		x	x	x	x
				(111)	EDX, Raman and infrared spectroscopies	Ontogeny	Dominant lateral teeth					x					x	x	
			Patelloida striata	(109; termed *Acmaea striata*)	X-ray diffraction	Ontogeny	Dominant lateral teeth					x						x	
			Tectura virginea	(42; termed *Acmaea virginea*)	Ashing, acid treatment, boiling, staining, refractive index, diffusion column	Whole radula	All					x							
			Lottia gigantea	(110)	EDX	Ontogeny	Dominant lateral teeth					x					x	x	
		Neolepetopsidae	*Paralepetopsis ferrugivora*	(112)	EDXA	Maturation zone	Dominant lateral teeth					x	x	x	x			x	
		Patellidae	*Patella pellucida*	(43; termed *Patella coeruleata* and *Patina pellucida*)	Ashing and acid treatment	Not defined, but probably whole radula	Not defined, but probably all					x						x	
												x						x	
			Patella rustica	(43; termed *Patella lusitanica*)	Ashing and acid treatment	Not defined, but probably whole radula	Not defined, but probably all					x						x	
			Scutellastra laticostata	(111)	EDX, Raman and infrared spectroscopies	Ontogeny	Dominant lateral teeth					x					x	x	
			Patella caerulea	(114)	Raman spectroscopy	Ontogeny	Dominant lateral teeth											x	
			Patella pellucida	(42)	Ashing, acid treatment, boiling, staining, refractive index, diffusion column	Whole radula	All				x	x	x				x	x	
			Patella vulgata	(105)	Scanning proton microprobe	Not defined	Dominant lateral teeth					x	x				x	x	x
				(43)	Ashing and acid treatment	Not defined, but probably whole radula	Not defined, but probably all					x						x	
				(107)	EDXA and acid treatment	Ontogeny	Dominant lateral teeth					x	x				x	x	
				(106)	Mössbauer spectroscopy and transmission electron microscopy	Ontogeny	Dominant lateral teeth											x	

				(103)	Ashing and acid treatment	Ontogeny	Dominant lateral teeth					x						x	
				(104)	Electron microprobe analyses	Ontogeny	Dominant lateral teeth					x		x				x	
				(42)	Ashing, acid treatment, boiling, staining, refractive index, diffusion column	Whole radula	All				x	x	x				x	x	
				(108)	EDX	Not defined	Lateral tooth cusp					x		x				x	
				(41)	Ashing and acid treatment	Whole radula	All						x				x	x	
				(113)	Atomic absorption spectrophotometer	Not defined	Not defined			x	x					x	x		
Polyplaco phora	Chitonida	Acanthochito nidae	*Acanthochitona discrepans*	(43; termed *Chiton discrepans*)	Ashing and acid treatment	Not defined, but probably whole radula	Not defined, but probably all					-						x	
			Cryptochiton stelleri	(23)	EDS	Working zone	Dominant lateral teeth						x					X	
				(81)	Electron microprobe analysis	Not defined	Dominant lateral teeth						x				x	x	
				(29)	EDS	Working zone	Dominant lateral teeth			x	x	x	x			x	x	x	
				(25)	EDS	Working zone	Dominant lateral teeth						x	x			x	x	
				(27)	EDS	Working zone	Dominant lateral teeth			x	x	x	x	x	x	x	x	x	
				(28)	EDS	Working zone	Dominant lateral teeth						x					x	
		Chaetopleuri dae	*Chaetopleura apiculata*	(88)	Atom probe tomography	Working zone	Dominant lateral teeth						x				x	x	
		Chitonidae	*Acanthopleura brevispinosa*	(85)	EDS	Ontogeny	Dominant lateral teeth											x	
			Acanthopleura curtisiana	(193)	Raman spectroscopy	Working zone	Dominant lateral teeth											x	
			Acanthopleura echinata	(92)	EDS	Ontogeny	Dominant lateral teeth				x		x	x			x	x	
				(145)	ESEM	Ontogeny	Dominant lateral teeth										x	x	
				(91)	EDS, Raman spectroscopy	Ontogeny	Dominant lateral teeth				x		x				x	x	
				(93)	EDS and Raman spectroscopy	Working zone	Dominant lateral teeth				x		x				x	x	
			Acanthopleura hirtosa	(102; termed *Clavarizona hirtosa*)	PIXE and PIGME	Ontogeny	Dominant lateral teeth		x	x			x	x	x	x	x	x	
				(101)	Fourier transform infrared spectroscopy	Working zone	Dominant lateral teeth		x								x		
				(82)	Ashing and histochemical staining	Ontogeny	Dominant lateral teeth						x				x	x	
				(84)	EDS and Raman spectroscopy	Ontogeny	Dominant lateral teeth						x				x	x	
				(90)	Dissolving and ICP-AES	Whole radula	All			x	x	x	x	x		x	x	x	x
				(162)	EDX	Ontogeny	Dominant lateral teeth						x				x	x	
				(87)	Backscatter, EDS, staining	Ontogeny	Dominant lateral teeth						x				x	x	
			Acanthopleura spinosa	(85)	EDS	Ontogeny	Dominant lateral teeth				x		x	x	x		x	x	
			Chiton mamoratus	(93)	EDS and Raman spectroscopy	Working zone	Dominant lateral teeth				x		x				x	x	

			Chiton stokesii	(89)	Mass spectrometry	Whole radula	All											x	
			Chiton tuberculatus	(89)	Mass spectrometry	Whole radula	All											x	
			Liolophura rehderi	(85; termed *Acanthopleura rehderi*)	EDS	Ontogeny	Dominant lateral teeth											x	
				(193; termed *Acanthopleura rehderi*)	Raman spectroscopy	Working zone	Dominant lateral teeth											x	
			Onithochiton quercinus	(85)	EDS	Ontogeny	Dominant lateral teeth				x		x	x	x		x	x	
				(193)	Raman spectroscopy	Working zone	Dominant lateral teeth											x	
				(93)	EDS and Raman spectroscopy	Working zone	Dominant lateral teeth				x		x				x	x	
			Rhyssoplax olivacea	(194; termed *Chiton olivaceus*)	X-ray microanalysis	Not defined, but probably working zone	Dominant lateral teeth											x	
			Squamopleura miles	(93; termed *Acanthopleura miles*)	EDS and Raman spectroscopy	Working zone	Dominant lateral teeth				x		x				x	x	
			Sypharochiton pelliserpentis	(100; termed *Chiton pelliserpentis*)	FT-IR, Raman, XRD	Not defined	Dominant lateral teeth										x		
				(93)	EDS and Raman spectroscopy	Working zone	Dominant lateral teeth				x		x				x	x	
		Cryptoplacidae	*Cryptoplax striata*	(83)	EDS	Ontogeny	Dominant lateral teeth			x	x	x	x	x		x	x	x	
		Ischnochitonidae	*Ischnochiton australis*	(85)	EDS	Ontogeny	Dominant lateral teeth				x		x	x	x		x	x	
				(93)	EDS and Raman spectroscopy	Working zone	Dominant lateral teeth				x		x				x	x	
		Mopaliidae	*Mopalia muscosa*	(89)	Mass spectrometry	Whole radula	All											x	
			Plaxiphora albida	(85)	EDS	Ontogeny	Dominant lateral teeth				x		x	x	x		x	x	
				(86)	Raman spectroscopy	Not defined	Dominant lateral teeth					x	x				x	x	
				(93)	EDS and Raman spectroscopy	Working zone	Dominant lateral teeth				x		x				x	x	
				(90)	Dissolving and ICP-AES	Whole radula	All			x	x	x	x	x		x	x	x	x
		Tonicellidae	*Tonicella lineata*	(89)	Mass spectrometry	Whole radula	All											x	
			Tonicella marmorea	(89)	Mass spectrometry	Whole radula	All											x	
	?	?	*Chiton emarginata*	(43)	Ashing and acid treatment	Not defined, but probably whole radula	Not defined, but probably all					-						x	
	?	?	*Chiton olivarius*	(43)	Ashing and acid treatment	Not defined, but probably whole radula	Not defined, but probably all					-						x	
	?	?	*Craspedocheilus cinereus*	(43)	Ashing and acid treatment	Not defined, but probably whole radula	Not defined, but probably all					-						x	
Scaphopoda	Dentalida	Dentaliidae	*Dentalium spec.*	(43)	Ashing and acid treatment	Not defined, but probably whole radula	Not defined, but probably all					x						x	
Cephalopoda	Octopoda	Argonautidae	*Argonauta spec.*	(43)	Ashing and acid treatment	Not defined, but probably whole radula	Not defined, but probably all					-						-	
